# Diborane(4) Azides: Surprisingly Stable Sources of Transient Iminoboranes

**DOI:** 10.1002/anie.202003050

**Published:** 2020-05-28

**Authors:** Torsten Thiess, Guillaume Bélanger‐Chabot, Felipe Fantuzzi, Maximilian Michel, Moritz Ernst, Bernd Engels, Holger Braunschweig

**Affiliations:** ^1^ Institute for Inorganic Chemistry and Institute for Sustainable Chemistry & Catalysis with Boron Julius-Maximilians-Universität Würzburg Am Hubland 97074 Würzburg Germany; ^2^ Département de Chimie Université Laval 1045 Avenue de la Médecine Québec G1V 0A6 Canada; ^3^ Institute for Physical and Theoretical Chemistry Julius-Maximilians-Universität Würzburg Emil-Fischer-Straße 42 97074 Würzburg Germany

**Keywords:** azides, boron nitride, diazadiboretidine, diborane(4), nitrenes

## Abstract

Herein we describe the first examples of isolable electron‐precise diboranes(4) that bear azide moieties: the acyclic 1,2‐diazido‐1,2‐bis(dimethylamino)diborane(4) and the cyclic 1,4‐diaryl‐2,3‐diazido‐1,4‐diaza‐2,3‐diborinines (aryl=mesityl, 2,6‐xylyl, 4‐tolyl). The reported examples are not only stable enough to be observed and isolated (putative transient diborane(4) azides previously reported by our group spontaneously decompose even below room temperature), but some of them are even robust enough to undergo controlled pyrolysis without explosive decomposition at temperatures well above 100 °C. In two cases, the controlled pyrolysis allows the isolation of complex diazaboretidines, which are the apparent dimerization products of endocyclic boryl‐iminoboranes.

## Graphical abstract

**Potentially explosive yet mild‐mannered**! The synthesis and reactivity of diamino‐substituted diborane(4) diazides, the first diborane(4) azides, is reported. Despite the presence of energetic and oxidizing azide groups and of reactive B−B bonds, they are astonishingly robust. The species do not decompose below 100 °C. Two diazides could be pyrolyzed in a controlled manner to diazadiboretidines, the dimerization product of transient iminoboranes.

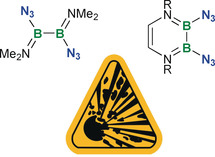

Azidoboranes are of significant interest both as synthetic intermediates and as energetic materials.^1^ Indeed, mono‐ and diazidoboranes are useful precursors to iminoboranes,^2^ which themselves take part in interesting reactivity with organic and organoboron azides (Figure 1). While B(N_3_)_3_ likely only exists in the gas phase,^3^ many of its adducts are known.^1^ [B(N_3_)_4_]^−^ is also known^1^, ^4^ and is a highly energetic anion that can yield spectacularly nitrogen‐rich salts.^5^ The decomposition of binary boron azides can yield boron nitrides, which makes the general field of boron azides relevant to materials chemistry.3a, 3b, 6 In stark contrast to the rather well‐established chemistry of monoborane azides, there has been, to our knowledge, no explicit report on the existence of electron‐precise diborane(4) azide species.

**Figure 1:**
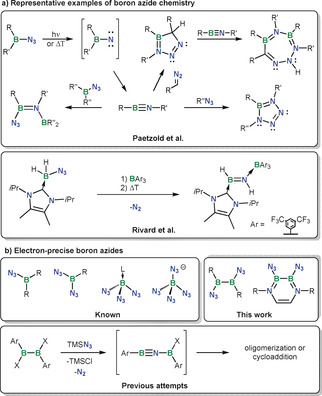
a) Representative examples of boron azide chemistry. b) Electron‐precise boron azides.

Our group has recently endeavored to revisit the chemistry of binary diborane(4) halides and their derivatives,^7^ which are related to important substrates in borylation catalysis.^8^ Beyond binary boron‐halogen species,^9^ the next logical step in developing the systematic chemistry of diboranes(4) is to explore the chemistry of pseudohalide derivatives. Only hexakis(pseudohalido)diborates(6) (pseudohalide=CN^−^, NCS^−^)^10^ and a few mixed derivatives bearing a cyanide group are known.^11^ The lack of known diborane(4) azides stands out, particularly because of the synthetic potential of the azide functionality,^12^ its rather high reactivity, which has yielded intriguing boron compounds in related systems,^13^ and its energetic character.

Our recent work^14^ on the attempted synthesis of 1,2(diaryl)‐diborane(4) azide derivatives indicates that such compounds tend to be short‐lived and spontaneously eliminate N_2_, giving rise to a chemistry that is best described as oligomerization of previously unknown boryl‐substituted iminoboranes (Figure 1). Attempts at forming diborane(4) azides from tetrahalodiboranes(4) and their derivatives showed even less controllable decomposition reactions that have so far only yielded intractable mixtures, all of which are indicative of the breakage of the B−B bond to a large extent in the products (see Supporting Information). The predisposition of diborane(4) azides to form transient nitrene‐like species was hypothesized to be due to the intramolecular activation of the azide,^14^ by analogy with borane‐triggered decompositions of some azides.13f, 13g, 13i A reasonable strategy to hinder such intramolecular activation would be the saturation of the empty p orbital at boron through π‐donation, for example with amine functional groups. Amine functional groups are already known to stabilize various reactive boron‐containing motifs,^15^ and diboranes(4) B_2_(NMe_2_)_4_ and B_2_(NMe_2_)_2_X_2_ are significantly more stable than the parent B_2_X_4_ (X=halide) species.

Our group's experience with the surprising results of combining azides with reactive boron compounds13m, 13n, 13p, 16 prompted us to verify whether the stabilization provided by amine groups would allow us to isolate diboranes(4) bearing at least one azide group and whether this stabilization would prevent all further reactivity. Herein we report on the synthesis of several diazidodiboranes and their use as precursors to transient boryl‐iminoboranes, which yielded unprecedented dimers of NBNBN‐containing seven‐membered rings.

The simple treatment of 1,2‐dichloro‐1,2‐bis(dimethylamino)diborane(4) with trimethylsilyl azide (TMSN_3_) in dichloromethane or benzene at room temperature cleanly afforded a single new species with a ^11^B NMR signal at slightly higher field than the starting material (*δ*(^11^B)=34.8 ppm) (Scheme 1). The new compound was isolated as a pale‐yellow liquid (46 mol %) by repeated fractional condensation. Its mass spectrum indicated its identity as the diazidodiborane(4) **1**. The presence of only one signal in the ^11^B NMR spectrum of **1** strongly supports its assignment as 1,2‐diazido‐1,2‐bis(dimethylamino)diborane(4). The IR spectrum of **1** features a strong band at 2112 cm^−1^, also consistent with the presence of an azide group covalently attached to boron (the ν_asym_ N_3_ band in covalently bound azides is usually observed at ca. 2100 cm^−1^)^17^ and in good agreement with predicted frequencies (see Supporting Information). **1** is also formed when an azide salt, [PPh_4_]N_3_, is used as the azide source. Since no [PPh_4_]Cl precipitates, the reaction appears to be mostly driven by the higher stability of the B−N_3_ bond with respect to the B−Cl bond in this system.

**Scheme 1:**
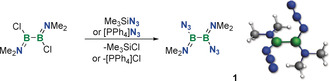
Synthesis of diborane(4) diazide **1** (right: lowest‐energy conformer optimized at the B3LYP/6‐31+G* level).

In solution at room temperature, **1** is surprisingly stable considering its reactive B−B bond and azido substituents combined within one molecule. Indeed, it can be heated to 80 °C for days with only marginal decomposition. Attempts to increase the degree of azide substitution towards B_2_(N_3_)_4_ by displacing the NMe_2_ groups only led (slowly) to decomposition, the products of which were indicative of the cleavage of the B−B bond (with ^11^B NMR signals appearing at ca. 0 ppm, which are uncharacteristic of diboranes(4)). **1** is a rather small molecule with a large proportion of nitrogen (ca. 58 % by weight) and is therefore likely to behave explosively.^18^ The compound has not detonated nor deflagrated under normal handling conditions, but it did deflagrate with a green flame when confined and heated with the flame of a butane torch. Chelating amines with large organic substituents can provide significant steric crowding and as such are expected to yield kinetically very robust diboranes(4) and to decrease the sensitivity of the derived diborane(4) diazides,^18^ rendering them much safer to use on a synthetic scale. Dilithiated 1,4‐diazabutadiene derivatives are ideally suited for that strategy. Indeed, when reacted with diborane precursors, they are known to yield 1,4‐diaza‐2,3‐diborinines,^19^ which are BN isosteres of benzene, with the concomitant possibility of additional resonance stabilization.^20^ These systems have been known to allow the isolation of unusual diborane(4)‐type compounds.^21^

Much like in the formation of **1**, the treatment of 2,3‐dihalodiazadiborinine derivatives19e with an excess of trimethylsilyl azide in benzene cleanly yielded the diazidodiboranes(4) **2** (Figure 2). The reaction proceeded to completion at room temperature overnight using dibromodiazadiborinine precursors for **2 a** and **2 b** but only the dichlorodiazadiborinine precursor yielded **2 c** without decomposition under similar conditions. This is consistent with the fact that the *p*‐tolyl substituent in **2 c** provides much less steric crowding in the periphery of the boron centers, thus allowing various unidentified reactions. This is not specific to the diazide **2 c** but is characteristic of the dibromodiazadiborinine starting material. Indeed, during the isolation of the dibromodiazadiborinine starting material, its significantly higher reactivity, leading to increased side‐reactions compared to the dichloro analogue, was also observed.19e The ^11^B NMR signals for **2** (single resonances at *δ*(ppm) 34.0 (**2 a**); 33.8 (**2 b**); 33.5 (**2 c**)) show very little influence of changing the substituent, also when compared against the signal for **1**.

**Figure 2:**
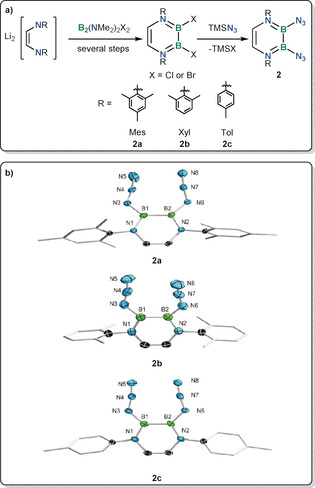
a) Synthesis of diborane(4) azides. b) Crystallographically‐derived structures of **2**.^31^ Hydrogen atoms and peripheral carbon ellipsoids omitted for clarity. Selected bond distances (Å) and angles (°). **2 a**: B1–B2 1.670(4), B1–N1 1.419(3), B2–N2 1.414(3), B1–N3 1.468(4), B2–N6 1.461(3) N3–N4 1.218(3), N4–N5 1.130(4), N6–N7 1.230(3), N7–N8 1.133(3), N3‐N4‐N5 171.4(3), N6‐N7‐N8 172.0(3) (only one of the two crystallographically‐independent molecules displayed, see Supporting Information); **2 b**: B1–B2 1.676(2), B1–N1 1.413(2), B2–N2 1.415(2) (disorder model for the azide groups of **2 b** is not depicted for clarity, see Supporting Information). **2 c**: B1–B2 1.682(4), B1–N1 1.422(4), B2‐N2 1.423(4), B1–N3 1.459(4), B1–N6 1.455(4) N3–N4 1.229(4), N4–N5 1.143(4), N6–N7 1.233(3), N7–N8 1.140(4), N3‐N4‐N5 172.0(3), N6‐N7‐N8 170.4(3) (only one of the two crystallographically‐independent molecules displayed).

As expected for most azaborinines, the rings in **2** are essentially planar (Figure 2). The B−B bond lengths are in the range found for most diboranes(4) and diboranes(6).7a The endocyclic B−N bond distances of **2 a**, **2 b** and **2 c** (ca. 1.41 Å) are all statistically identical. The azide groups are oriented approximately in the azaborinine plane, allowing a π‐interaction between the α‐nitrogen atom of the azide group and the boron to which it is attached, which is reflected in the exocyclic B−N distances of B1−N3 1.468(4), B2−N6 1.461(3) Å (**2 a**), B1−N3 1.459(4), B1−N6 1.455(4) Å (**2 c**). The structural parameters for the azide groups are typical of other covalently bound azide groups: B−N_azide_ distance of ca. 1.46 Å (indicative of some B−N_α_ π‐interaction), N_α_−N_β_ 1.22 Å and N_β_−N_γ_ 1.12 Å and a slightly non‐linear N_α_N_β_N_γ_ bond angle of ca. 172°.^17^ The azide groups all point towards each other, except in one of the two crystallographically‐independent molecules of the less sterically crowded **2 c** (see Supporting Information). The IR spectra of **2 a,b,c** each feature a strong band between 2133 and 2139 cm^−1^ (with, in each case, a poorly resolved shoulder), consistent with the presence of a covalent azide and in good agreement with predicted frequencies (see Supporting Information). NICS calculations on **2 a** as a model compound hints at an aromatic character for compounds **2** (see Supporting Information).

With a selection of unprecedented diborane(4) diazides in hand, the reaction chemistry of (formal) transient nitrenes was the next logical step for this study. The photolysis of **1** led to its complete conversion within 24 h, yielding species displaying one major feature at ca. 25 ppm in the ^11^B NMR spectrum as well as one broad complex resonance at 2.56 ppm in the ^1^H NMR spectrum. Clear evidence for dinitrogen elimination was found by ^14^ N NMR spectroscopy, with a sharp signal at −71 ppm corresponding to free N_2_. These spectroscopic observations hint at the presence of a mixture of oligomers which remains thus far intractable. This is not unexpected, as **1** is a rather small molecule which can be conceived to undergo unhindered oligomerization reactions, especially if two transient formal nitrenes are simultaneously formed on a single molecule. An even less selective reaction was observed for the similarly unencumbered **2 c**. The pyrolysis of **1** in mesitylene is similarly unselective but, strikingly, takes days to reach completion at temperatures between 120 and 150 °C, again showing the rather high stability of **1** compared to transient diborane azides that have likely been generated but thus far have not been observed.^14^

The bulkier diazides **2 a** and **2 b**, which provide more steric shielding at the boron centers, proved to be much better substrates for the selective formation of pyrolysis products. The pyrolysis above 150 °C (neat or in mesitylene, see Supporting Information) afforded compounds **3 a** and **3 b**, respectively, which were shown via X‐ray diffraction‐derived solid‐state structures to be diazadiboretidines (Figure 3). These new species are the apparent dimerization products of transient, endocyclic iminoboranes. These iminoboranes are rare examples of borylated iminoboranes^14^, ^22^ and the first examples of borylimino(amino)boranes. Indeed, while borepines are not uncommon,^23^ seven‐membered rings bearing the NBNBN sequence have, to our knowledge, never been reported, although related seven‐membered boron‐nitrogen‐containing heterocycles have been reported in the context of ring‐expansion reactions of boroles13o, 24 and in carbene‐diazadiborinine adducts.21d Our seven‐membered rings are also closely related to a six‐membered cyclic iminoborane reported by Bettinger et al., which likewise displays a behavior typical of iminoboranes (oligomerization, cycloaddition, etc.).13j

**Figure 3:**
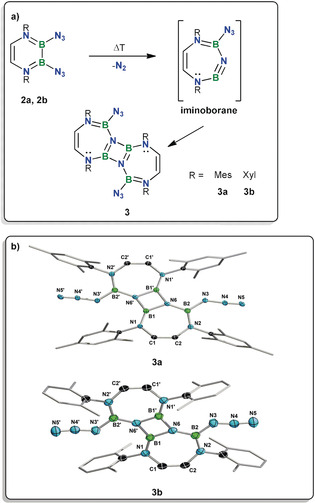
a) The pyrolysis of **2 b** and **2 c** yields the novel diazadiboretidines **3 a** and **3 b**, the apparent products of the dimerization of *endo*‐cyclic transient iminoboranes. b) Crystallographically‐derived structures of **3 a** and **3 b**.^31^ Hydrogen atoms and peripheral carbon ellipsoids were omitted for clarity. Selected structural parameters. **3 a**: Bond distances (Å) and angles (°): B1–N6 1.469(2), B1–N6′ 1.463(2), B1–N1 1.397(2), B2–N6 1.421(2), B2–N2 1.421(2), N6′‐B1‐N6 96.4(1), B1‐N6‐B1′ 83.6(1). Angle between the B1‐N6‐B1′ and B2‐N2‐C2 planes: 21.41°. Angle between the B1‐N6‐B1′ and N1‐C1‐C2 planes: 14.26°. The disorder models for the azide groups are not depicted for clarity (see Supporting Information). **3 b**: Bond distances (Å) and angles (°): B1–N6 1.472(2), B1–N6′ 1.462(2), B1–N1 1.399(2), B2–N6 1.423(2), B2–N2 1.423(2), B2–N3 1.457(3), N3–N4 1.212(2), N4–N5 1.125(2), N6′‐B1‐N6 96.5(1), B1‐N6‐B1′ 83.5(1), N3‐N4‐N5 171.0(2). Angle between the B1‐N6‐B1′ and B2‐N2‐C2 planes: 19.54°. Angle between the B1‐N6‐B1′ and N1‐C1‐C2 planes: 15.22°. **3 a** and **3 b** lie on a crystallographic inversion center and the full molecules are generated by symmetry.

The IR spectra of **3 a,b** each feature a strong band between 2138 and 2141 cm^−1^, consistent with the presence of residual azide groups, which is also found in the solid‐state structures of **3 a,b**. The presence of azide substituents in the pyrolysis products is noteworthy and is a strong indication of the surprising stability of the azido(amino)boron moiety. The diazadiboretidines **3** are part of a limited group of structurally characterized diazadiboretidines.2h, 2j, 2p, 2q, 2r, 2t, 2v, 2x, 25 The structural data obtained for **3 a,b** via X‐ray crystallography shows that the seven‐membered rings deviate from planarity, as expected for molecules related to cycloheptatrienes. The B−N distances are all similar in **3 a** and **3 b**, ranging from 1.397(2) to 1.469(2) Å (**3 a**) and from 1.399(2) to 1.472(2) Å (**3 b**). The shortest B−N bond in **3** is the B1−N1 bond. The diazadiboretidine ring is essentially planar in both compounds.

Products **3 a,b** are the dimers of putative seven‐membered cyclic iminoborane analogues that are remarkably similar to the products of the photolysis of aryl azides (yielding transient nitrenes) in the presence of nucleophiles, which ultimately result in azepines.^26^ Our recently reported attempts at generating diborane(4) azides showed that some of these putative compounds spontaneously undergo loss of dinitrogen and yield nitrene‐like reaction patterns without requiring the presence of a nitrene intermediate.^14^ This prompted investigations into the mechanistic aspects of the transformations yielding **3 a** and **3 b**.

We conducted quantum chemical calculations on the reactions forming **3 a,b** at the B3LYP(D3)/6‐311++G**+SMD(Mesitylene)//B3LYP/6‐31+G* level of theory^27^ together with high‐level CASSCF^28^ and NEVPT2^29^ calculations. Our results show that the triplet state of a diborane‐substituted nitrene is more stable than the singlet biradical state by 8.8 kcal mol^−1^ at the NEVPT2/cc‐pVDZ//B3LYP/6‐31+G* level.^30^ Interestingly, the singlet state was shown to have a significant biradical character, as revealed by both DFT and high‐level CASSCF/NEVPT2 calculations (see Supporting Information). We compared two types of reaction pathways for the formation of **3** using **3 a** as a model compound. One involves a nitrene intermediate (see Supporting Information) while the other involves the loss of dinitrogen from an azide group without the intermediacy of a nitrene species (Scheme 2). We found that the loss of dinitrogen, the limiting step of both mechanisms, was more favorable for the “nitreneless” mechanism by 9 kcal mol^−1^ (activation barriers: nitrene pathway 42 kcal mol^−1^; nitreneless pathway 33 kcal mol^−1^). This indicates that it is unlikely that a nitrene is involved in reactions leading to **3** but rather that the loss of dinitrogen occurs in concert with the insertion of the remaining nitrene‐like nitrogen atom originating from the azide group into the boron–boron bond (Scheme 2). The full nitreneless pathway is outlined in Scheme 2. The elimination of dinitrogen (**TS2**) is preceded by a *cis*–*trans* isomerization of the B_2_(N_3_)_2_ moiety via an azide rotation about the B−N bond with a low free energy barrier of 4.9 kcal mol^−1^ (**TS1**). The loss of dinitrogen and simultaneous nitrogen insertion into the B−B bond, which yields the endocyclic imiborane **2 a′′**, occurs with a barrier of 33 kcal mol^−1^, in qualitative agreement with the reaction occurring at rather high temperatures (150 °C and above). The last step is the very exergonic dimerization of the resulting transient iminoborane, yielding compound **3 a**. Although the putative triplet‐state nitrene does not appear to play a significant role in the reactions observed in this study, our computational work suggests that such diborane‐nitrene intermediate could lead to intriguing species, for example their dimerization product, an azo‐bridged bis(diazadiborinine) species **4** (Scheme 3).

**Scheme 2:**
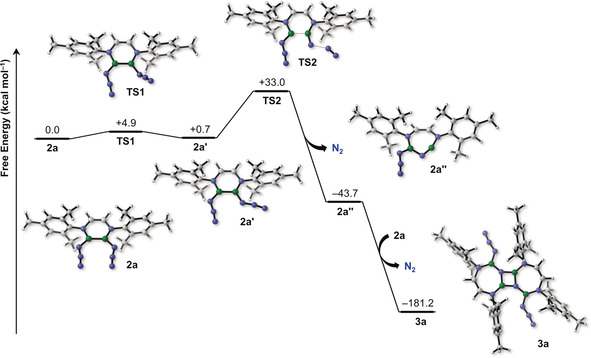
Computed reaction mechanism (B3LYP(D3)/6‐311++G**+SMD(Mesitylene)) of the dimerization of diazidodiborane through an iminoborane intermediate.

**Scheme 3:**
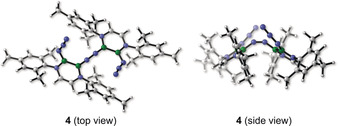
Hypothetical azo‐bridged bis(diazadiborinine) species **4** at the B3LYP/6‐31+G* level of theory.

In conclusion, we report the first isolable diborane(4) azides. Alongside our previous work,^14^ these results suggest that the formation of a nitrene‐like moiety and its insertion into a B−B bond tend to be the most accessible decomposition pathway of diborane(4) azides. The fact that temperatures well above 100 °C are required to decompose compounds **1** and **2** strongly supports our hypothesis that the electronic saturation of the boron centers in diborane(4) azides, provided in this study by amine substituents at boron, should lead to significant stabilization. The diazides synthesized in this work were in fact so stable that the most sterically congested ones could be pyrolyzed without explosive decomposition, yielding putative transient endocyclic boryl‐iminoboranes. The latter dimerize into complex polycyclic boron‐nitrogen diazadiboretidines. The isolated diborane(4) azides are not only an important step towards developing the inorganic chemistry of diboranes(4), but also provide convenient synthons that could allow the introduction of the B−B moiety to other molecules via azide‐mediated reactions, such as ring‐expansions and cycloadditions. The stability of compound **1** also warrants further work into the exploration of high energy density materials (HEDM) based on diborane azides. On a more general note, azides are convenient precursors for the generation of metal and non‐metal nitrides. We demonstrate herein that some of the diborane(4) diazides can undergo pyrolysis in a remarkably controllable fashion, to the extent that one azide group remains after high‐temperature pyrolysis of diazides **2 a** and **2 b**. The presence of a boron–boron bond in the precursors is expected to change the dynamics of formation of extended structure materials, such as boron nitrides. Diborane(4) azides could therefore act as models for precursors to new hydrocarbon‐rich boron nitride analogues.^6^

## Experimental Section

**Caution**! The materials in this study bear energetic azide groups and therefore can behave explosively. While no inadvertent deflagration or detonation occurred throughout this work, the materials discussed should be assumed to be potentially explosive and handled on millimolar scales or below using appropriate protective equipment, including face shield, heavy leather gloves and jacket, and earplugs. Pyrolysis in the absence of solvents should be avoided when possible and conducted using minute amounts of starting materials and behind blast shields (see Supporting Information).

## Conflict of interest

The authors declare no conflict of interest.

## Supporting information

As a service to our authors and readers, this journal provides supporting information supplied by the authors. Such materials are peer reviewed and may be re‐organized for online delivery, but are not copy‐edited or typeset. Technical support issues arising from supporting information (other than missing files) should be addressed to the authors.

Supplementary

## References

[1] W. Fraenk , T. Habereder , A. Hammerl , T. M. Klapötke , B. Krumm , P. Mayer , H. Nöth , M. Warchhold , Inorg. Chem. 2001, 40, 1334–1340. 11300838 10.1021/ic001119b

[2a] T. Mennekes , P. Paetzold , Z. Anorg. Allg. Chem. 1995, 621, 1175–1177;

[2b] H. F. Bettinger , M. Filthaus , H. Bornemann , I. M. Oppel , Angew. Chem. Int. Ed. 2008, 47, 4744–4747; 10.1002/anie.200705936 18481829

[2c] P. I. Paetzold , G. Stohr , Chem. Ber. 1968, 101, 2874–2880;

[2d] P. Paetzold , C. von Plotho , Chem. Ber. 1982, 115, 2819–2825;

[2e] P. Paetzold , R. Truppat , Chem. Ber. 1983, 116, 1531–1539;

[2f] K. Delpy , H.-U. Meier , P. Paetzold , C. von Plotho , Z. Naturforsch. B 1984, 39, 1696–1701;

[2g] H.-U. Meier , P. Paetzold , E. Schröder , Chem. Ber. 1984, 117, 1954–1964;

[2h] P. Paetzold , C. V. Plotho , G. Schmid , R. Boese , B. Schrader , D. Bougeard , U. Pfeiffer , R. Gleiter , W. Schüfer , Chem. Ber. 1984, 117, 1089–1102;

[2i] P. Paetzold , C. von Plotho , G. Schmid , R. Boese , Z. Naturforsch. B 1984, 39, 1069–1075;

[2j] P. Paetzold , E. Schröder , G. Schmid , R. Boese , Chem. Ber. 1985, 118, 3205–3216;

[2k] K.-H. van Bonn , T. von Bennigsen-Mackiewicz , J. Kiesgen , C. von Plotho , P. Paetzold , Z. Naturforsch. B 1988, 43, 61–68;

[2l] P. Paetzold , E. Eleftheriadis , R. Minkwitz , V. Wölfel , R. Gleiter , P. Bischof , G. Friedrich , Chem. Ber. 1988, 121, 61–66;

[2m] B. Thiele , P. Schreyer , U. Englert , P. Paetzold , R. Boese , B. Wrackmeyer , Chem. Ber. 1991, 124, 2209–2216;

[2n] J. Kiesgen , J. Münster , P. Paetzold , Chem. Ber. 1993, 126, 1559–1563;

[2o] B. Kröckert , K.-H. van Bonn , P. Paetzold , Z. Anorg. Allg. Chem. 2005, 631, 866–868;

[2p] P. Paetzold , A. Richter , T. Thijssen , S. Würtenberg , Chem. Ber. 1979, 112, 3811–3827;

[2q] P. Paetzold , C. von Plotho , G. Schmid , R. Boese , Z. Naturforsch. B 1984, 39, 1069–1075;

[2r] G. Schmid , D. Kampmann , W. Meyer , R. Boese , P. Paetzold , K. Delpy , Chem. Ber. 1985, 118, 2418–2428;

[2s] P. Paetzold , in Advances in Inorganic Chemistry, Vol. 31 (Eds.: H. J. Emeléus, A. G. Sharpe), Academic Press, San Diego, 1987, pp. 123–170;

[2t] P. Paetzold , K. Delpy , R. Boese , Z. Naturforsch. B 1988, 43, 839–845;

[2u] P. Schreyer , P. Paetzold , R. Boese , Chem. Ber. 1988, 121, 195–205;

[2v] P. Paetzold , B. Redenz-Stormanns , R. Boese , M. Bühl , P. von Ragué Schleyer , Angew. Chem. Int. Ed. Engl. 1990, 29, 1059–1060;

[2w] F. Meyer , P. Paetzold , U. Englert , Chem. Ber. 1992, 125, 2025–2026;

[2x] S. Luckert , E. Eversheim , M. Müller , B. Redenz-Stormanns , U. Englert , P. Paetzold , Chem. Ber. 1995, 128, 1029–1035;

[2y] E. Bulak , T. Varnal , P. Paetzold , U. Englert , Z. Anorg. Allg. Chem. 1999, 625, 3–5;

[2z] S. Luckert , E. Eversheim , U. Englert , T. Wagner , P. Paetzold , Z. Anorg. Allg. Chem. 2001, 627, 1815–1823.

[3a] R. L. Mulinax , G. S. Okin , R. D. Coombe , J. Phys. Chem. 1995, 99, 6294–6300;

[3b] I. A. Al-Jihad , B. Liu , C. J. Linnen , J. V. Gilbert , J. Phys. Chem. A 1998, 102, 6220–6226;

[3c] F. Liu , X. Zeng , J. Zhang , L. Meng , S. Zheng , M. Ge , D. Wang , D. Kam Wah Mok , F.-t. Chau , Chem. Phys. Lett. 2006, 419, 213–216;

[3d] E. Wiberg , H. Michaud , Z. Naturforsch. B 1954, 9, 497.

[4] W. Fraenk , H. Nöth , T. M. Klapötke , M. Suter , Z. Naturforsch. B 2002, 57, 621.

[5] R. Haiges , S. Schneider , T. Schroer , K. O. Christe , Angew. Chem. Int. Ed. 2004, 43, 4919–4924; 10.1002/anie.200454242 15372568

[6] R. H. Wentorf , J. Chem. Phys. 1962, 36, 1990–1991.

[7a] G. Bélanger-Chabot , H. Braunschweig , Angew. Chem. Int. Ed. 2019, 58, 14270–14274; 10.1002/anie.201906666 PMC7028027 31361383

[7b] M. Arrowsmith , J. Böhnke , H. Braunschweig , A. Deißenberger , R. D. Dewhurst , W. C. Ewing , C. Hörl , J. Mies , J. H. Muessig , Chem. Commun. 2017, 53, 8265–8267; 10.1039/c7cc03148c 28656182

[7c] L. Englert , A. Stoy , M. Arrowsmith , J. H. Müssig , M. Thaler , A. Deißenberger , A. Häfner , J. Böhnke , F. Hupp , J. Seufert , J. Mies , A. Damme , T. Dellermann , K. Hammond , T. Kupfer , K. Radacki , T. Thiess , H. Braunschweig , Chem. Eur. J. 2019, 25, 8612–8622. 30974025 10.1002/chem.201901437

[8] E. C. Neeve , S. J. Geier , I. A. I. Mkhalid , S. A. Westcott , T. B. Marder , Chem. Rev. 2016, 116, 9091–9161. 27434758 10.1021/acs.chemrev.6b00193

[9a] M. E. Peach , T. C. Waddington , J. Chem. Soc. A 1968, 180–182;

[9b] W. C. Schumb , E. L. Gamble , M. D. Banus , J. Am. Chem. Soc. 1949, 71, 3225–3229;

[9c] T. Wartik , R. Moore , H. I. Schlesinger , J. Am. Chem. Soc. 1949, 71, 3265–3266;

[9d] G. Urry , T. Wartik , R. E. Moore , H. I. Schlesinger , J. Am. Chem. Soc. 1954, 76, 5293–5298;

[9e] A. Finch , H. I. Schlesinger , J. Am. Chem. Soc. 1958, 80, 3573–3574;

[9f] W. Haubold , P. Jacob , Z. Anorg. Allg. Chem. 1983, 507, 231–234.

[10a] W. Preetz , B. Steuer , Z. Naturforsch. B 1996, 51, 551;

[10b] J. Landmann , J. A. P. Sprenger , M. Hailmann , V. Bernhardt-Pitchougina , H. Willner , N. Ignat′ev , E. Bernhardt , M. Finze , Angew. Chem. Int. Ed. 2015, 54, 11259–11264; 10.1002/anie.201504579 26219926

[11] S. C. Malhotra , Inorg. Chem. 1964, 3, 862–865.

[12] S. Bräse , K. Banert , Organic Azides. Syntheses and Applications, Wiley, Chichester, 2010.

[13a] R. L. Melen , A. J. Lough , D. W. Stephan , Dalton Trans. 2013, 42, 8674–8683; 23652382 10.1039/c3dt50791b

[13b] R. L. Melen , D. W. Stephan , Dalton Trans. 2013, 42, 4795–4798; 23436044 10.1039/c3dt00068k

[13c] C. M. Mömming , G. Kehr , B. Wibbeling , R. Fröhlich , G. Erker , Dalton Trans. 2010, 39, 7556–7564; 20617234 10.1039/c0dt00015a

[13d] A. Stute , G. Kehr , R. Fröhlich , G. Erker , Chem. Commun. 2011, 47, 4288–4290; 10.1039/c1cc10241a 21365106

[13e] A. Stute , L. Heletta , R. Fröhlich , C. G. Daniliuc , G. Kehr , G. Erker , Chem. Commun. 2012, 48, 11739–11741; 10.1039/c2cc36782c 23111350

[13f] A. K. Swarnakar , C. Hering-Junghans , M. J. Ferguson , R. McDonald , E. Rivard , Chem. Sci. 2017, 8, 2337–2343; 28451338 10.1039/c6sc04893e PMC5365008

[13g] K. Bläsing , J. Bresien , R. Labbow , D. Michalik , A. Schulz , M. Thomas , A. Villinger , Angew. Chem. Int. Ed. 2019, 58, 6540–6544; 10.1002/anie.201902226 30888089

[13h] E. Merling , V. Lamm , S. J. Geib , E. Lacôte , D. P. Curran , Org. Lett. 2012, 14, 2690–2693; 22616557 10.1021/ol300851m

[13i] A. K. Swarnakar , C. Hering-Junghans , K. Nagata , M. J. Ferguson , R. McDonald , N. Tokitoh , E. Rivard , Angew. Chem. Int. Ed. 2015, 54, 10666–10669; 10.1002/anie.201504867 26214271

[13j] M. Müller , C. Maichle-Mössmer , H. F. Bettinger , Angew. Chem. Int. Ed. 2014, 53, 9380–9383; 10.1002/anie.201403213 25044930

[13k] M. Müller , C. Maichle-Mössmer , H. F. Bettinger , J. Org. Chem. 2014, 79, 5478–5483; 24865178 10.1021/jo500549m

[13l] H. Braunschweig , K. Geetharani , J. O. C. Jimenez-Halla , M. Schäfer , Angew. Chem. Int. Ed. 2014, 53, 3500–3504; 10.1002/anie.201309707 24574145

[13m] H. Braunschweig , C. Hörl , L. Mailänder , K. Radacki , J. Wahler , Chem. Eur. J. 2014, 20, 9858–9861; 24964998 10.1002/chem.201403101

[13n] H. Braunschweig , M. Celik , T. Dellermann , G. Frenking , K. Hammond , F. Hupp , H. Kelch , I. Krummenacher , F. Lindl , L. Mailänder , J. Müssig , A. Ruppert , Chem. Eur. J. 2017, 23, 8006–8013; 28430374 10.1002/chem.201700749

[13o] S. A. Couchman , T. K. Thompson , D. J. D. Wilson , J. L. Dutton , C. D. Martin , Chem. Commun. 2014, 50, 11724–11726; 10.1039/c4cc04864d 25142866

[13p] F. Lindl , S. Lin , I. Krummenacher , C. Lenczyk , A. Stoy , M. Müller , Z. Lin , H. Braunschweig , Angew. Chem. Int. Ed. 2019, 58, 338–342; 10.1002/anie.201811601 30394650

[14] D. Prieschl , G. Belanger-Chabot , X. Guo , M. Dietz , M. Muller , I. Krummenacher , Z. Lin , H. Braunschweig , J. Am. Chem. Soc. 2020, 142, 1065–1076. 31830413 10.1021/jacs.9b12336

[15] H. Nöth , J. Knizek , W. Ponikwar , Eur. J. Inorg. Chem. 1999, 1931–1937.

[16] H. Braunschweig , M. A. Celik , F. Hupp , I. Krummenacher , L. Mailänder , Angew. Chem. Int. Ed. 2015, 54, 6347–6351; 10.1002/anie.201500970 25808881

[17] W. Fraenk , T. Habereder , T. M. Klapötke , H. Nöth , K. Polborn , J. Chem. Soc. Dalton Trans. 1999, 4283–4286.

[18] H. C. Kolb , M. G. Finn , K. B. Sharpless , Angew. Chem. Int. Ed. 2001, 40, 2004–2021; 10.1002/1521-3773(20010601)40:11<2004::AID-ANIE2004>3.0.CO;2-5 11433435

[19a] H. Nöth , P. Fritz , Z. Anorg. Allg. Chem. 1963, 324, 129–145;

[19b] M. A. M. Alibadi , A. S. Batsanov , G. Bramham , J. P. H. Charmant , M. F. Haddow , L. MacKay , S. M. Mansell , J. E. McGrady , N. C. Norman , A. Roffey , C. A. Russell , Dalton Trans. 2009, 5348–5354; 19565086 10.1039/b822225h

[19c] H. C. Söyleyici , S. Uyanık , R. Sevinçek , E. Fırıncı , B. Bursalı , O. Burgaz , M. Aygün , Y. Şahin , Inorg. Chem. Commun. 2015, 61, 214–216;

[19d] C. J. Carmalt , W. Clegg , A. H. Cowley , F. J. Lawlor , T. B. Marder , N. C. Norman , C. R. Rice , O. J. Sandoval , A. J. Scott , Polyhedron 1997, 16, 2325–2328;

[19e] H. Braunschweig , T. Thiess , T. Kupfer , M. Ernst , Chem. Eur. J. 2020, 26, 2967–2972. 31944442 10.1002/chem.201905356 PMC7078994

[20] J. E. Del Bene , M. Yáñez , I. Alkorta , J. Elguero , J. Chem. Theory Comput. 2009, 5, 2239–2247. 26616610 10.1021/ct900128v

[21a] X. Xie , M. F. Haddow , S. M. Mansell , N. C. Norman , C. A. Russell , Dalton Trans. 2012, 41, 2140–2147; 22187045 10.1039/c2dt11936f

[21b] X. Xie , C. J. Adams , M. A. M. Al-Ibadi , J. E. McGrady , N. C. Norman , C. A. Russell , Chem. Commun. 2013, 49, 10364–10366; 10.1039/c3cc44990d 24079005

[21c] M. Arrowsmith , H. Braunschweig , K. Radacki , T. Thiess , A. Turkin , Chem. Eur. J. 2017, 23, 2179–2184; 27935652 10.1002/chem.201605270

[21d] T. Thiess , S. K. Mellerup , H. Braunschweig , Chem. Eur. J. 2019, 25, 13572–13578. 31429506 10.1002/chem.201903259 PMC6856839

[22a] K. Dehnicke , V. Fernández , Chem. Ber. 1976, 109, 488–492;

[22b] H. Nöth , P. Otto , W. Storch , Chem. Ber. 1985, 118, 3020–3031.

[23a] M. Noltemeyer , F. Pauer , D. Bromm , A. Meller , Acta Crystallogr. Sect. C 1990, 46, 1981–1982;

[23b] A. J. Ashe III , J. W. Kampf , W. Klein , R. Rousseau , Angew. Chem. Int. Ed. Engl. 1993, 32, 1065–1066;

[23c] B. Deobald , J. Hauss , H. Pritzkow , D. Steiner , A. Berndt , W. Siebert , J. Organomet. Chem. 1994, 481, 205–210;

[23d] L. G. Mercier , W. E. Piers , M. Parvez , Angew. Chem. Int. Ed. 2009, 48, 6108–6111; 10.1002/anie.200902803 19598197

[23e] C. Fan , W. E. Piers , M. Parvez , R. McDonald , Organometallics 2010, 29, 5132–5139;

[23f] H. Amarne , C. Baik , R.-Y. Wang , S. Wang , Organometallics 2011, 30, 665–668;

[23g] D. R. Levine , A. Caruso , M. A. Siegler , J. D. Tovar , Chem. Commun. 2012, 48, 6256–6258; 10.1039/c2cc32500d 22596035

[23h] H. Braunschweig , J. Maier , K. Radacki , J. Wahler , Organometallics 2013, 32, 6353–6359;

[23i] A. Iida , S. Saito , T. Sasamori , S. Yamaguchi , Angew. Chem. Int. Ed. 2013, 52, 3760–3764; 10.1002/anie.201210236 23427134

[24] J. H. Barnard , S. Yruegas , K. Huang , C. D. Martin , Chem. Commun. 2016, 52, 9985–9991. 10.1039/c6cc04330e 27345619

[25a] H. Hess , Acta Crystallogr. Sect. B 1969, 25, 2342–2349;

[25b] H. Nöth , M. Schwartz , S. Weber , Chem. Ber. 1985, 118, 4716–4724;

[25c] E. v. Steuber , G. Elter , M. Noltemeyer , H.-G. Schmidt , A. Meller , Organometallics 2000, 19, 5083–5091;

[25d] C. Matthes , U. Klingebiel , S. Deuerlein , H. Ott , D. Stalke , Z. Anorg. Allg. Chem. 2008, 634, 2402–2410.

[26a] B. Iddon , O. Meth-Cohn , E. F. V. Scriven , H. Suschitzky , P. T. Gallagher , Angew. Chem. Int. Ed. Engl. 1979, 18, 900–917;

[26b] G. Smolinsky , E. Wasserman , W. A. Yager , J. Am. Chem. Soc. 1962, 84, 3220–3221.

[27a] S. H. Vosko , L. Wilk , M. Nusair , Can. J. Phys. 1980, 58, 1200–1211;

[27b] C. Lee , W. Yang , R. G. Parr , Phys. Rev. B 1988, 37, 785–789; 10.1103/physrevb.37.785 9944570

[27c] A. D. Becke , J. Chem. Phys. 1993, 98, 5648–5652;

[27d] P. J. Stephens , F. J. Devlin , C. F. Chabalowski , M. J. Frisch , J. Phys. Chem. 1994, 98, 11623–11627;

[27e] R. Krishnan , J. S. Binkley , R. Seeger , J. A. Pople , J. Chem. Phys. 1980, 72, 650–654;

[27f] T. Clark , J. Chandrasekhar , G. W. Spitznagel , P. V. R. Schleyer , J. Comput. Chem. 1983, 4, 294–301;

[27g] S. Grimme , J. Antony , S. Ehrlich , H. Krieg , J. Chem. Phys. 2010, 132, 154104; 20423165 10.1063/1.3382344

[27h] A. V. Marenich , C. J. Cramer , D. G. Truhlar , J. Phys. Chem. B 2009, 113, 6378–6396. 19366259 10.1021/jp810292n

[28] B. O. Roos , in Advances in Chemical Physics, Wiley, Hoboken, New Jersey, 1987, pp. 399–445.

[29a] C. Angeli , R. Cimiraglia , S. Evangelisti , T. Leininger , J.-P. Malrieu , J. Chem. Phys. 2001, 114, 10252–10264;

[29b] C. Angeli , R. Cimiraglia , J.-P. Malrieu , Chem. Phys. Lett. 2001, 350, 297–305;

[29c] C. Angeli , R. Cimiraglia , J.-P. Malrieu , J. Chem. Phys. 2002, 117, 9138–9153.

[30] T. H. Dunning , J. Chem. Phys. 1989, 90, 1007–1023.

[31] CCDC 1984944, 1984945, 1984946, 1984947, and 1984948 contain the supplementary crystallographic data for this paper. These data can be obtained free of charge from the Cambridge Crystallographic Data Centre.

