# Supplementary material for: Diborane(4) Azides: Surprisingly Stable Sources of Transient Iminoboranes
Source: Angew Chem Int Ed Engl. 2020 May 28;59(36):15480–6. doi: 10.1002/anie.202003050 (PMC7497274; doi:10.1002/anie.202003050)
Supplement: Supplementary file 1 — Supplementary [file ANIE-59-15480-s001.pdf]

Angewandte Chemie
Eine Zeitschrift der Gesellschaft Deutscher Chemiker

Supporting Information

Diborane(4) Azides: Surprisingly Stable Sources of Transient Iminoboranes

*Torsten Thiess, Guillaume Bélanger-Chabot[+], Felipe Fantuzzi[+], Maximilian Michel, Moritz Ernst, Bernd Engels, and Holger Braunschweig**

anie_202003050_sm_miscellaneous_information.pdf

Table of Contents

Methods and Materials 1
Synthesis and Characterization 2
X-ray Crystallographic Details 22
Computational Details 33
Conformational Analysis of 1 (B3LYP/6-31+G*) 36
Geometrical Parameters 37
Hessian Calculations and Thermochemical Analysis 39
Molecular Orbital Diagrams and Spin Densities 46
Nucleus-Independent Chemical Shift 48
CASSCF and NEVPT2 Results 49
Reaction Mechanism 51
Cartesian Coordinates 54
References 81

Methods and Materials

All manipulations were performed either under an atmosphere of dry argon or *in vacuo* using standard Schlenk line or glovebox techniques. Deuterated benzene was dried over molecular sieves and degassed by three freeze-pump-thaw cycles prior to use. All other solvents were distilled and degassed from appropriate drying agents. Solvents were stored under argon over activated 4 Å molecular sieves. NMR spectra were acquired on a Bruker Avance 500 NMR spectrometer (^{1}H: 500.1 MHz, ^{11}B:160.5 MHz, ^{13}C{^{1}H}: 125.8 MHz) or on a Bruker Avance 400 NMR spectrometer (^{1}H: 400.1 MHz, ^{11}B: 128.4 MHz, ^{13}C 100.613 MHz, ^{14}N 28.915 MHz). ^{1}H and ^{13}C signals were assigned based on data from DEPT-135, HSQC, HMBC and COSY experiments. Chemical shifts (δ) are given in ppm and internally referenced to the solvent signal (^{13}C NMR) or the residual protic signal (^{1}H NMR) of the solvent. ^{11}B NMR and ^{14}N NMR spectra were externally referenced to [BF_3·OEt_2] and $MeNO_2$, respectively. Photolysis reactions were performed under a mercury vapor lamp (current: 19 A, voltage: 26 V). IR spectra were acquired in the argon atmosphere of a glovebox on a Bruker Alpha spectrometer equipped with an ATR module.

1,2-Dichloro-1,2-bis(dimethylamido)diborane(4) (**$B_2(NMe_2)_2Cl_2$**),[1] 2,3-dibromo-1,4-dimesityl-1,4-diaza-2,3-diborinine (**MDBBr**), 2,3-dichloro-1,4-dimesityl-1,4-diaza-2,3-diborinine (**MDBCl**), 2,3-dibromo-1,4-bis(2,5-dimethylphenyl)-1,4-diaza-2,3-diborinine·Et_2O (**XDBBr·Et_2O**) and 2,3-dichloro-1,4-bis(4-methylphenyl)-1,4-diaza-2,3-diborinine·(**pTolDBCl**) were synthesized as described in the literature.[2] Trimethylsilyl azide ($TMSN_3$) was purchased from TCI and used without further purification.

Synthesis and Characterization

1,2-Diazido-1,2-bis(dimethylamido)diborane(4) (1)

$B_2(NMe_2)_2Cl_2$ (163 mg; 0.903 mmol) and $TMSN_3$ (204 mg; 1.77 mmol) solutions in dichloromethane were placed in a glass reactor equipped with a Teflon valve. The reaction mixture was stirred for several days at room temperature. The product is volatile enough that most will evaporate if subjected to a dynamic vacuum for a significant amount of time. The crude product obtained by the removal of the solvent *in vacuo* at 0 °C is of acceptable purity. To remove most of the traces of leftover starting materials and impurities thereof, the contents of the flask were separated by two fractional condensations where the product was recovered in a 0 °C trap. **1** was obtained as a faint-yellow liquid (80 mg, 46 mol% yield). The product travels very slowly under static vacuum and operations have to be conducted under dynamic vacuum. The compound can also be observed by treating a dichloromethane solution of $B_2(NMe_2)_2Cl_2$ with two equivalents of $[PPh_4]N_3$.

^{1}H NMR (400 MHz, C_6D_6): δ = 2.41 (s, 6H, NCH_3), 2.39 (s, 6H, NCH_3). **^{11}B NMR** (128 MHz, C_6D_6): δ = 34.8 (s, br) ppm. **^{13}C{^{1}H} NMR** (100 MHz, C_6D_6): 40.67 (s, NCH$_3$), 36.71 (s, NCH$_3$). **HRMS-ASAP**: $[M\text{-}H]^+$ 195.1440 (calculated: 195.1444).

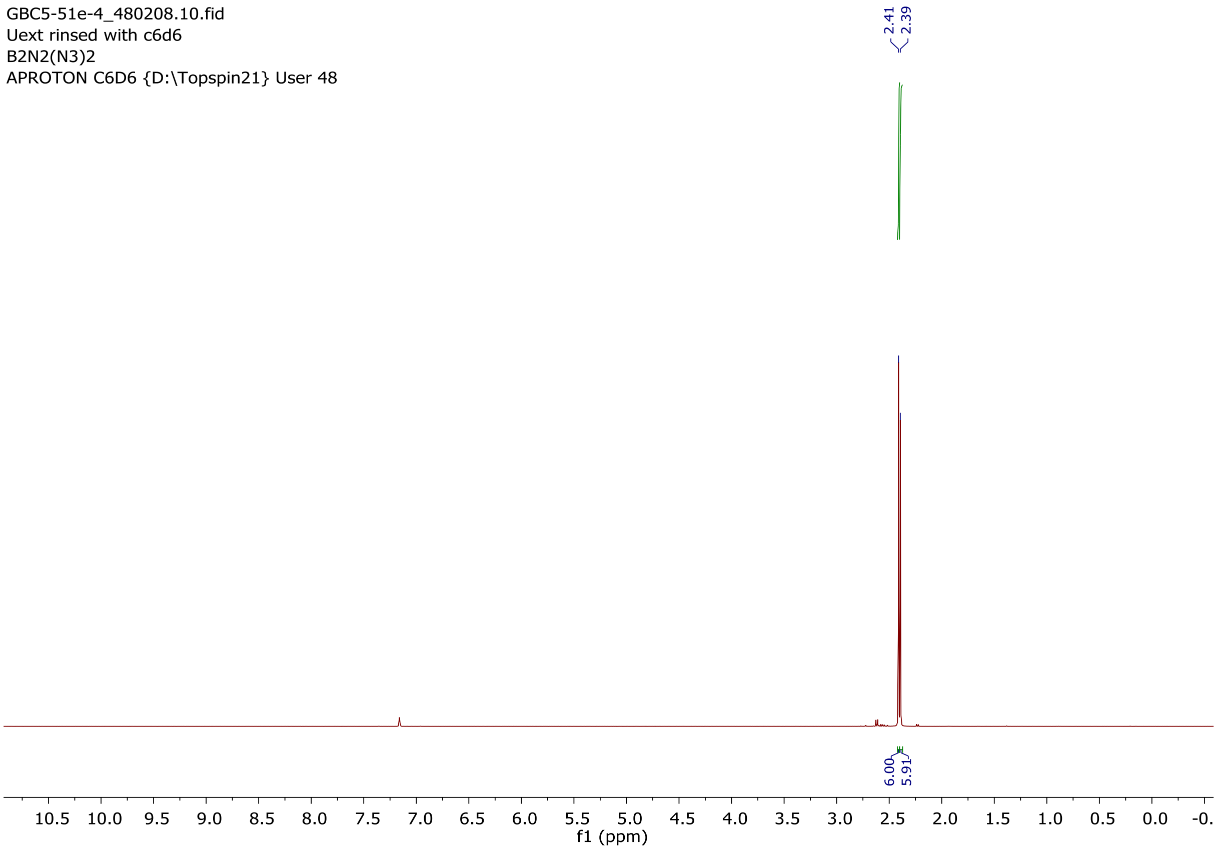

Figure S1: ^{1}H NMR spectrum of **1** in C_6D_6.

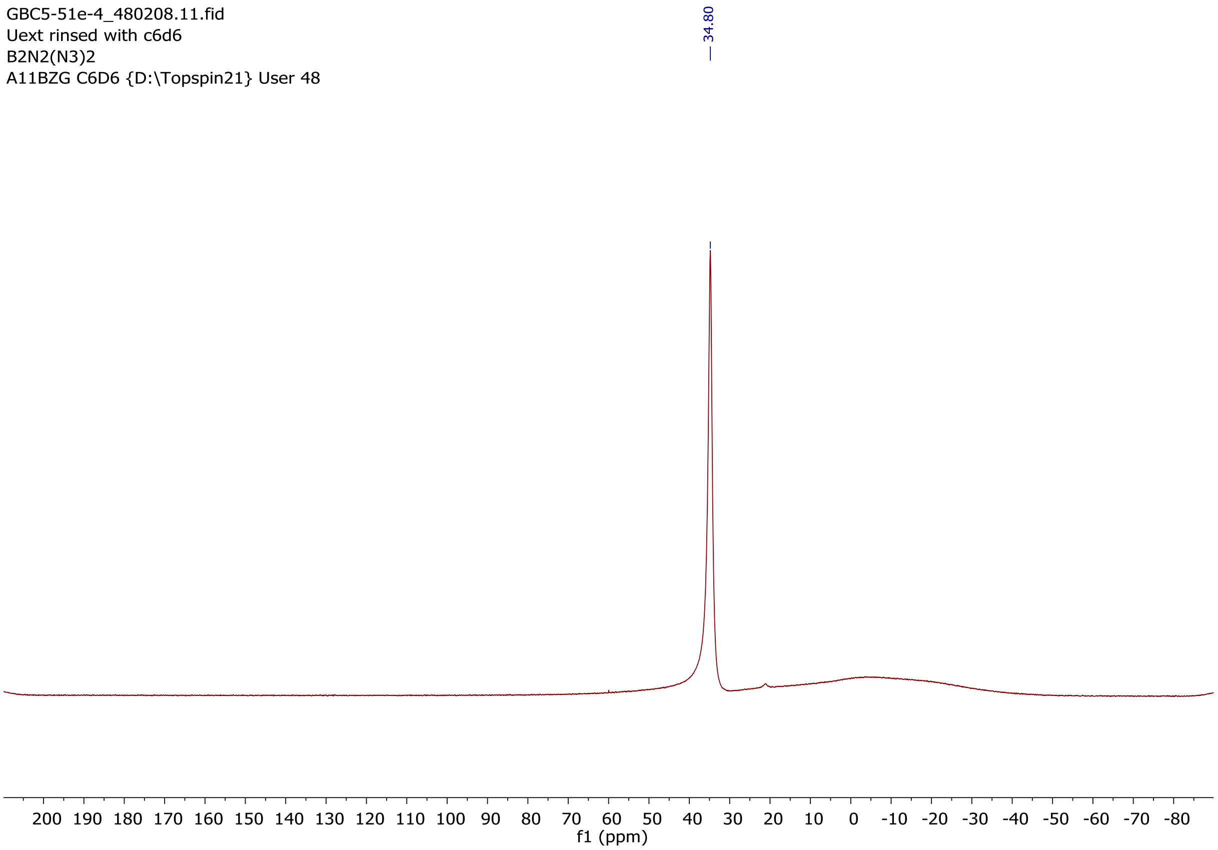

Figure S2: ^{11}B NMR spectrum of **1** in C_6D_6.

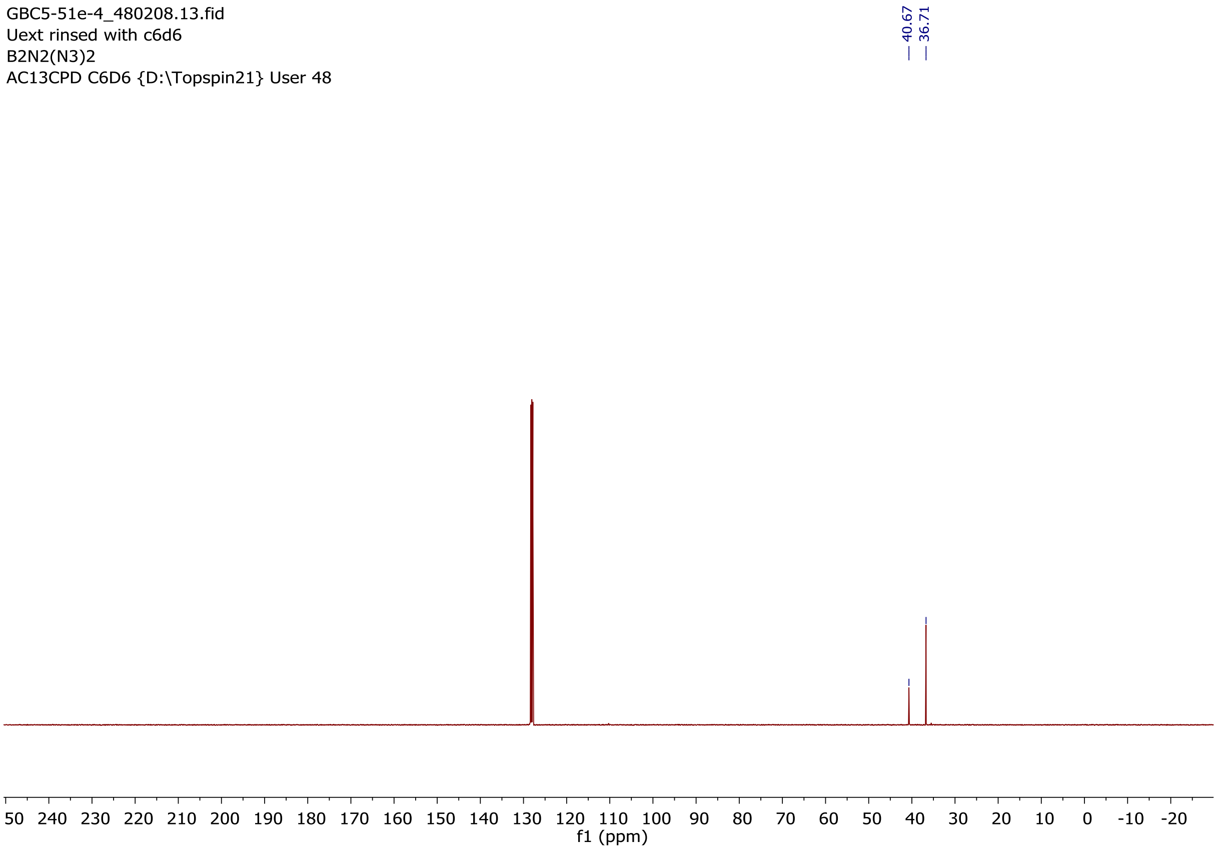

Figure S3: $^{13}C\{^{1}H\}$ NMR spectrum of **1** in C_6D_6.

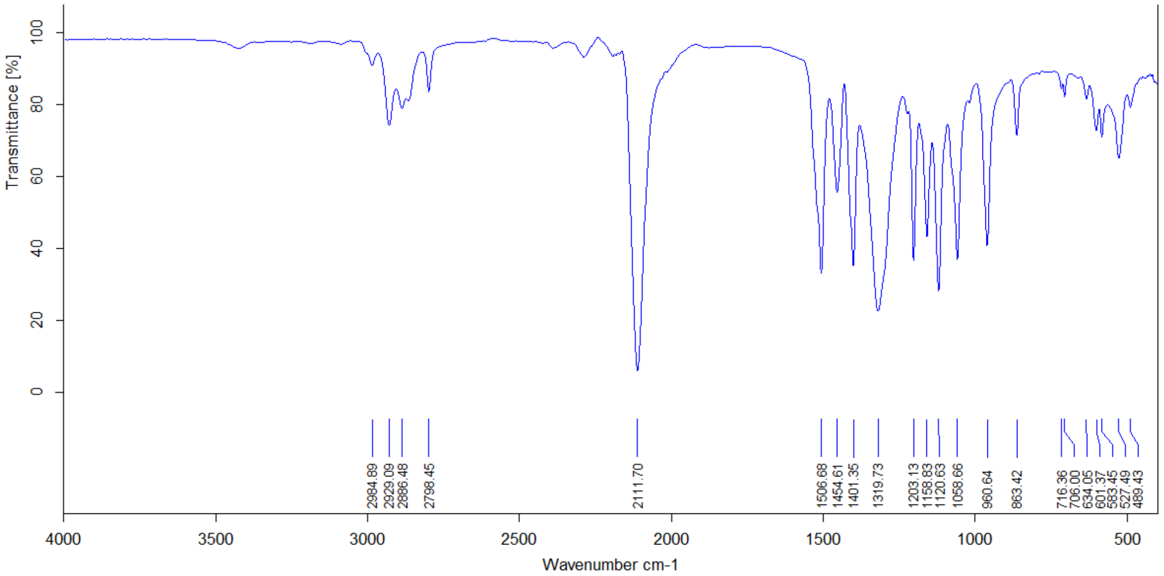

Figure S4: ATR-IR spectrum of **1**.

2,3-Diazido-1,4-dimesityl-1,4-diaza-2,3-diborinine (MDBN3) (2a)

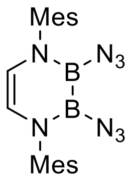

a) **MDBBr** (1.00 g, 2.10 mmol) was dissolved in benzene (25 mL) along with an excess of $TMSN_3$ and the mixture was stirred at ambient temperature for 16 hours. The solvent was frozen in liquid nitrogen and sublimed *in vacuo* while the vessel was left to warm to room temperature. **2a** was isolated as a colorless powder (790 mg, 1.98 mmol, 95%).

b) **MDBCl** (500 mg, 1.30 mmol) was dissolved in benzene (15 mL), treated with an excess of $TMSN_3$ and stirred at 60 °C for 16 hours. The solvent was frozen in liquid nitrogen and sublimed *in vacuo* while the vessel was left to warm to room temperature. **2a** was isolated as a colorless powder (471 mg, 1.05 mmol, 81%). The product was spectroscopically identical to that obtained in a).

^{1}H NMR (500 MHz, C_6D_6): δ = 6.78 (s, 4H, *meta*-C*H*), 5.37 (s, 2H, C*H*=CH), 2.13 (s, 6H, *para*-C*H*3), 2.06 (s, 12H, *ortho*-C*H*3) ppm. **^{11}B NMR** (160 MHz, C_6D_6): δ = 34.0 (s, br) ppm. **^{13}C{^{1}H} NMR** (125 MHz, C_6D_6): δ = 141.45 (s, *ipso*-*C*q), 137.04 (s, *para*-*C*q), 133.69 (s, *ortho*-*C*q), 129.32 (s, *meta*-*C*H), 118.75 (s, *C*H), 20.96 (s, *para*-*C*H3), 17.96 (s, *ortho*-*C*H3) ppm. **HRMS-ASAP:** $[M-H]^+$ 399.2383 (calculated: 399.238827).

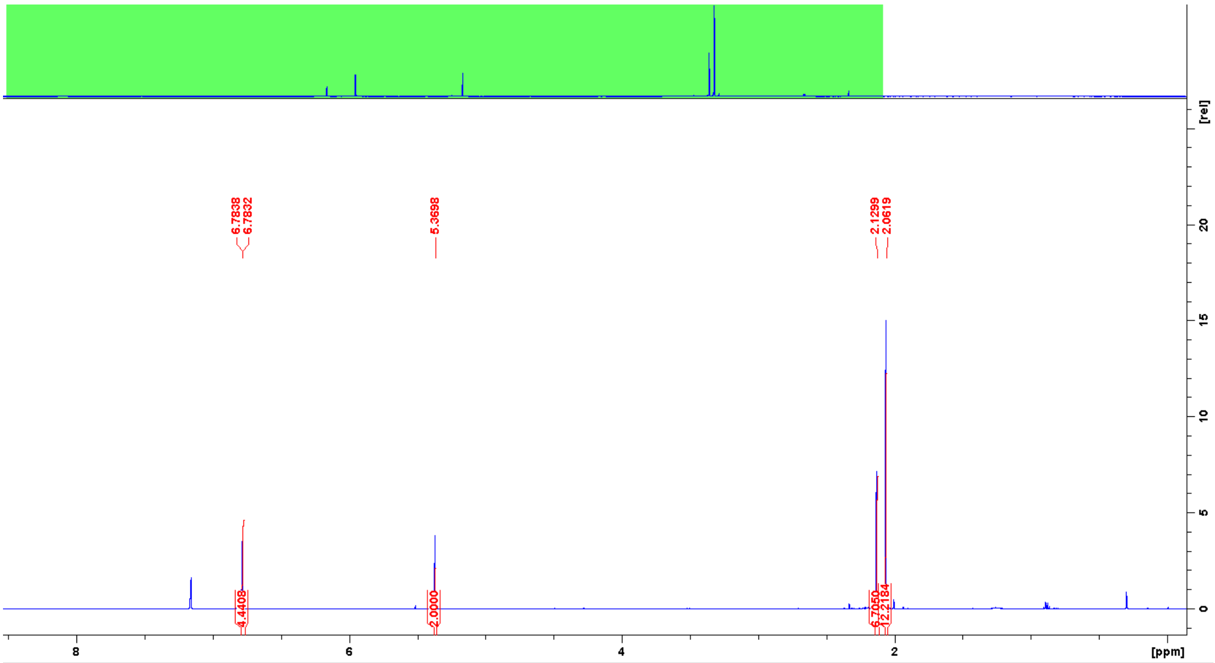

Figure S5: ^{1}H NMR spectrum of **2a** in C_6D_6.

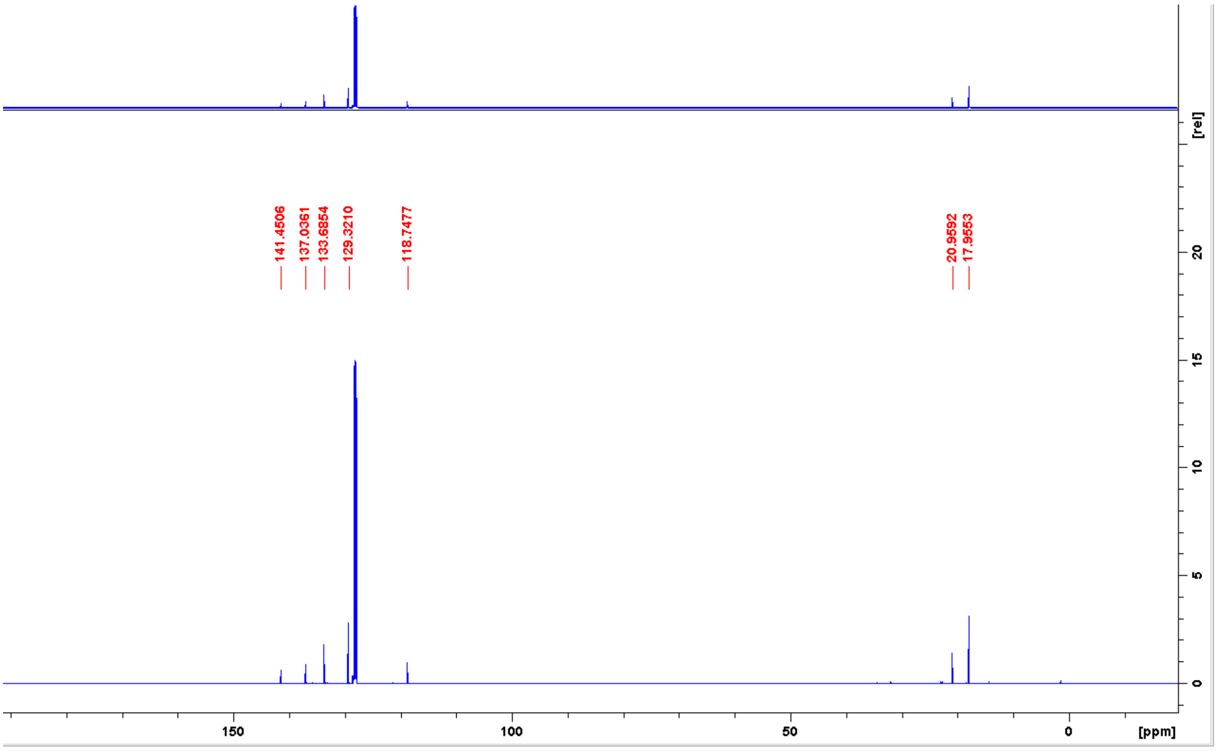

Figure S6: ^{13}C{^{1}H} NMR spectrum of **2a** in C_6D_6.

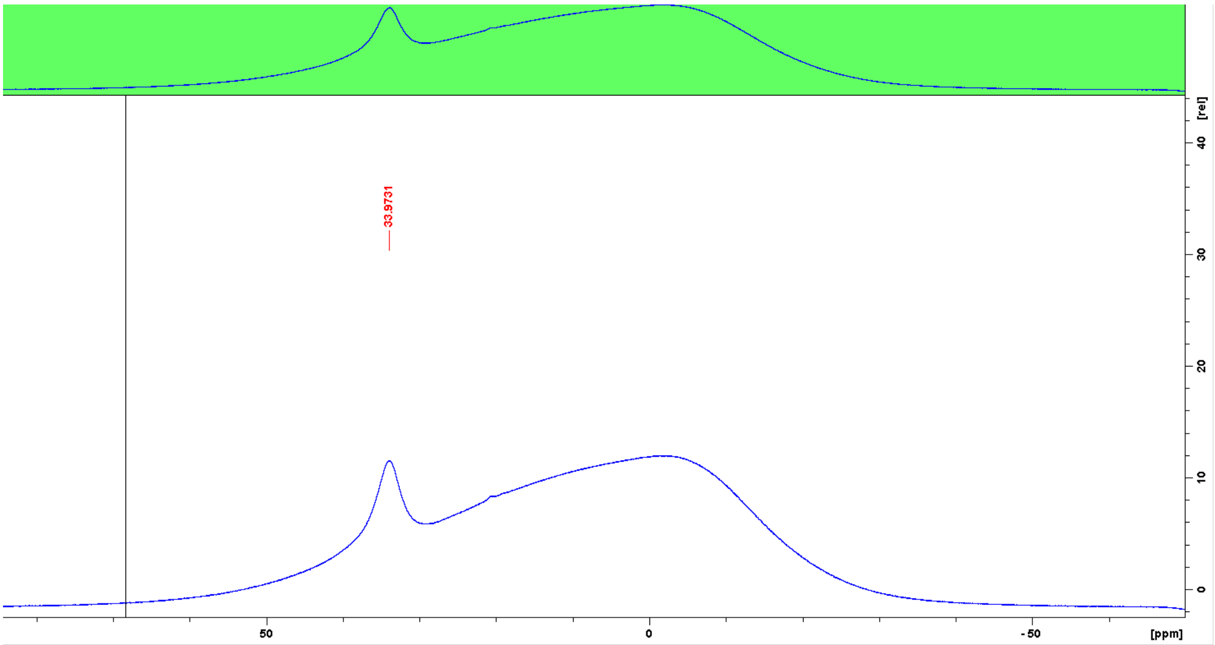

Figure S7: ^{11}B NMR spectrum of **2a** in C_6D_6.

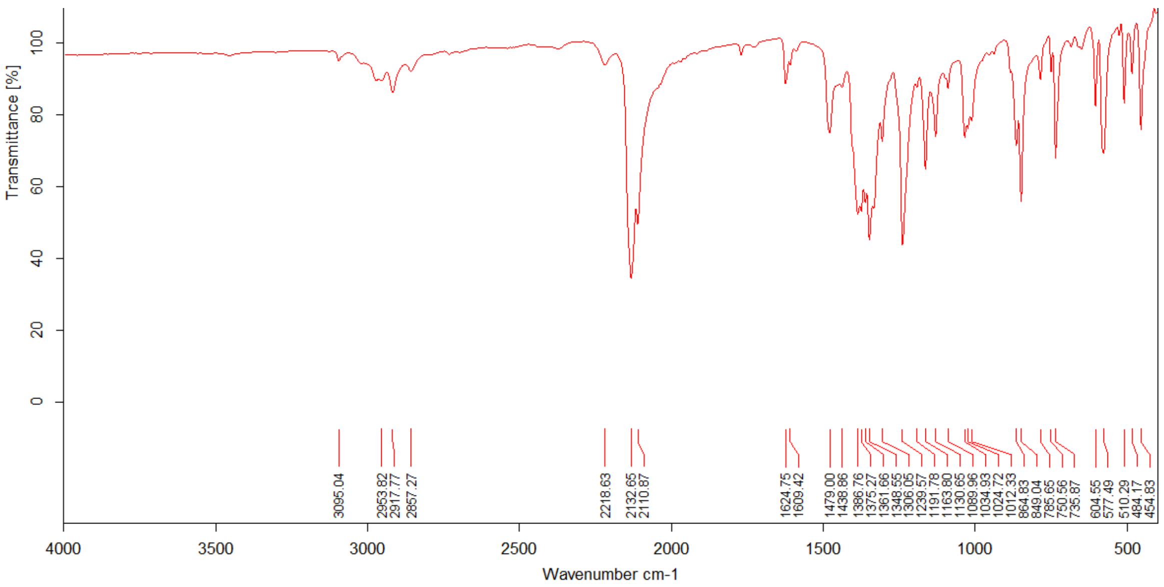

Figure S8: ATR-IR spectrum of **2a**.

2,3-Diazido-1,4-bis(2,6-dimethylphenyl)-1,4-diaza-2,3-diborinine (XDBN3) (2b)

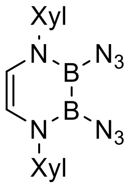

XDBBr·Et_2O (218.0 mg, 0.422 mmol) was dissolved in benzene (10 mL), treated with an excess of $TMSN_3$ and stirred at ambient temperature for one hour followed by reflux for five minutes. The solvent was frozen in liquid nitrogen and sublimed *in vacuo* while the vessel was left to warm to room temperature. **2b** was isolated as a colorless powder (146.0 mg, 394.6 µmol, 94%).

^{1}H NMR (500 MHz, C_6D_6): δ = 7.02–7.00 (m, 2H, aryl-C*H*), 6.97–6.95 (m, 4H, aryl-C*H*), 5.28 (s, 2H, C*H*=CH), 2.05 (s, 6H, *ortho*-C*H*$_3$) ppm. **^{11}B NMR** (160 MHz, C_6D_6): δ = 33.8 (s, br) ppm. **^{13}C{^{1}H} NMR** (125 MHz, C_6D_6): δ = 143.91 (s, *ipso*-*C*$_q$), 134.07 (s, *ortho*-*C*$_q$), 127.73 (s, aryl-*C*H), 128.62 (s, aryl-*C*H), 118.39 (s, C*H*=CH), 17.99 (s, *ortho*-*C*H$_3$) ppm. **HRMS-ASAP**: $[M-H]^+$ 371.2070 (calculated: 371.2075).

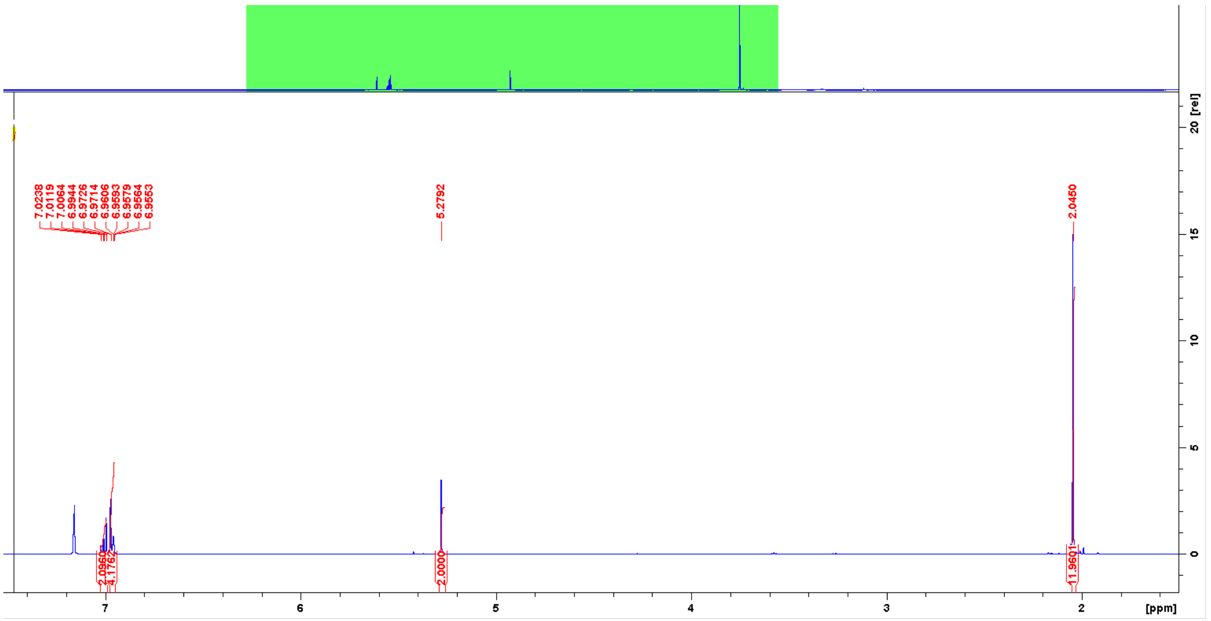

Figure S9: ^{1}H NMR spectrum of **2b** in C_6D_6.

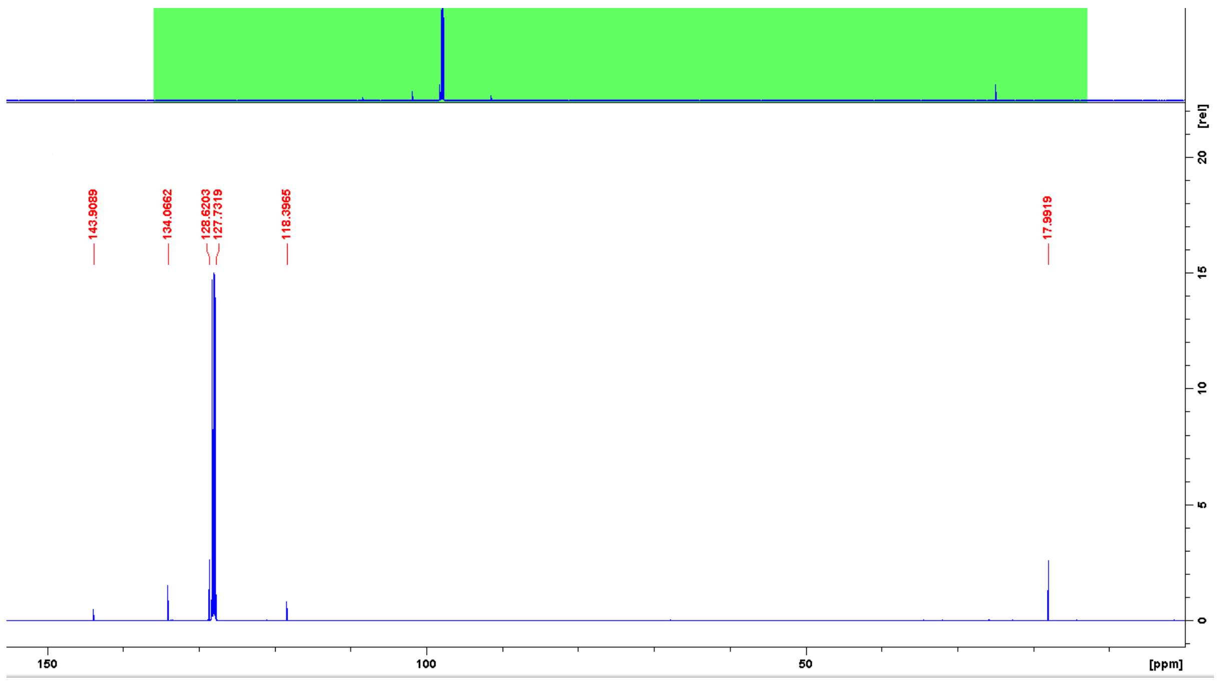

Figure S10: $^{13}C\{^{1}H\}$ NMR spectrum of **2b** in C_6D_6.

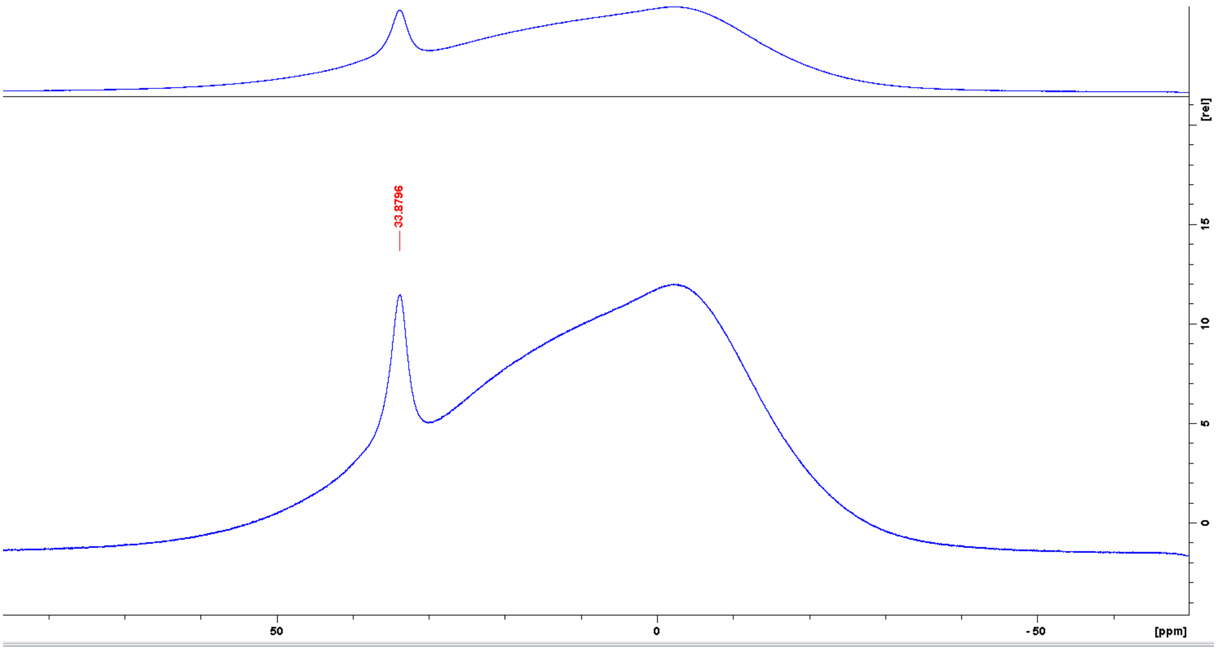

Figure S11: ^{11}B NMR spectrum of **2b** in C_6D_6.

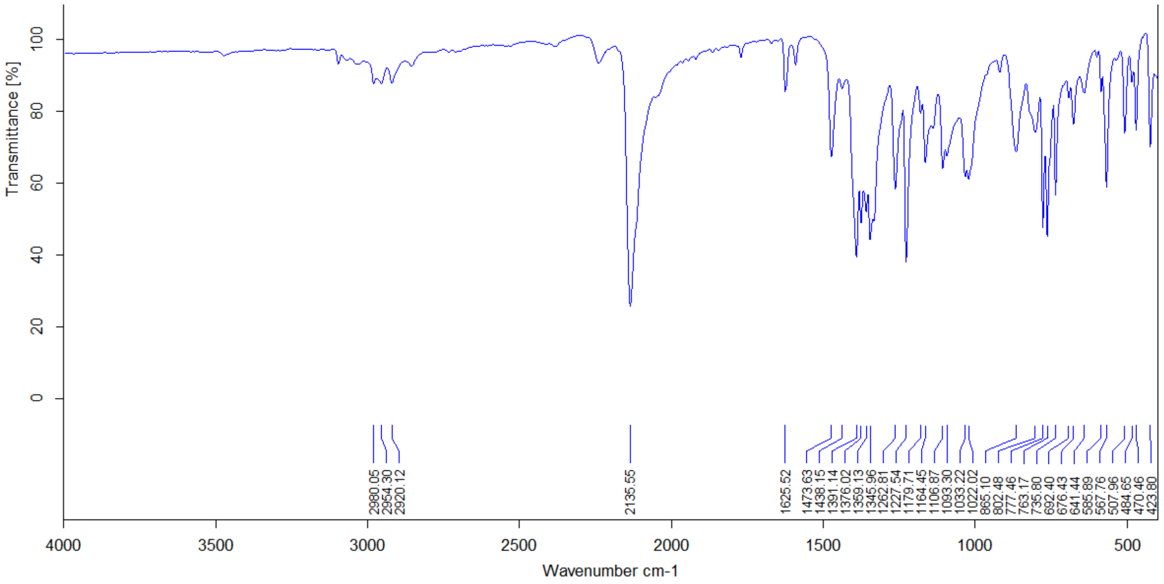

Figure S12: ATR-IR spectrum of **2b**.

2,3-Diazido-1,4-bis(4-methylphenyl)-1,4-diaza-2,3-diborinine (pTolDBN3) (2c)

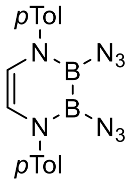

pTolDBCl (100 mg, 304 µmol) was dissolved in 10 mL benzene, treated with an excess of $TMSN_3$ and the mixture was stirred at ambient temperature for 16 hours. The solvent was frozen in liquid nitrogen and sublimed *in vacuo* while the vessel was left to warm to room temperature. **2c** was isolated as a colorless powder (90.3 mg, 364 µmol, 87%).

^{1}H NMR (500 MHz, C_6D_6): δ = 6.99–6.93 (m, 8H, aryl-C*H*), 5.75 (s, 2H, C*H*=CH), 2.09 (s, 6H, *para*-C*H*$_3$) ppm. **^{11}B NMR** (160 MHz, C_6D_6): δ = 33.5 (s, br) ppm. **^{13}C{^{1}H} NMR** (125 MHz, C_6D_6): δ = 143.25 (s, *ipso*-*C*$_q$), 136.63 (s, *para*-*C*$_q$), 129.87 (s, *meta*-*C*H), 126.17 (s, *para*-*C*H), 118. (s, *C*H=CH), 20.88 (s, *para*-*C*H$_3$) ppm. **HRMS-ASAP**: [M-H]$^+$ 343.1757 (calculated: 343.1762).

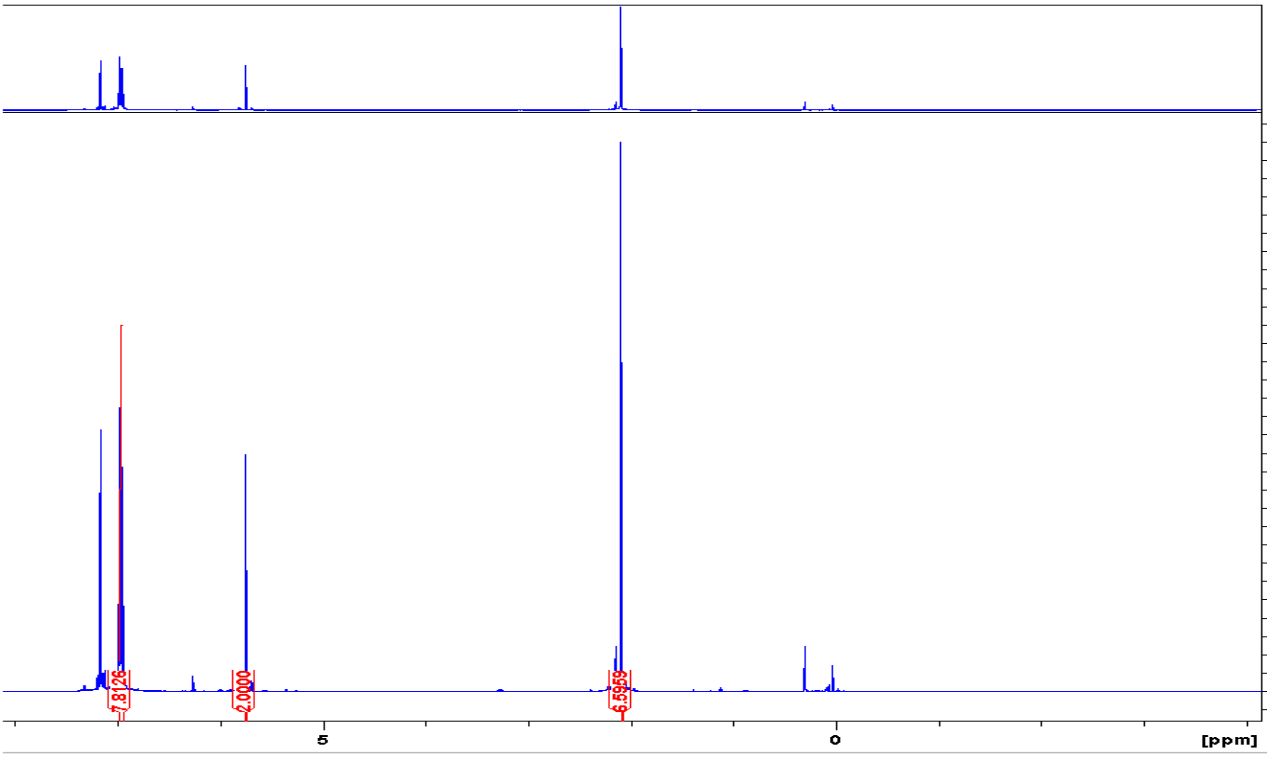

Figure S13: ^{1}H NMR spectrum of **2c** in C_6D_6.

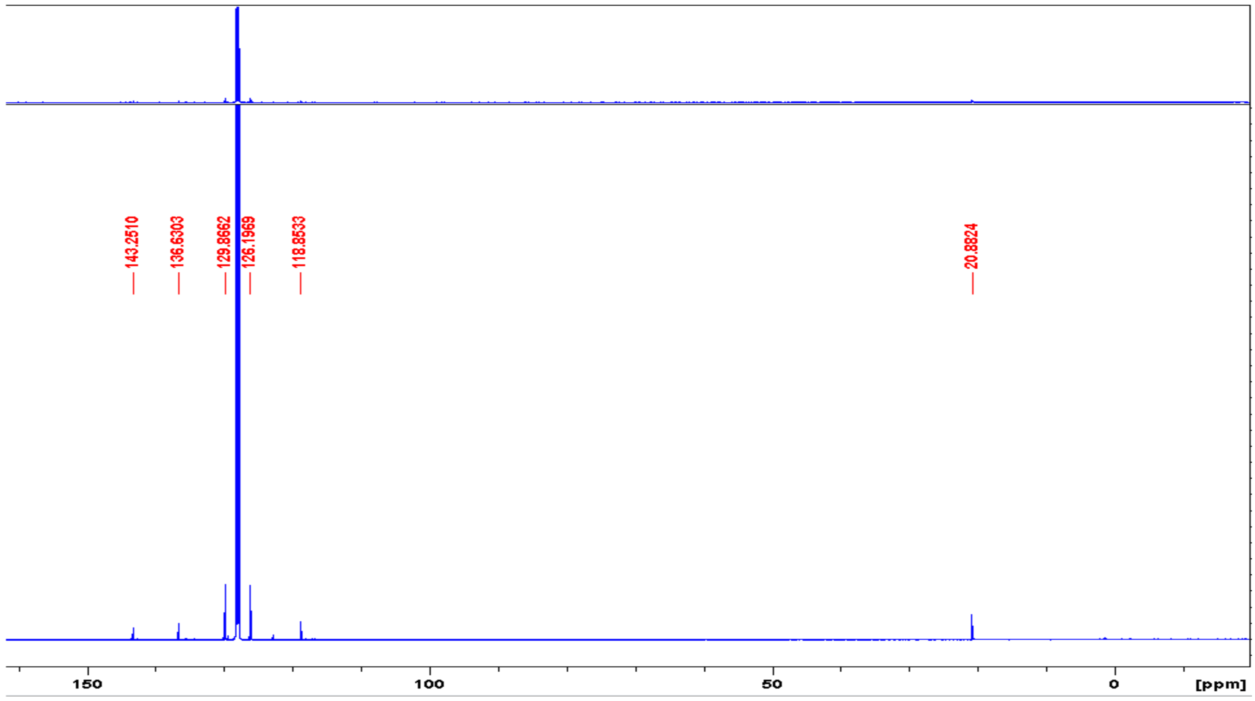

Figure S14: $^{13}C\{^{1}H\}$ NMR spectrum of **2c** in C_6D_6.

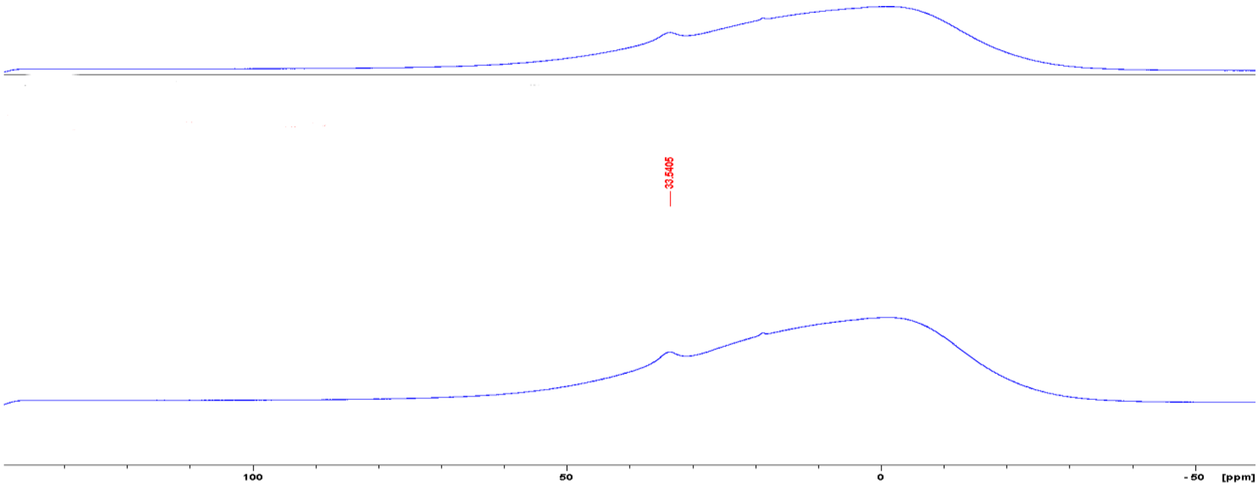

Figure S15: ^{11}B NMR spectrum of **2c** in C_6D_6.

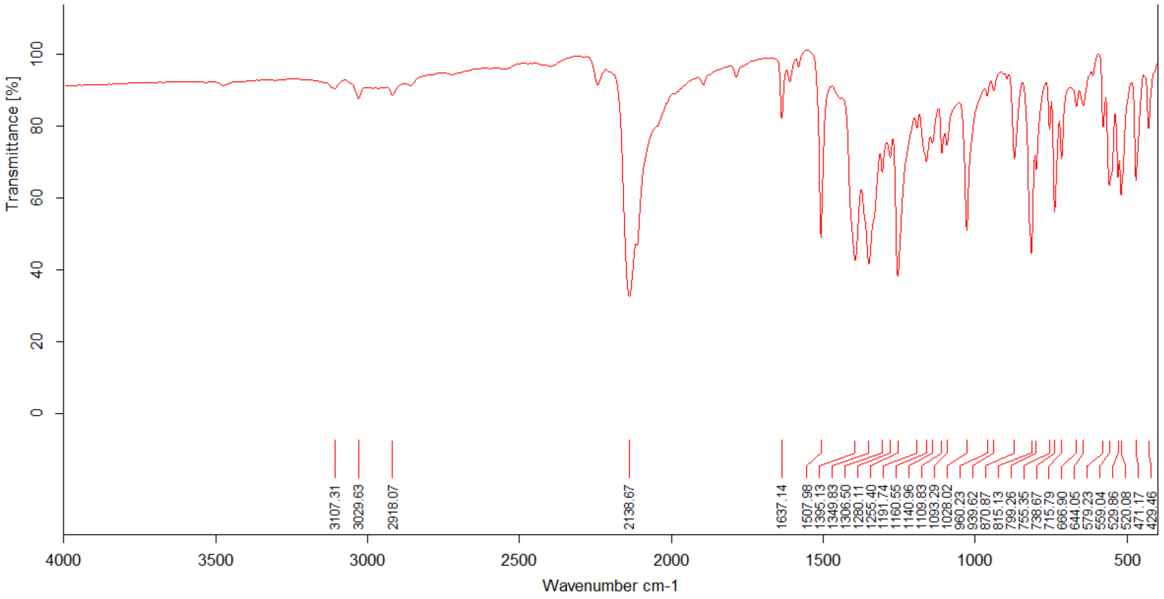

Figure S16: ATR-IR spectrum of **2c**.

1,8-Diazido-2,5,9,12-tetramesityl-2,5,9,12-tetrahydro-1*H*,8*H*-[1,3,2,4]diazadiboreto[2,1-*b*:4,3-*b'*]bis([1,3,5,2,4]triazadiborepine) (MDBN3dT) (3a)

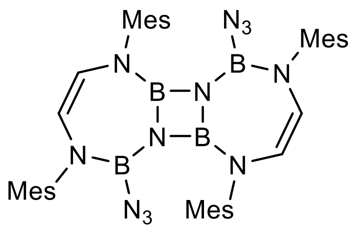

1,8-diazido-2,5,9,12-tetramesityl-2,5,9,12-tetrahydro-1*H*,8*H*-[1,3,2,4]diazadiboreto[2,1-*b*:4,3-*b'*]bis([1,3,5,2,4]triazadiborepine)

a) **2a** (23 mg, 57.8 µmol) was placed in a narrow Schlenk flask. The atmosphere was removed *in vacuo* and the flask sealed under vacuum. The flask was heated with a heatgun, whereupon the melting of the substrate could be observed, followed by what appeared to be a deflagration that did not damage the vessel. The resulting brown residue was extracted with pentane and the extract stored at –30 °C to yield colorless crystals of **3a** (16 mg, 21.62 µmol, 75%).

b) **2a** (80.6 mg, 239 µmol) was placed in a narrow Schlenk flask. The atmosphere was removed *in vacuo* and the flask sealed under vacuum. The flask was heated to 150 °C in an oil bath wereupon melting and the evolution of gas was observed. The brown residue was extracted with pentane and the extract stored at –30 °C to yield colorless crystals of **3a** (62.3 mg, 84.2 µmol, 84%).

c) **2a** (108 mg, 270 µmol) was dissolved in mesitylene (0.5 mL) and heated to 150 °C for one hour until a yellow solution was obtained. The solvent was removed *in vacuo* and the residue extracted with pentane and the extract stored at –30 °C for 16 hours to yield colorless crystals of **3a** (78.3 mg, 106 µmol, 78%).

^{1}H NMR (500 MHz, C_6D_6): δ = 6.80 (m, 4H, aryl-C*H*), 6.70 (m, 4H, aryl-C*H*), 4.55 (d, 2H, $^{3}J_{HH}$ = 8.53 Hz, C=C*H*), 4.35 (d, 2H, $^{3}J_{HH}$ = 8.53 Hz, C=C*H*), 2.44 (s, 12H, *ortho*-CH_3), 2.25 (s, 12H, *ortho*-CH_3), 2.10 (s, 6H, *para*-CH_3), 2.04 (s, 6H, *para*-CH_3) ppm. **^{11}B NMR** (160 MHz, C_6D_6): δ = 29.1 (s, N*B*N), 22.3 (s, *B*N_3) ppm. **^{13}C{^{1}H} NMR** (125 MHz, C_6D_6): δ = 141.30 (s, *ipso*-*C*q), 140.90 (s, *ipso*-*C*q), 136.85 (s, *para*-*C*q), 136.61 (s, *para*-*C*q), 136.21 (s, *ortho*-*C*q), 136.00 (s, *ortho*-*C*q), 129.63 (s, *meta*-*C*H), 129.05 (s, *meta*-*C*H), 117.25 (s, C=*C*H), 116.41 (s,

C=CH), 20.93 (s, *para*-CH_3), 20.87 (s, *para*-CH_3), 18.38 (s, *ortho*-CH_3), 17.93 (s, *ortho*-CH_3) ppm. **HRMS-ASAP**: $[M-H]^+$ 741.4570 (calculated: 741.4575).

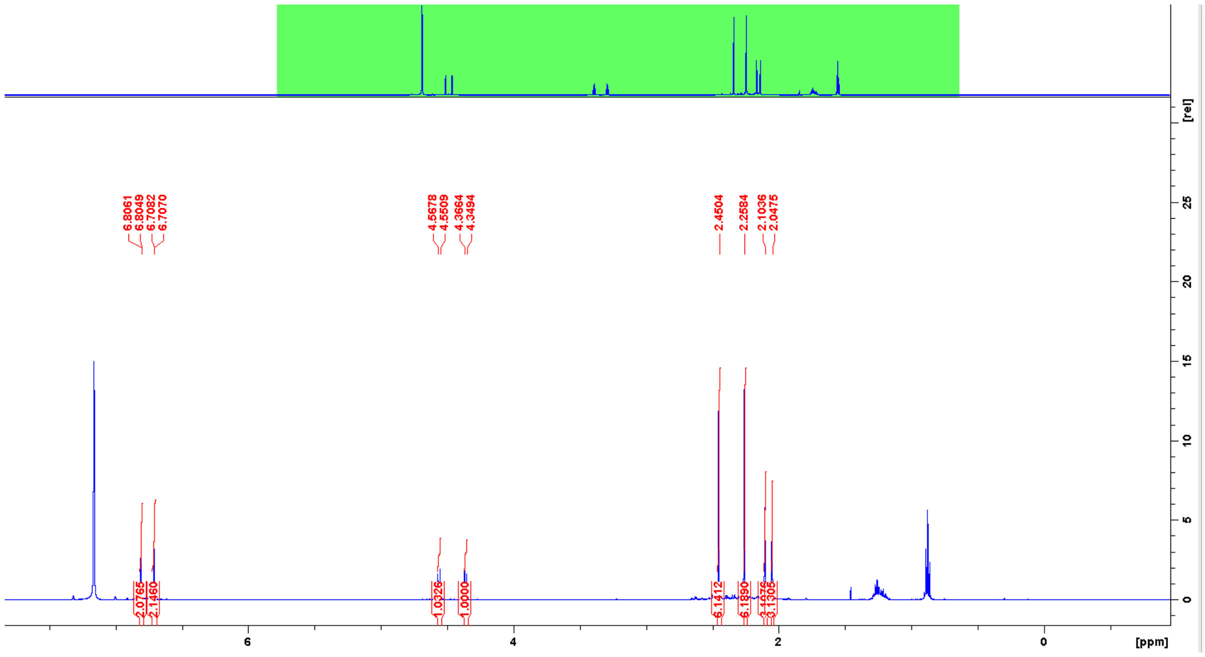

Figure S17: ^1H NMR spectrum of **3a** in C_6D_6. Signals between 1 and 2 ppm are due to residual pentane.

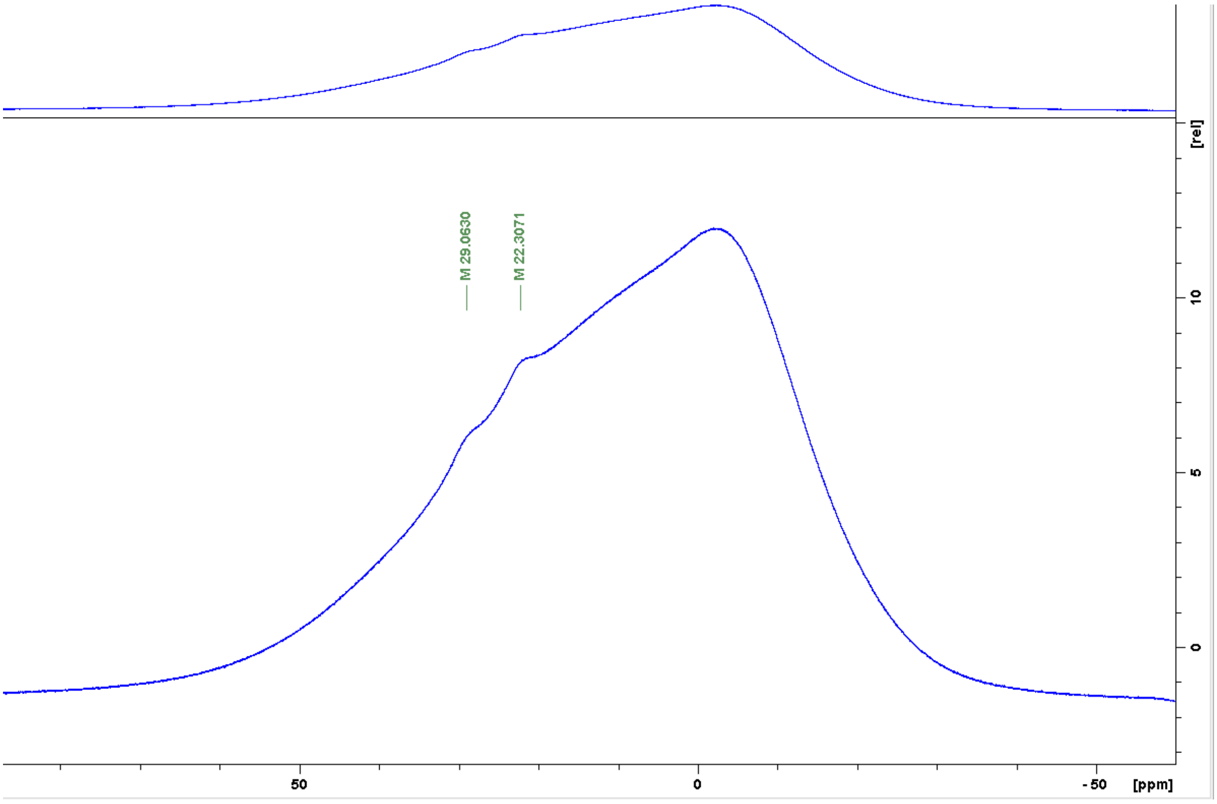

Figure S18: ^{11}B NMR spectrum of **3a** in C_6D_6.

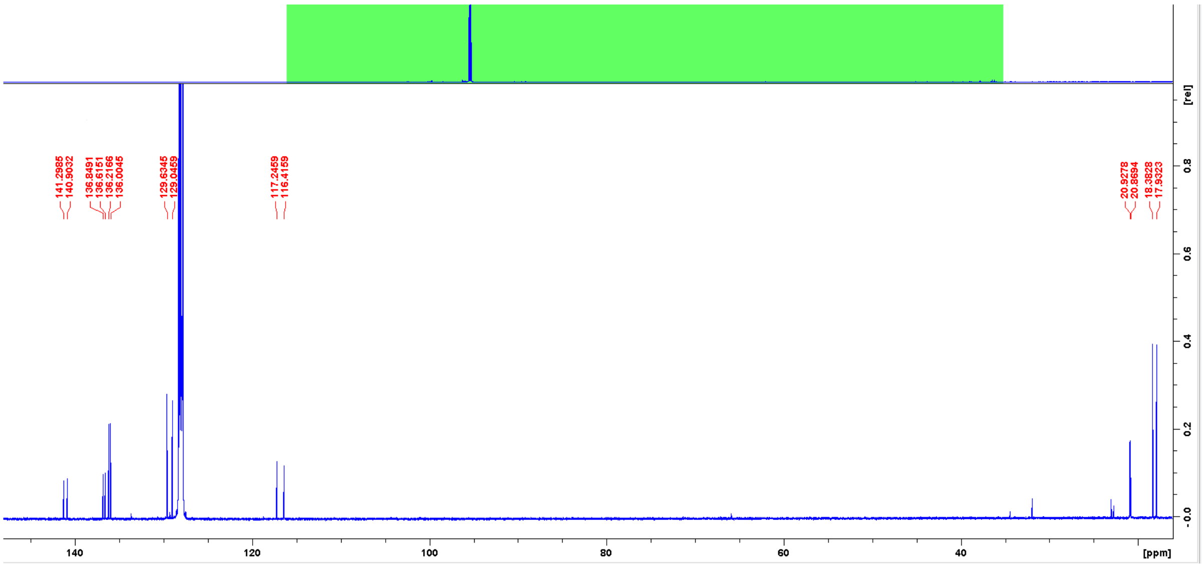

Figure S19: ^{13}C NMR spectrum of **3a** in C_6D_6.

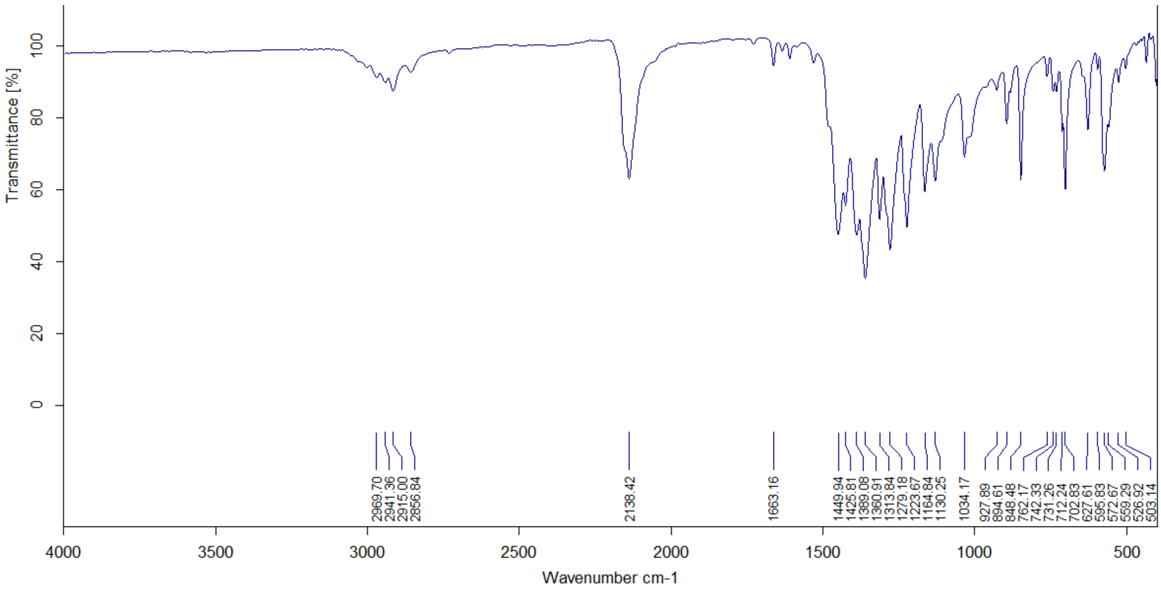

Figure S20: ATR-IR spectrum of **3a**.

1,8-Diazido-2,5,9,12-tetrakis(2,6-dimethylphenyl)-2,5,9,12-tetrahydro-1*H*,8*H*-[1,3,2,4]diazadiboreto[2,1-*b*:4,3-*b'*]bis([1,3,5,2,4]triazadiborepine) (XDBN3dT) (3b)

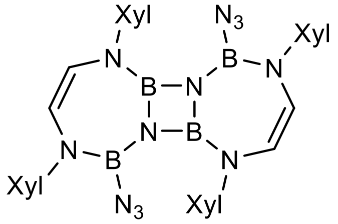

1,8-diazido-2,5,9,12-tetrakis(2,6-dimethylphenyl)-2,5,9,12-tetrahydro-1*H*,8*H*-[1,3,2,4]diazadiboreto[2,1-*b*:4,3-*b'*]bis([1,3,5,2,4]triazadiborepine)

2b (29 mg, 78.4 µmol) was placed in a narrow Schlenk flask. The atmosphere was removed *in vacuo* and the flask sealed under vacuum. The flask was heated with a heatgun, whereupon the melting of the substrate could be observed, followed by an apparent deflagration that did not damage the vessel. The brown residue was extracted with pentane and the extract was stored at –30 °C to yield colorless crystals of **3b** (11.3 mg, 16.5 µmol, 42%).

^{1}H NMR (500.13 MHz, C_6D_6): δ (ppm) = 6.99–6.88 (m, 12 H, aryl-C*H*), 4.48 (d, 1H, $^{3}J_{HH}$ =8.43 Hz, HC=C*H*), 4.29 (d, 1H, $^{3}J_{HH}$ = 8.43 Hz, H*C*=CH), 2.44 (s, 12 H, *ortho*-C*H*$_3$), 2.23 (s, 12 H, *ortho*-C*H*$_3$). **^{11}B NMR** (160.46 MHz, C_6D_6): δ (ppm) = 29.0 (brs, N*B*N), 22.1 (brs, *B*N$_3$). **^{13}C{^{1}H} NMR** (125.76 MHz, C_6D_6): δ (ppm) = 143.63 (s, *ipso*-*C*$_q$), 143.24 (s, *ipso*-*C*$_q$), 136.48 (s, *para*-*C*H), 136.44 (s, *para*-*C*H), 128.94 (s, *meta*-*C*H), 128.40 (s, *meta*-*C*H), 127.69 (s, *ortho*-

*C*q), 127.53 (s, *ortho*-*C*q), 117.03 (s, HC=*C*H), 116.24 (s, H*C*=CH), 18.41 (s, *ortho*-*C*H3), 17.95 (s, *ortho*-*C*H3). **HRMS-ASAP**: [M-H]$^+$ 685.3944 (calculated: 685.3949).

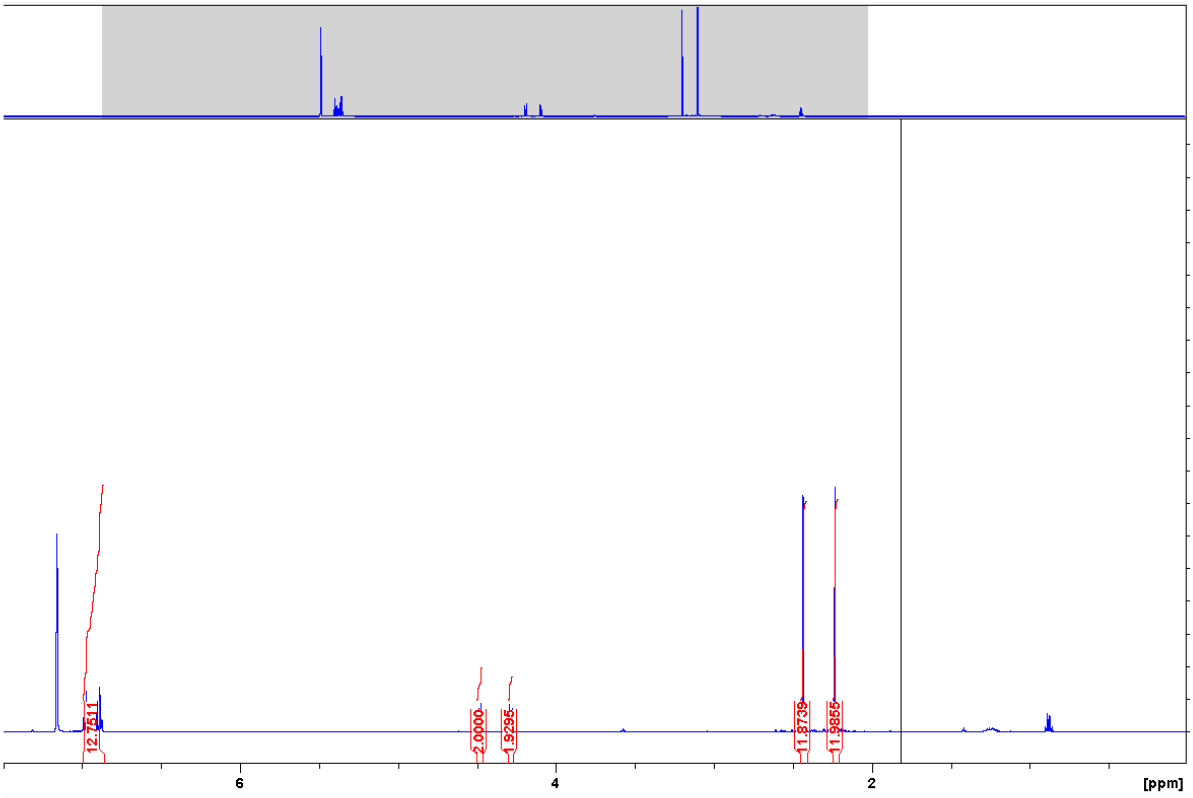

Figure S21: ^{1}H NMR spectrum of **3b** in C_6D_6.

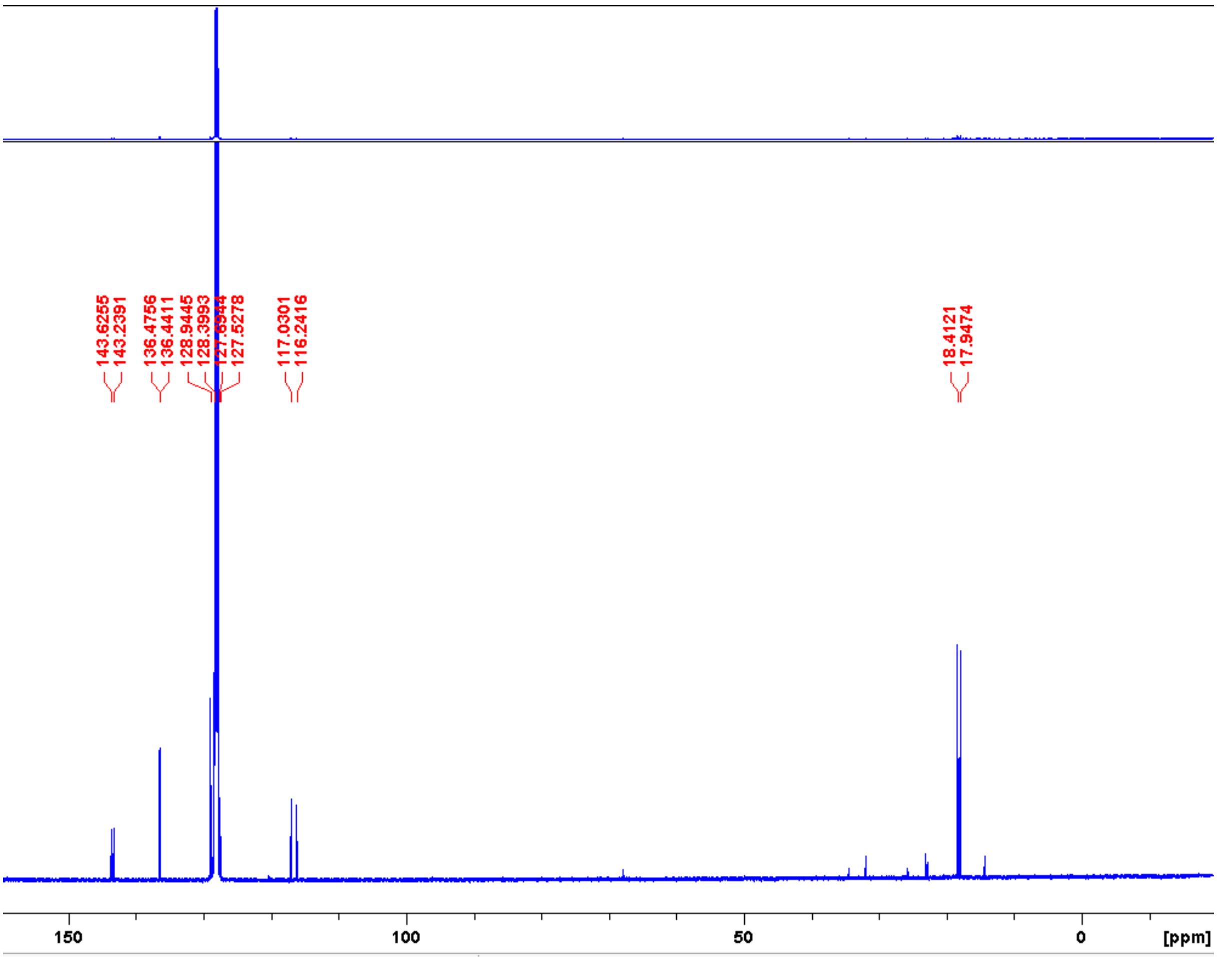

Figure S22: ^{13}C NMR spectrum of **3b** in C_6D_6.

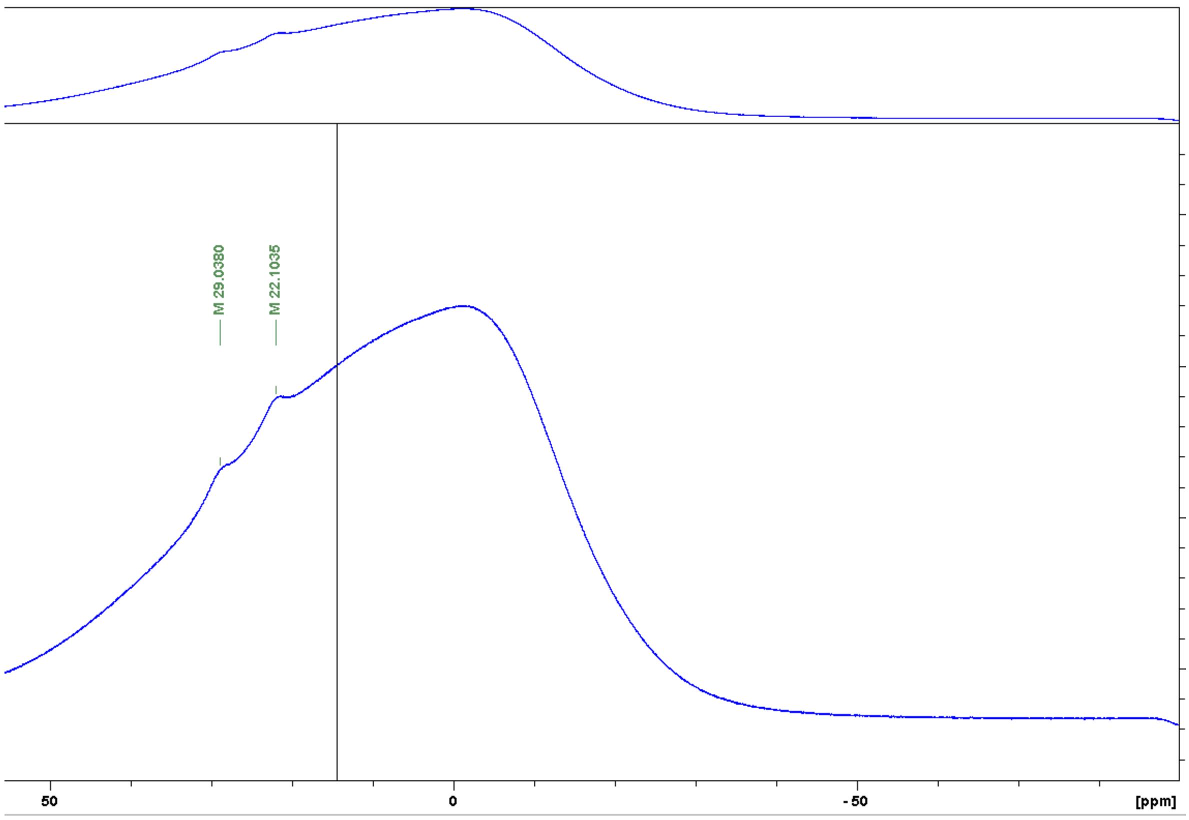

Figure S23: ^{11}B NMR spectrum of **3b** in C_6D_6.

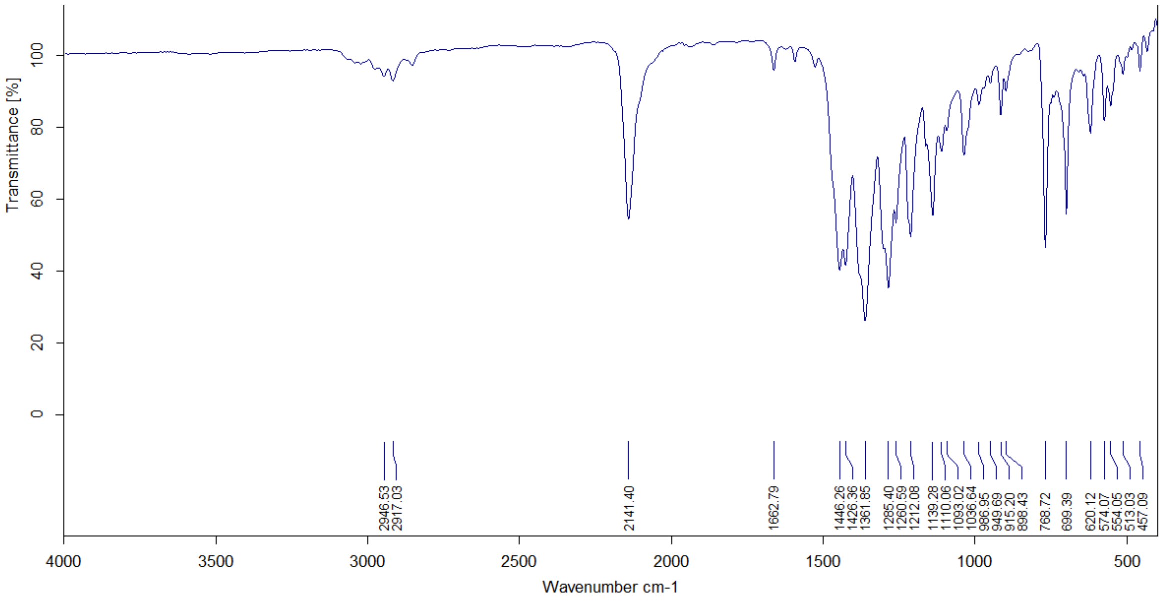

Figure S24: ATR-IR spectrum of **3b**.

Attempt to synthesize 1,8-diazido-2,5,9,12-tetra-*p*-tolyl-2,5,9,12-tetrahydro-1*H*,8*H*-[1,3,2,4]diazadiboreto[2,1-*b*:4,3-*b'*]bis([1,3,5,2,4]triazadiborepine) (3c)

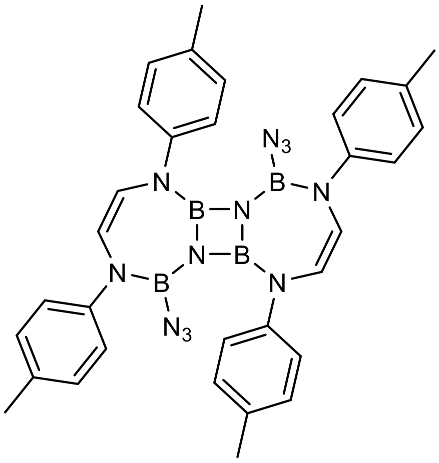

2c (32.1 mg, 93.8 µmol) was placed in a narrow schlenk flask. The atmosphere was removed *in vacuo* and the flask sealed under vacuum. The flask was heated up to 110 °C whereupon melting, gas evolution and a color change to green was observed. The analysis of the resulting products by ^{11}B and ^{1}H NMR spectroscopy did not show any selective reaction (in contrast to the both reactions above) and only an intractable mixture was obtained.

^{11}B NMR (160 MHz, C_6D_6): δ = 33.7 (s, br, starting material), 29.8–24.2 (br), 22.1 (br), 19 ppm (s).

Photolysis and pyrolysis of 1

A solution of **1** in C_6D_6 was placed overnight under a mercury vapor light, yielding partial conversion of the starting material, which was complete after a further *ca.* 8 h of photolysis.

^{11}B NMR (160 MHz, C_6D_6): δ = 26.0 and shoulder at 24.0 ppm. **^{1}H NMR** (400 MHz, C_6D_6): 2.56 (broad/complex signal) ppm.

The pyrolysis of **1** was performed by heating a mesitylene solution of **1** between 110 and 150 °C for several days. The selectivity of the pyrolysis reaction was comparable to that of the photolysis. The complete consumption of the starting material could be approached within days only at temperatures above 140 °C.

Reaction of tetrahalodiborane(4) derivatives with azide transfer reagents

Reactions were attempted using B_2F_4, $B_2Cl_4{\cdot}2SMe_2$, B_2Br_4, B_2I_4, $[PPh_4]_2[B_2Br_6]$ (speciates into $[PPh_4][B_2Br_5{\cdot}NCCH_3]$ in acetonitrile), $B_2Br_4{\cdot}2SMe_2$ as the diborane(4) substrates and

tetrabutylammonium azide ([TBA]N_3), [PPh_4]N_3 or trimethylsilyl azide (TMSN_3) as azide transfer reagents.

In a typical reaction, TMSN_3 (*ca.* 4x10^{-4} mol) was condensed over B_2X_4 (*ca.* 1x10^{-4} mol) in dichloromethane at –196 °C or over a dichloromethane solution of B_2X_4·2SMe_2 and the reaction was followed by NMR spectroscopy. Azide salts were then added to the resulting solution in order to generate the putative $[B_2X_{5-n}(N_3)_n]^-$ or $[B_2X_{6-n}(N_3)_n]^{2-}$. Alternatively, B_2X_4 was condensed onto a solution of [cation]N_3 in dichloromethane.

Reations usually show the complete conversion of the diborane(4) starting material. Most reactions show clear evidence for dinitrogen evolution by ^{14}N NMR spectroscopy. Except for reactions involving B_2F_4, the dinitrogen evolution is usually visible as vigorous effervescence shortly after the solvent has thawed (temperatures between –96 and *ca.* –40 °C). Typically, three resonances are found around 30 ppm in the ^{11}B NMR spectra of the reaction mixtures, the sharpest of which shifting to *ca.* 0 ppm upon addition of azide salts. This is indicative of a breakage of the B-B bond in the starting materials (signals are found at too high field – and are too sharp – to indicate the presence of diboranes(4)). The sharp signals found at *ca.* 0 ppm are indicative of the presence of $[B(N_3)_4]^-$.[3] In some larger-scale experiments, the amount of N_2 produced was determined by pressure measurements on a calibrated vacuum line, which revealed that *ca.* 50 to 65 mol% of N_2 was produced per mole of diborane. The presence of two broad resonances at *ca.* 30 ppm in ^{11}B NMR spectra in most experiments (more consistent with diboranes(4)) even after the addition of azide salts, in addition to the substoichiometric amount of N_2 produced, may be evidence that a certain amount of a azide compounds still bearing a B-B bond might be formed and trapped by adduct formation with a Lewis base (TMSN_3, $[N_3]^-$ or one of the boron azides formed) in the mixture. No direct evidence for such species has been found to date.

X-ray Crystallographic Details

The crystal data of **2a, 2b, 2c** and **3b** were collected on a BRUKER D8 QUEST diffractometer with a CMOS area detector and multi-layer mirror monochromated $Mo_{K\alpha}$ radiation. The crystal data of **3a** were collected on a BRUKER X8-APEX II diffractometer with a CCD area detector and multi-layer mirror monochromated $Mo_{K\alpha}$ radiation.

The structures were solved using the intrinsic phasing method,[4] refined with the SHELXL program[5] and expanded using Fourier techniques. All non-hydrogen atoms were refined anisotropically. Hydrogen atoms were included in structure factor calculations. All hydrogen atoms were assigned to idealized geometric positions and depicted as spheres of arbitrary radius. Ellipsoids are represented at the 50% probability level.

Crystallographic data have been deposited with the Cambridge Crystallographic Data Center as supplementary publication numbers CCDC 1984944-1984948. These data can be obtained free of charge from The Cambridge Crystallographic Data Centre *via* www.ccdc.cam.ac.uk/data_request/cif

MDBN3 (2a)

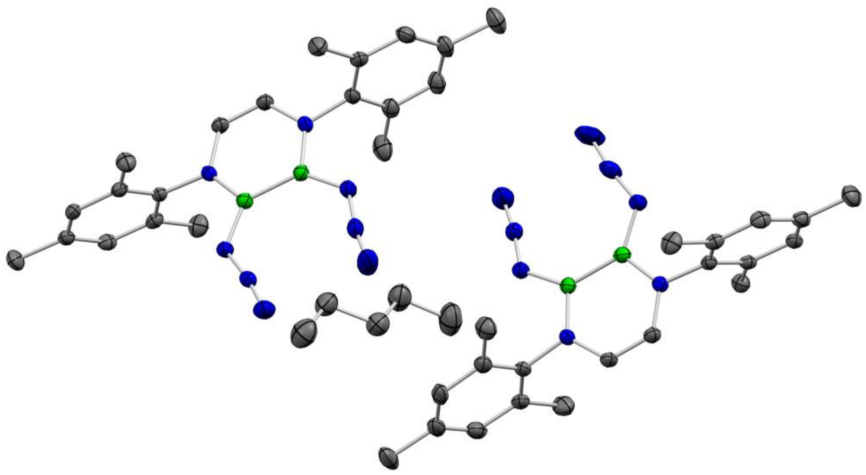

Figure S25: Solid-state structure of **2a**, showing the two crystallographically-independent molecules of **2a** and the cocrystallized pentane molecule making up the asymmetric unit. Hydrogen atoms are omitted for clarity.

Table S1: Structural determination parameters for **2a**.

Data	**ToTh156_a**
Empirical formula	$C_{45}H_{60}B_4N_{16}$
Formula weight (g·mol^{-1})	868.33
Temperature (K)	100(2)
Radiation, λ (Å)	$Mo_{K\alpha}$ 0.71073
Crystal system	Orthorhombic
Space group	$Pca2_1$
Unit cell dimensions	
a (Å)	13.585(6)
b (Å)	24.562(12)
c (Å)	14.869(7)
α (°)	90
β (°)	90
γ (°)	90
Volume (Å^3)	4961(4)
Z	4
Calculated density (Mg·m^{-3})	1.163
Absorbtion coefficient (mm^{-1})	0.072
F(000)	1848
Theta range for collection	2.193 to 26.020°
Reflections collected	33521
Independent reflections	8635
Minimum/maximum transmission	0.6493/0.7461
Refinement method	Full-matrix least-squares on F^2
Data / parameters / restraints	8635 / 600 / 1
Goodness-of-fit on F^2	1.038
Final R indices [I>2σ(I)]	R_1 = 0.0374, wR^2 = 0.0914
R indices (all data)	R_1 = 0.0461, wR^2 = 0.0972
Maximum/minimum residual electron density (e·Å^{-3})	0.216 / –0.257

XDBN3 (2b)

The two azide moieties were found in two conformations each, which were modeled as two independent two-part disorders with refined ratios of 84:16 and 74:26, respectively. The distances between B1/N101_4 and B1/N101_3 were restrained to be the same with the SADI similarity restraint. The displacement parameters of pairs of atoms N101_3/N101_4; N102_3/N102_4; N103_3/N103_4 of the second disordered azide moiety were respectively constrained to the same value with EADP keyword. The displacement parameters of atoms N101_3 > N103_4 and N101_1 > N103_2 of the two disordered azide moieties were respectively restrained to the same value with the similarity restraint SIMU. The atomic displacement parameters of atoms N101_3 > N103_4 were restrained with the RIGU keyword in the ShelXL input ('enhanced rigid bond' restraint for all bonds in the connectivity list. Values of 0.003 for both parameters s1 and s2 were used). The 1-2 and 1-3 distances in N101_1 > N103_1/N101_2 > N103_2 and N101_3 > N103_3/N101_4 > N103_4 were respectively restrained to the same values with SAME.

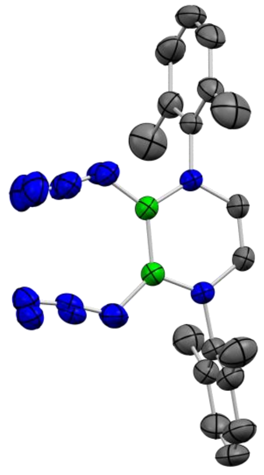

Figure S26: Solid-state structure of **2b** showing the disordered azide moieties. Hydrogen atoms are omitted for clarity.

Table S2: Structural determination parameters for **2b**.

Data	**ToTh167_a**
Empirical formula	$C_{18}H_{20}B_2N_8$
Formula weight (g·mol^{-1})	370.04
Temperature (K)	100(2)
Radiation, λ (Å)	$Mo_{K\alpha}$ 0.71073
Crystal system	Orthorhombic
Space group	*Pbca*
Unit cell dimensions	
a (Å)	13.305(3)
b (Å)	14.563(4)
c (Å)	20.683(9)
α (°)	90
β (°)	90
γ (°)	90
Volume (Å^3)	4008(2)
Z	8
Calculated density (Mg·m^{-3})	1.227
Absorbtion coefficient (mm^{-1})	0.078
F(000)	1552
Theta range for collection	2.295 to 25.884°
Reflections collected	28082
Independent reflections	3878 [R_{int} = 0.0336]
Minimum/maximum transmission	0.1944/0.2271
Refinement method	Full-matrix least-squares on F^2
Data / parameters / restraints	3878 / 295 / 157
Goodness-of-fit on F^2	1.044
Final R indices [I>2σ(I)]	R_1 = 0.0442, wR^2 = 0.1189
R indices (all data)	R_1 = 0.0554, wR^2 = 0.1285
Maximum/minimum residual electron density (e·Å^{-3})	0.229 / –0.166

pTolDBN3 (2c)

One azide moiety was found to be in two conformations, which was modeled as a two-part disorder with a refined ratio of 0.51:0.49. The displacement parameters of atoms N101_1 > N103_2 of the disordered azide moiety were restrained to the same value with similarity restraint SIMU. The atomic displacement parameters of atoms N101_1 > N103_2 were restrained with the RIGU keyword in the ShelXL input ('enhanced rigid bond' restraint for all bonds in the connectivity list. Values of 0.003 for both parameters s1 and s2 were used). The 1-2 and 1-3 distances in N101 > N103 in residues 1 and 2 (disordered azide moiety) were restrained to the same values with SAME.

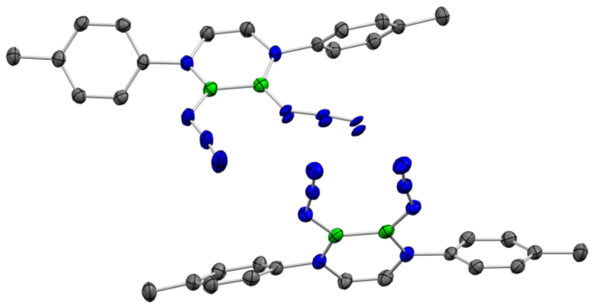

Figure S27: Solid-state structure of **2c**, showing the two molecules of **2c** making up the asymmetric unit and the disordered azide moiety. Hydrogen atoms are omitted for clarity.

Table S3: Structural determination parameters for: **2c**.

Data	**ToTh182_a**
Empirical formula	$C_{16}H_{16}B_2N_8$
Formula weight (g·mol^{-1})	341.99
Temperature (K)	100(2)
Radiation, λ (Å)	$Mo_{K\alpha}$ 0.71073
Crystal system	Triclinic
Space group	$P\bar{1}$
Unit cell dimensions	
a (Å)	10.224(3)
b (Å)	12.520(4)
c (Å)	14.988(5)
α (°)	99.774(14)
β (°)	99.773(14)
γ (°)	109.041(15)
Volume (Å^3)	1734.2(10)
Z	4
Calculated density (Mg·m^{-3})	1.310
Absorbtion coefficient (mm^{-1})	0.084
F(000)	712
Theta range for collection	2.246 to 26.022°
Reflections collected	17673
Independent reflections	6791
Minimum/maximum transmission	0.4010/0.5401
Refinement method	Full-matrix least-squares on F^2
Data / parameters / restraints	6791 / 501 / 87
Goodness-of-fit on F^2	1.028
Final R indices [I>2σ(I)]	R_1 = 0.0555, wR^2 = 0.1231
R indices (all data)	R_1 = 0.0938, wR^2 = 0.1437
Maximum/minimum residual electron density (e·Å^{-3})	0.240 / –0.264

MDBN3dT 3a

One azide moiety was found to be in two conformations, which was modeled as a two-part disorder with a refined ratio of 0.66:0.34. The displacement parameters of atoms N101_1 > N103_2 of the disordered azide moiety were restrained to the same value with the similarity restraint SIMU. The 1-2 and 1-3 distances in N101 > N103 in residues 1 and 2 (disordered azide moiety) were restrained to the same values with SAME.

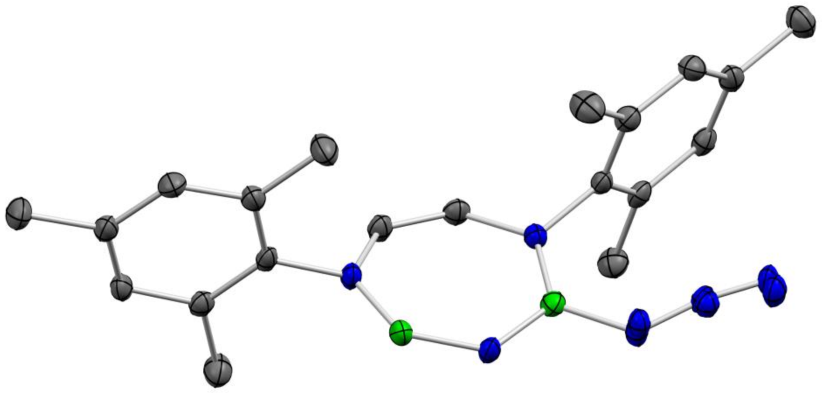

Figure S28: Solid-state structure of **3a**, showing the half-molecule of **3a** that makes up the asymmetric unit (the whole molecule is generated by symmetry) and the disordered azide moiety. Hydrogen atoms are omitted for clarity.

Table S4: Structural determination parameters for: **3a.**

Data	**ToTh193_a**
Empirical formula	$C_{40}H_{48}B_4N_{12}$
Formula weight (g·mol^{-1})	740.14
Temperature (K)	100(2)
Radiation, λ (Å)	$Mo_{K\alpha}$ 0.71073
Crystal system	Monoclinic
Space group	$P2_1/n$
Unit cell dimensions	
a (Å)	11.251(8)
b (Å)	13.966(8)
c (Å)	12.904(8)
α (°)	90
β (°)	90.87(4)
γ (°)	90
Volume (Å^3)	2028(2)
Z	2
Calculated density (Mg·m^{-3})	1.212
Absorbtion coefficient (mm^{-1})	0.074
F(000)	784
Theta range for collection	2.149 to 26.019°
Reflections collected	17596
Independent reflections	3989
Minimum/maximum transmission	0.1581/0.2277
Refinement method	Full-matrix least-squares on F^2
Data / parameters / restraints	3989 / 287 / 69
Goodness-of-fit on F^2	1.027
Final R indices [I>2σ(I)]	R_1 = 0.0420, wR^2 = 0.0963
R indices (all data)	R_1 = 0.0610, wR^2 = 0.1063
Maximum/minimum residual electron density (e·Å^{-3})	0.302 / –0.224

XDBN3dT (3b)

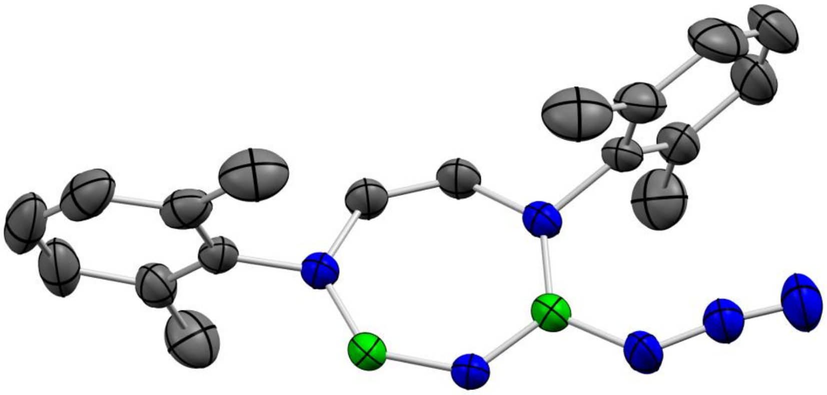

Figure S29: Solid-state structure of **3b**, showing the half-molecule of **3b** that makes up the asymmetric unit (the whole molecule is generated by symmetry). Hydrogen atoms are omitted for clarity.

Table S5: Structural determination parameters for: **3b.**

Data	**ToTh165_a**
Empirical formula	$C_{36}H_{40}B_4N_{12}$
Formula weight (g·mol^{-1})	684.04
Temperature (K)	100(2)
Radiation, λ (Å)	$Mo_{K\alpha}$ 0.71073
Crystal system	Monoclinic
Space group	$P2_1/n$
Unit cell dimensions	
a (Å)	15.924(4)
b (Å)	6.9227(14)
c (Å)	17.046(5)
α (°)	90
β (°)	93.844(15)
γ (°)	90
Volume (Å^3)	1874.9(8)
Z	2
Calculated density (Mg·m^{-3})	1.212
Absorbtion coefficient (mm^{-1})	0.075
F(000)	720
Theta range for collection	2.395 to 26.022°
Reflections collected	23341
Independent reflections	3687
Minimum/maximum transmission	0.3761/0.4030
Refinement method	Full-matrix least-squares on F^2
Data / parameters / restraints	3687 / 239 / 0
Goodness-of-fit on F^2	1.099
Final R indices [I>2σ(I)]	R_1 = 0.0521, wR^2 = 0.1306
R indices (all data)	R_1 = 0.0587, wR^2 = 0.1348
Maximum/minimum residual electron density (e·Å^{-3})	0.206 / –0.141

Computational Details

Initially, we performed geometry optimization calculations on the diazidodiborane(4) **1**, the mesityl-substituted diazido diborane **2a** and its isomer **2a'**, the formal transient nitrenes with singlet (**^{1}2a^{nit}**) and triplet (**^{3}2a^{nit}**) multiplicities, the transient seven-membered ring cyclic iminoborane **2a''**, and the mesityl-substituted diazadiboretidine **3a.** The optimizations of **2a** and **3a** were performed starting from the experimental solid-state geometries. For **1**, since no solid-state structure was obtained, we performed a systematic conformational search changing the initial orientation of the azide and amido groups in order to identify the most stable conformer. All structures were optimized using density functional theory (DFT) with the B3LYP[6] functional and 6-31+G*[7] basis set.

We then performed stability tests[8] on the single-determinant restricted closed-shell wavefunctions of the optimized geometries at singlet multiplicities. Apart from the singlet nitrene **^{1}2a^{nit}**, we did not observe internal instability for the singlet closed-shell solution of any of the systems, revealing that they do not present biradical character. For **^{1}2a^{nit}**, all attempts at optimizing the structure with a restricted closed-shell wavefunction failed, and the calculations converged directly to the cyclic iminoborane **2''**. The stability test of **^{1}2a^{nit}** starting from the optimized triplet nitrene **^{3}2a^{nit}** structure revealed that the closed-shell wavefunction has a restricted Hartree-Fock (RHF)/unrestricted Hartree-Fock (UHF) instability, which impeded geometry optimization. This was overcome by using a stable open-shell UDFT wavefunction, which allowed the straightforward geometry optimization of **^{1}2a^{nit}**. Our DFT results, therefore, reveal that the singlet nitrene possesses a biradical character.

In order to characterize the optimized geometries as minimum energy structures, we performed Hessian calculations and thermochemical analyses also at the (U)B3LYP/6-31+G* level. All geometries were characterized as minimum energy structures in their respective potential energy surfaces by vibrational frequency analysis, as all Hessian eigenvalues are positive. We applied a scaling factor of 0.94[9] on the computed vibrational frequencies as well as a Gaussian broadening fitting (band width at half-height: 50 cm^{-1}) in order to compare them with the experimental IR spectra. Single-point calculations at the B3LYP/6-311++G**[7a, 10] level of theory starting from the optimized structures were obtained for generating molecular orbital diagrams for **1, 2a, 2a''** and **3a**.

In order to address the presence of aromatic character in the cyclic diborane species, we performed nucleus-independent chemical shift (NICS)[11] calculations for **2a** and benzene (**5**)

at the same level of theory (B3LYP/6-311++G**//B3LYP/6-31+G*) (Figure S34). The out-of-plane zz component of NICS was obtained for several distances by placing ghost atoms up to 6 Å above and below the ring plane, with a step size of 0.1 Å. A negative value of the NICS(1) magnetic shielding, which is calculated for a distance of 1 Å from the ring plane, is indicative of aromaticity. By plotting the zz component of NICS as a function of the distance from the ring plane, we obtain the NICS-scan profile. The presence of a minimum in the NICS-scan curve also indicates aromatic character.[11c]

All calculations were carried out with Gaussian 16, Revision B.01.[12] Pictures of molecular structures, orbitals and densities were visualized and generated with Chemcraft,[13] ADFView and CYLview.

In order to confirm the open-shell character of the singlet nitrene **^{1}2a^{nit}**, we performed single-point calculations using high-level complete active space self-consistent field (CASSCF)[14] and N-electron valence state second-order perturbation theory (NEVPT2)[15] on a reduced model of the nitrene. In this approach, methyl groups replaced the mesityl substituents at both N atoms, leading to the open-shell singlet (**^{1}2d^{nit}**) and triplet (**^{3}2d^{nit}**) nitrenes. The calculations were performed for the fully optimized **^{1}2d^{nit}** and **^{3}2d^{nit}** structures at the B3LYP/6-31+G* level of theory. Further single-point calculations at the CASSCF(8,8) and NEVPT2(8,8) levels with cc-pVDZ[16] basis set were performed. We obtained the singlet-triplet gap for each of those levels and estimated the biradical character index (y^{PUHF}) in the spin-projected unrestricted Hartree-Fock (PUHF) formalism.[17] The y^{PUHF} index is defined by the weight of the doubly-excited configuration within a multiconfigurational self-consistent field (MCSCF) approach, and is given by the following expression:

$$y^{PUHF} = 1 - \frac{2T}{1 + T^2} \qquad (S1)$$

where T is the orbital overlap of the highest occupied natural orbital (HONO) and the lowest unoccupied natural orbital (LUNO), and is obtained by the occupation numbers, η, of the respective UHF natural orbitals:

$$T = \frac{n_{HONO} - n_{LUNO}}{2} \qquad (S2)$$

The y^{PUHF} index can vary from 0 (closed-shell system) to 1 (pure biradical state). The calculations were performed using the ORCA 4.1.1 software.[18]

We also investigated the reaction mechanism starting from **2a** that leads to the dimeric product **3a**. We tried two distinct approaches: the first involved the formation of a transient nitrene species, while in the second the formation of the iminoborane seven-membered ring **2a''** is achieved directly after N_2 liberation without a previous nitrene formation. Geometry optimizations, stability tests and Hessian calculations were performed at the B3LYP/6-31+G* for all transition states (TSs) and intermediates. Intrinsic reaction coordinate (IRC)[19] calculations were also performed in order to confirm the connectivity between transition states and their respective reactants and products. Single-point calculations at the B3LYP/6-311++G** level of theory starting from the optimized minimum and TS structures were performed in order to correct the electronic energies. We also took into account dispersion interactions by combining Grimme's empirical dispersion correction GD3[20] with the B3LYP functional to all of the calculated minima and TSs. For computing the ΔG of solvation, we employed the solvation model based on density (SMD)[21] technique with mesitylene (ε = 2.2650) as solvent. The entropic contributions obtained within the ideal gas approximation at p = 1 atm for molecules in solution are overestimated, which affects especially associative/dissociative reaction steps.[22] In order to better describe the liquid state, the Gibbs free energies reported in this work incorporate zero-point energy, thermal and entropic contributions that were evaluated at 298.15 K and 176 atm ($p = \rho_{mesitylene}RT$; $\rho_{mesitylene}$ = 0.8637 g cm^{-3}), following the procedure described by Martin et al.[23]

Finally, we compared the thermochemistry of the dimerization of two iminoborane rings **2a''** with the one that could hypothetically be achieved from the junction of two triplet nitrenes **^{3}2a^{nit}** leading to N_2-bridged compounds. For that purpose, we performed the same calculations as the ones described in the previous paragraphs for the *cis*- and *trans*-N_2-bridged bis(azido diborane) compounds **4** and **4b**.

Conformational Analysis of 1 (B3LYP/6-31+G*)

Table S6: Structure and relative energies of distinct conformers of **1** (B3LYP/6-31+G*).

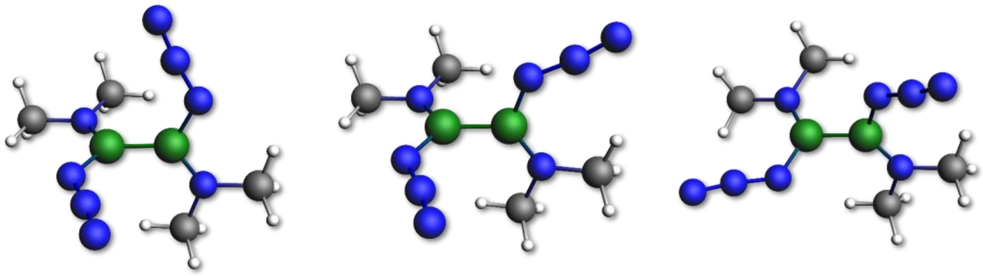

	**1**	**1b**	**1c**
E+ZPE (a.u.)	–647.2093	–647.2015	–647.1939
Δ(E+ZPE) (kcal mol^{-1})	0.0	4.9	9.7

Geometrical Parameters

Table S7: Comparison of the geometrical parameters (Å for bond lengths) of the X-ray and the computed equilibrium structure of **2a** (B3LYP/6-31+G*).

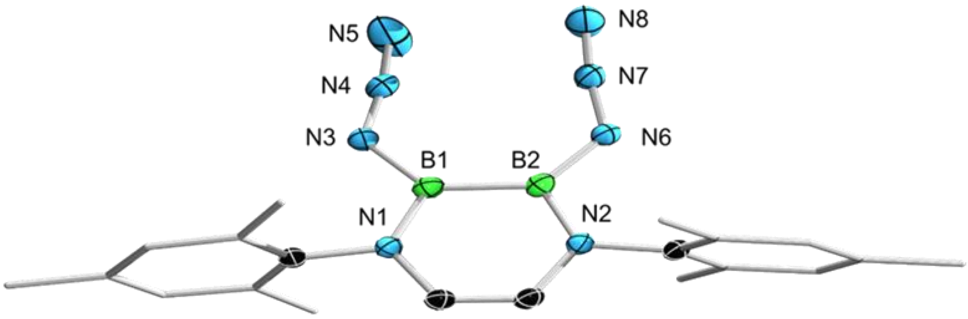

2a

	2a (X-ray)	2a (Calcd)
B1–B2	1.670(4)	1.693
B1–N1	1.419(3)	1.435
B2–N2	1.414(3)	1.435
B1–N3	1.468(4)	1.459
B2–N6	1.461(3)	1.459

Table S8: Comparison of the geometrical parameters (Å for bond lengths, ° for bond angles) of the X-ray and the computed equilibrium structures of **3a**.

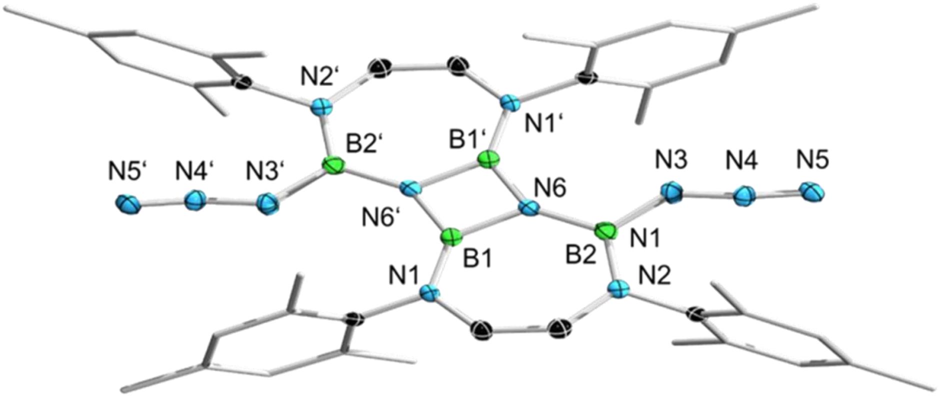

3a

	3a (X-ray)	3a (Calcd)
B1–N6	1.469(2)	1.475
B1–N6'	1.463(2)	1.475
B1–N1	1.397(2)	1.415
B2–N6	1.421(2)	1.427
B2–N2	1.421(2)	1.438
a(N6'–B1–N6)	96.4(1)	96.5
a(B1–N6–B1')	83.6(1)	83.4

Hessian Calculations and Thermochemical Analysis

Table S9: Harmonic frequencies (cm^{-1}), reduced masses (amu), force constants (mDyne Å^{-1}) and IR intensities (km mol^{-1}) for the nine lowest vibrational frequency modes of **1**, **1b** and **1c**.

Vibrational Mode	Frequencies	Reduced Masses	Force Constants	IR Intensities
**1**				
1	13.4824	6.4491	0.0007	1.0789
2	60.8369	4.4583	0.0097	1.1056
3	64.7936	4.7800	0.0118	0.0979
4	77.4124	3.9568	0.0140	0.1126
5	78.3307	4.0378	0.0146	0.1416
6	103.1977	4.6198	0.0290	0.0536
7	124.7299	5.5972	0.0513	0.0873
8	150.5156	4.3144	0.0576	0.1546
9	158.9780	2.9701	0.0442	0.2163
**1b**				
1	10.0316	6.5264	0.0004	1.0352
2	24.2127	3.8426	0.0013	0.3650
3	56.3998	4.0691	0.0076	0.7914
4	71.5319	3.1667	0.0095	0.1428
5	79.9981	4.8248	0.0182	0.2820
6	103.4228	4.5718	0.0288	0.3125
7	124.9685	1.4703	0.0135	0.1279
8	139.6705	4.8805	0.0561	1.1515
9	153.6930	3.7357	0.0520	0.0951
**1c**				
1	21.7543	5.5291	0.0015	1.7155
2	37.3234	5.3046	0.0044	0.0296
3	43.7213	3.9376	0.0044	0.5401
4	58.0838	3.1688	0.0063	0.0030
5	73.1883	3.6854	0.0116	1.2664
6	102.6855	3.4708	0.0216	1.4382
7	108.4916	2.2029	0.0153	0.7030
8	141.4327	1.6921	0.0199	1.3323
9	157.9883	1.7787	0.0262	0.1436

Table S10: Thermochemical analysis of **1**, **1b**, and **1c** (T = 298.15 K; p = 176 atm). All values are in Hartree.

	**1**	**1b**	**1c**
**B3LYP/6-31+G***			
Electronic energy	–647.4129	–647.4048	–647.3970
Zero-point correction	0.2035	0.2032	0.2030
Thermal correction to energy	0.2201	0.2200	0.2199
Thermal correction to enthalpy	0.2210	0.2209	0.2208
Thermal correction to Gibbs free energy	0.1628	0.1611	0.1613
Sum of electronic and zero-point energies	–647.2093	–647.2015	–647.1939
Sum of electronic and thermal energies	–647.1928	–647.1848	–647.1771
Sum of electronic and thermal enthalpies	–647.1918	–647.1839	–647.1762
Sum of electronic and thermal free energies	–647.2500	–647.2437	–647.2357
**B3LYP/6-311++G**//B3LYP/6-31+G***			
Electronic energy	–647.5699	-	-

Table S11: Harmonic frequencies (cm^{-1}), reduced masses (amu), force constants (mDyne $Å^{-1}$) and IR intensities (km mol^{-1}) for the nine lowest vibrational frequency modes of **2a**, **2a''** and **3a**.

Vibrational Mode	Frequencies	Reduced Masses	Force Constants	IR Intensities
**2a**				
1	11.4354	1.0180	0.0001	0.1533
2	15.5161	7.7229	0.0011	0.0006
3	18.6734	1.0438	0.0002	0.2357
4	20.4083	3.7690	0.0009	0.0092
5	24.8512	4.2276	0.0015	0.0043
6	31.7729	4.7278	0.0028	0.2206
7	36.4830	4.2696	0.0033	0.2542
8	47.9288	5.6030	0.0076	0.3893
9	63.7101	5.8105	0.0139	0.0000
**2a''**				
1	15.9341	1.0511	0.0002	0.1510
2	17.2430	2.6055	0.0005	0.0312
3	21.9978	1.1209	0.0003	0.2352
4	25.1137	4.2163	0.0016	0.1115
5	30.0117	4.2102	0.0022	0.1075
6	32.4008	5.2169	0.0032	0.3243
7	49.3914	5.5812	0.0080	0.3035
8	60.1378	5.2868	0.0113	0.2420
9	68.4149	4.8015	0.0132	0.4957
**3a**				
1	8.2837	5.0370	0.0002	0.0002
2	12.0692	4.7575	0.0004	0.0071
3	14.5523	5.0172	0.0006	0.3061

4	16.3017	4.4470	0.0007	0.0019
5	17.5125	4.5503	0.0008	0.0796
6	29.6657	4.0813	0.0021	0.0847
7	30.1040	3.7996	0.0020	0.0134
8	31.8343	3.6970	0.0022	0.0113
9	33.3631	3.8032	0.0025	0.2017

Table S12: Thermochemical analysis of **2a**, **2a''** and **3a** (T = 298.15 K; p = 176 atm). All values are in Hartree.

	**2a**	**2a''**	**3a**
**B3LYP/6-31+G***			
Electronic energy	–1264.3930	–1154.9152	–2309.9891
Zero-point correction	0.4299	0.4201	0.8453
Thermal correction to energy	0.4609	0.4489	0.9034
Thermal correction to enthalpy	0.4618	0.4498	0.9044
Thermal correction to Gibbs free energy	0.3670	0.3614	0.7478
Sum of electronic and zero-point energies	–1263.9631	–1154.4951	–2309.1438
Sum of electronic and thermal energies	–1263.9322	–1154.4663	–2309.0857
Sum of electronic and thermal enthalpies	–1263.9312	–1154.4654	–2309.0847
Sum of electronic and thermal free energies	–1264.0260	–1154.5538	–2309.2413
**B3LYP/6-311++G**//B3LYP/6-31+G***			
Electronic energy	–1264.6766	–1155.1728	–2310.4992

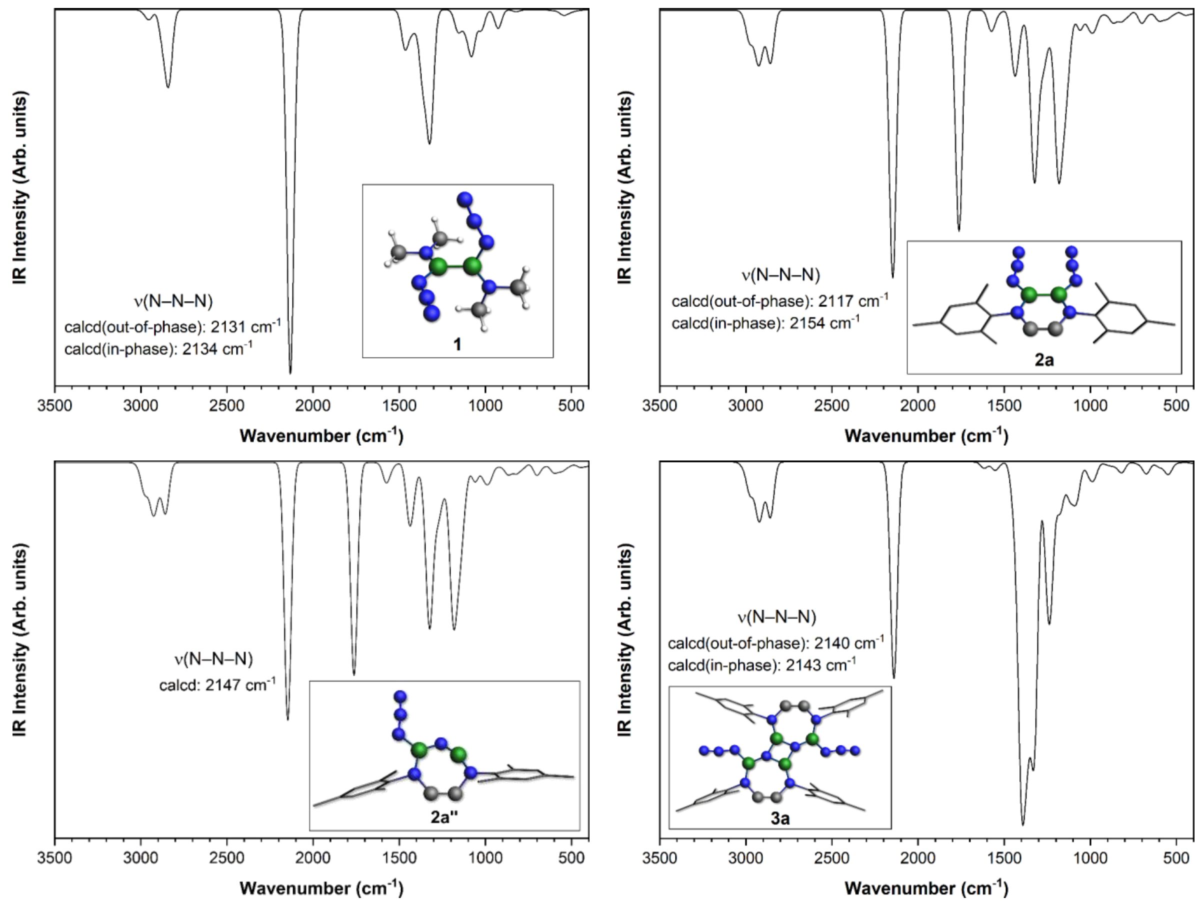

Figure S30: Computed IR spectra of **1**, **2a, 2a''** and **3a** at the B3LYP/6-31+G* level of theory. A precomputed vibrational scaling factor of 0.94[9] was used for the frequencies.

Table S13: Harmonic frequencies (cm^{-1}), reduced masses (amu), force constants (mDyne $Å^{-1}$) and IR intensities (km mol^{-1}) for the nine lowest vibrational frequency modes of the singlet (**^{1}2a^{nit}**) and triplet (**^{3}2^{nit}**) nitrene.

Vibrational Mode	Frequencies	Reduced Masses	Force Constants	IR Intensities
**Singlet nitrene (^{1}2a^{nit})**				
1	13.6157	3.9111	0.0004	0.1472
2	19.8320	3.8960	0.0009	0.0961
3	22.9399	1.0274	0.0003	0.2349
4	27.4887	1.0149	0.0005	0.1925
5	30.0920	4.6085	0.0025	0.3539
6	36.4261	4.2296	0.0033	0.3626
7	51.5453	8.2412	0.0129	4.5633
8	51.9298	5.2094	0.0083	0.5603
9	78.1859	5.2582	0.0189	0.7821

Triplet nitrene (^{3}2a^{nit})				
1	14.6975	3.5761	0.0005	0.1199
2	19.7048	1.2316	0.0003	0.3498
3	20.1131	2.3784	0.0006	0.0066
4	23.2932	1.0331	0.0003	0.1807
5	29.9778	4.7444	0.0025	0.2772
6	36.3815	4.2186	0.0033	0.3397
7	51.8778	5.2138	0.0083	0.4868
8	54.3959	7.7164	0.0135	3.0132
9	77.2348	5.2725	0.0185	0.5204

Table S14: Thermochemical analysis of **^{1}2a^{nit}** and **^{3}2a^{nit}** (T = 298.15 K; p = 176 atm). All values are in Hartree.

	**^{1}2a^{nit}**	**^{3}2a^{nit}**
**B3LYP/6-31+G***		
Electronic energy	–1154.8154	–1154.8269
Zero-point correction	0.4180	0.4181
Thermal correction to energy	0.4471	0.4472
Thermal correction to enthalpy	0.4481	0.4482
Thermal correction to Gibbs free energy	0.3590	0.3579
Sum of electronic and zero-point energies	–1154.3974	–1154.4088
Sum of electronic and thermal energies	–1154.3683	–1154.3797
Sum of electronic and thermal enthalpies	–1154.3674	–1154.3787
Sum of electronic and thermal free energies	–1154.4564	–1154.4690
**B3LYP/6-311++G**//B3LYP/6-31+G***		
Electronic energy	–1155.0710	–1155.0824
G + dispersion + solvent effects	–1154.7872	–1154.7981

Table S15: Harmonic frequencies (cm^{-1}), reduced masses (amu), force constants (mDyne Å^{-1}) and IR intensities (km mol^{-1}) for the nine lowest vibrational frequency modes of the *cis*- (**4**) and *trans*- (**4b**) N_2-bridged bis(azidodiborane) compounds.

Vibrational Mode	Frequencies	Reduced Masses	Force Constants	IR Intensities
**4**				
1	9.4418	5.2013	0.0003	0.0130
2	10.8586	5.3186	0.0004	0.0646
3	15.3755	1.0471	0.0001	0.2219
4	15.9710	1.1722	0.0002	0.3046
5	16.2287	3.1628	0.0005	0.2322
6	18.4482	3.3793	0.0007	0.1271
7	20.7779	3.4790	0.0009	0.0770
8	26.2423	3.1179	0.0013	0.0329
9	27.3606	4.4720	0.0020	2.3445
**4b**				
1	5.9944	5.2411	0.0001	0.0939
2	10.3382	5.5349	0.0003	0.1079
3	17.1288	2.6860	0.0005	0.2195
4	18.8573	1.1614	0.0002	0.1443
5	19.4777	2.1155	0.0005	0.2900
6	20.3167	2.7143	0.0007	0.0610
7	21.3201	1.6527	0.0004	0.1415
8	25.5605	4.0056	0.0015	0.4719
9	26.2995	1.0774	0.0004	0.1460

Table S16: Thermochemical analysis of the *cis*- (**4**) and *trans*- (**4b**) N_2-bridged bis(azidodiborane) compounds (T = 298.15 K; p = 176 atm). All values are in Hartree.

	**4**	**4b**
**B3LYP/6-31+G***		
Electronic energy	–2309.8021	–2309.8071
Zero-point correction	0.8419	0.8423
Thermal correction to energy	0.9011	0.9012
Thermal correction to enthalpy	0.9021	0.9021
Thermal correction to Gibbs free energy	0.7413	0.7425
Sum of electronic and zero-point energies	–2308.9602	–2308.9648
Sum of electronic and thermal energies	–2308.9009	–2308.9059
Sum of electronic and thermal enthalpies	–2308.9000	–2308.9049
Sum of electronic and thermal free energies	–2309.0608	–2309.0646
**B3LYP/6-311++G**//B3LYP/6-31+G***		
Electronic energy	–2310.3142	–2310.3189
G + dispersion + solvent effects	–2309.7384	–2309.7380

Table S17: Comparison of the geometrical structures of the *cis*- (**4a**) and *trans*- (**4a'**) N_2-bridged bis(azidodiborane) compounds (B3LYP/6-31+G*).

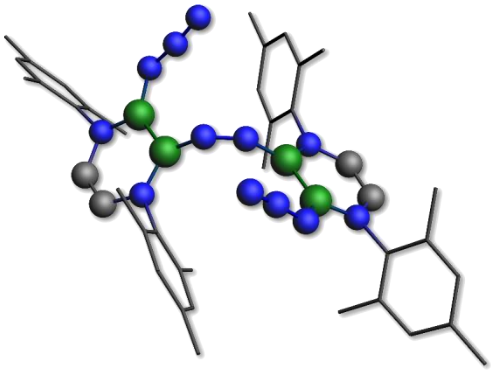

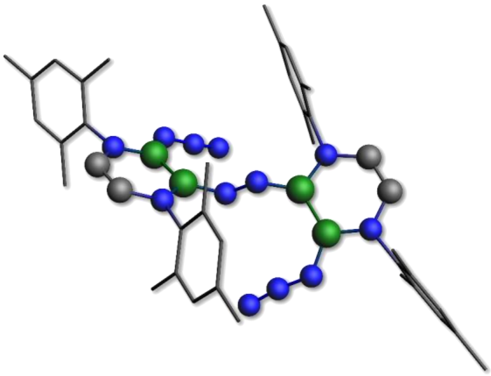

	**4a**	**4a'**

Molecular Orbital Diagrams and Spin Densities

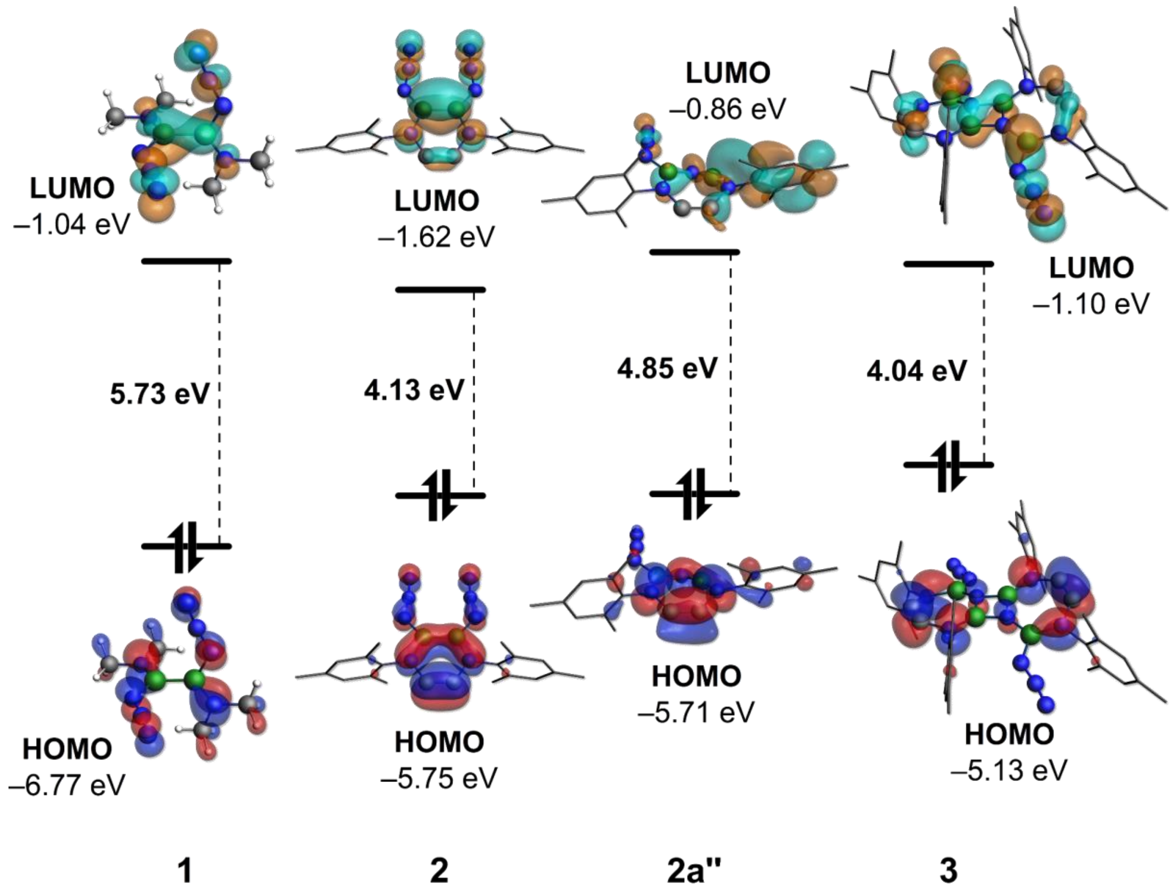

Figure S31: Molecular orbital diagrams of **1**, **2a**, **2a''** and **3a** at the B3LYP/6-311++G**//B3LYP/6-31+G* level of theory.

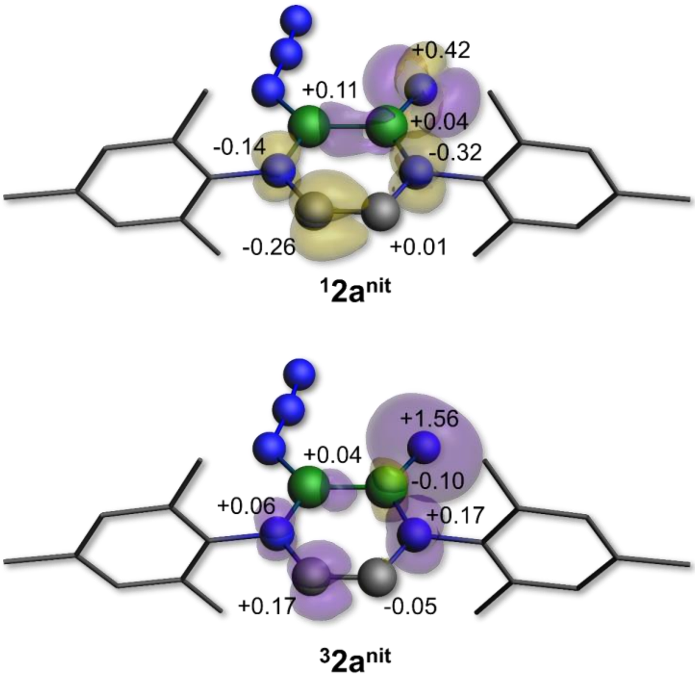

Figure S32: Spin densities of the singlet (**^{1}2a^{nit}**) and triplet (**^{3}2^{nit}**) nitrenes at the B3LYP/6-311++G**//B3LYP/6-31+G* level of theory.

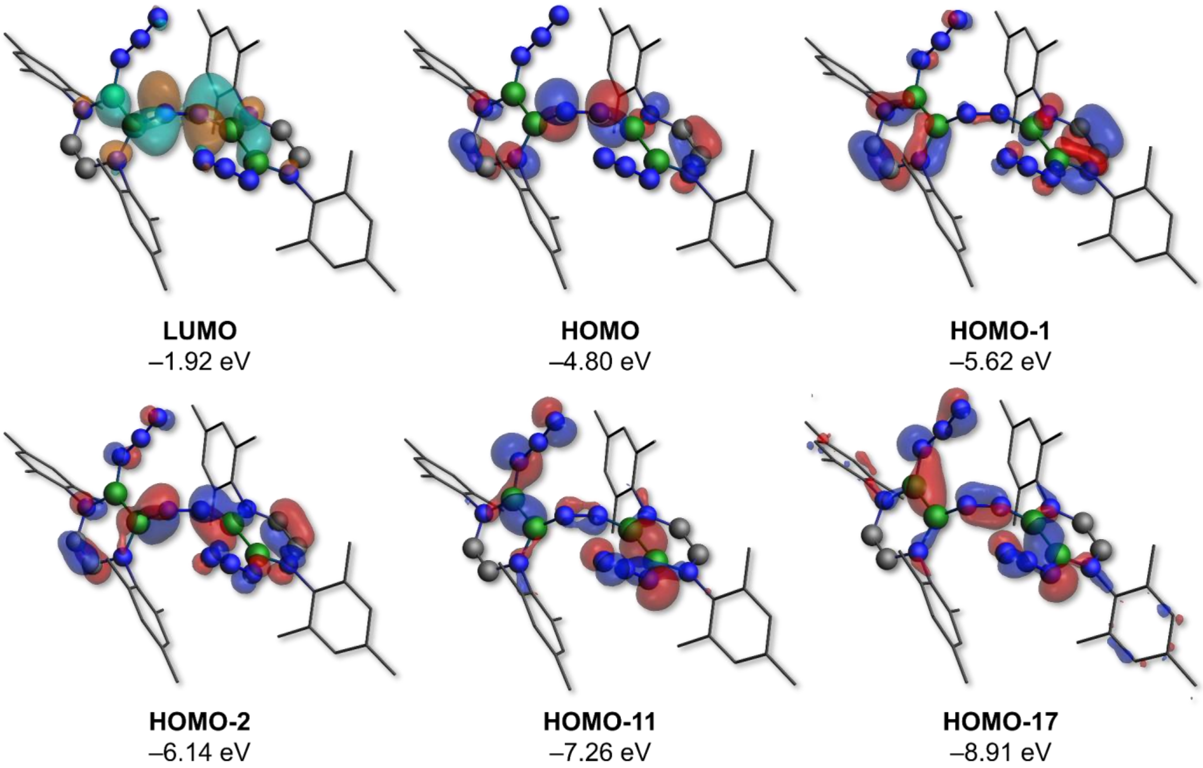

Figure S33: Selected molecular orbital diagrams of **4** at the B3LYP/6-311++G**//B3LYP/6-31+G* level of theory.

Nucleus-Independent Chemical Shift

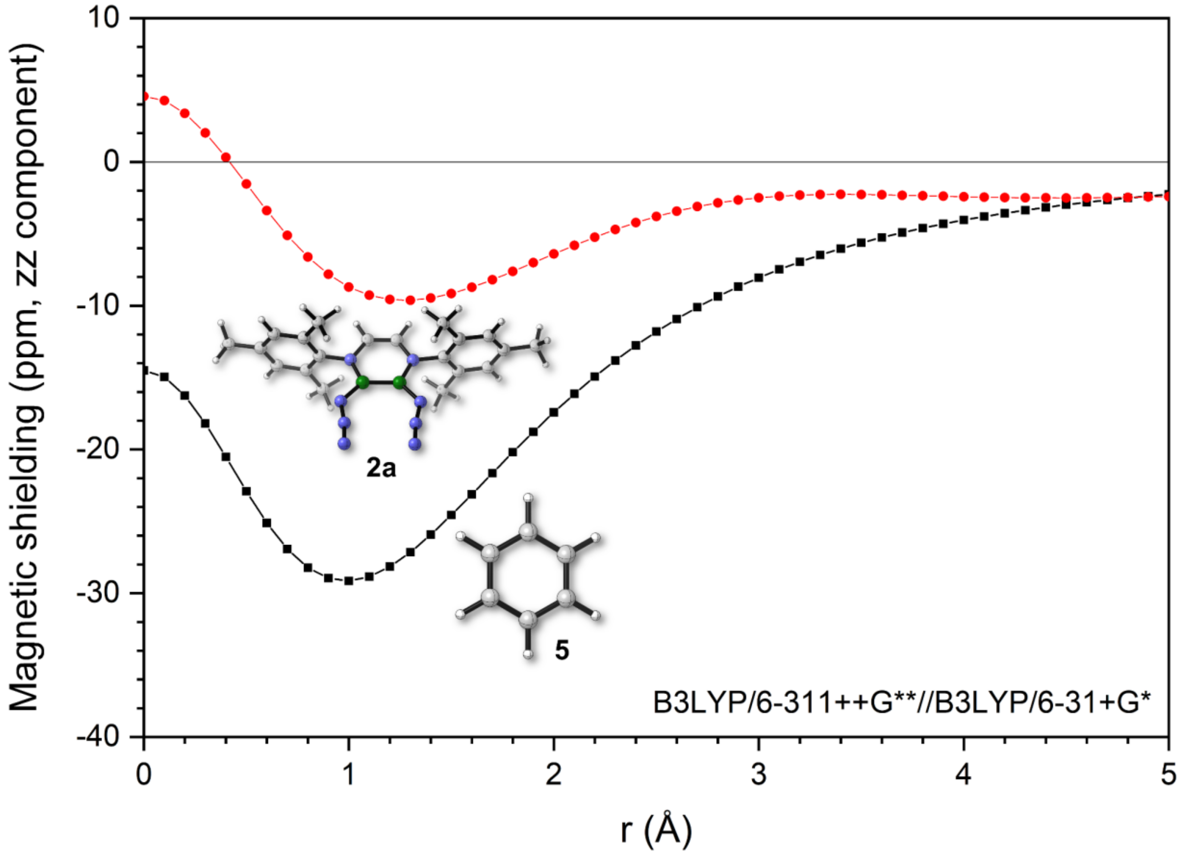

Figure S34: NICS-scan results (out-of plane zz component, ppm) for **2a** and **5** as a function of the distance from the ring plane. NICS(1) values for **2a**: –2.9 ppm (isotropic); –8.7 ppm (zz component). NICS(1) values for benzene (**5**): –10.2 ppm (isotropic); –29.1 ppm (zz component).

CASSCF and NEVPT2 Results

Table S18: CASSCF/NEVPT2 results of ^{1}**2d**^{nit} and ^{3}**2d**^{nit} using cc-pVDZ basis set. The active space is composed of eight electrons and eight orbitals.

	Minimal model
**CASSCF(8,8)**	
Electronic energy (^{1}**2d**^{nit})	–532.2626
Electronic energy (^{3}**2d**^{nit})	–532.3059
Main electronic configuration (^{1}**2d**^{nit})	2 2 2 1 1 0 0 0
Weight of the main electronic configuration (^{1}**2d**^{nit})	0.88930
Singlet-triplet gap	–27.2
Occupation of the HONO (η^{HONO}) - ^{1}**2d**^{nit}	1.0050
Occupation of the LUNO (η^{LUNO}) - ^{1}**2d**^{nit}	1.0000
HONO-LUNO Orbital Overlap (T) - ^{1}**2d**^{nit}	0.0025
Biradical character (y_0) - ^{1}**2d**^{nit}	0.99
**NEVPT2(8,8)**	
Electronic energy (^{1}**2d**^{nit})	–533.7889
Electronic energy (^{3}**2d**^{nit})	–533.8029
Singlet-triplet gap	–8.8

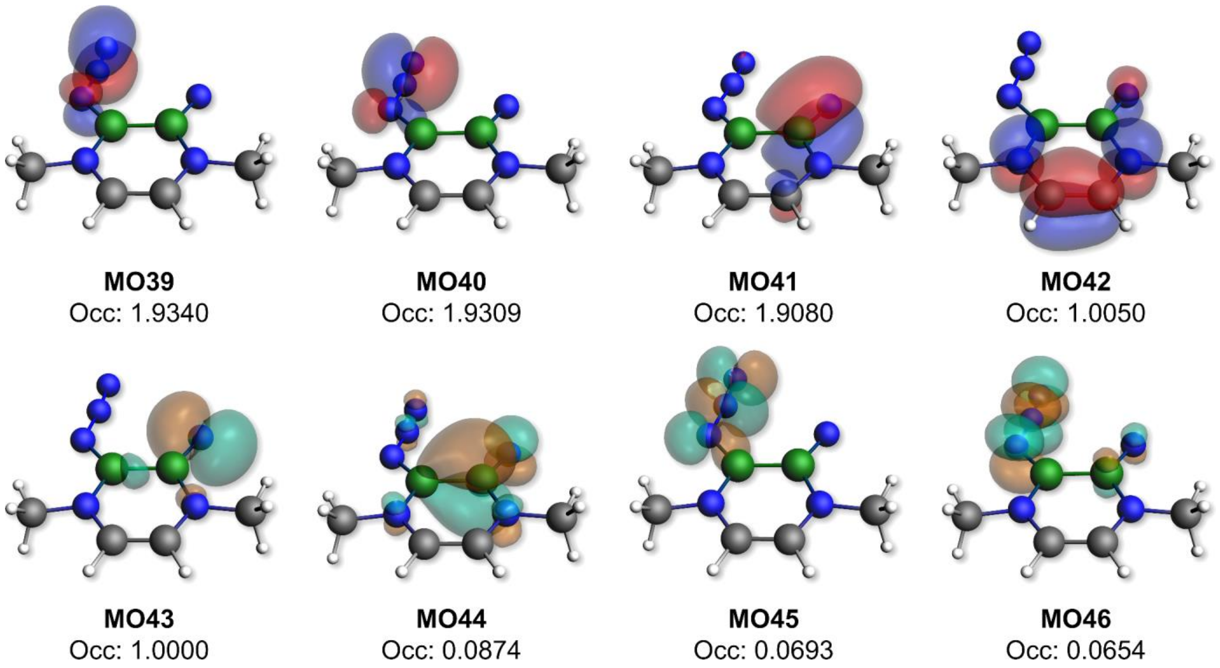

Figure S35: Active space orbitals of the minimal model of **^{1}2d^{nit}** at the CASSCF(8,8)/cc-pVDZ level of theory.

Reaction Mechanism

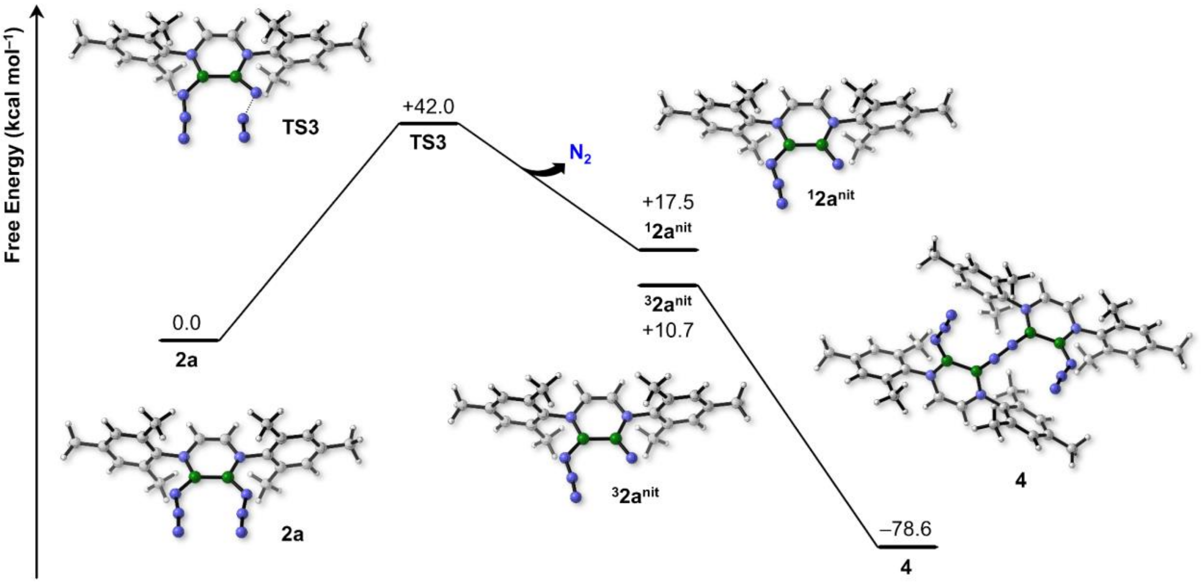

Figure S36: Computed nitrene reaction pathway, illustrating the formation of the hypothetical azo-bridged bis(diazadiborinine) species **4**.

Table S19: Harmonic frequencies (cm^{-1}), reduced masses (amu), force constants (mDyne Å^{-1}) and IR intensities (km mol^{-1}) for the imaginary vibrational frequency mode of **TS1**.

Vibrational Mode	Frequencies	Reduced Masses	Force Constants	IR Intensities
		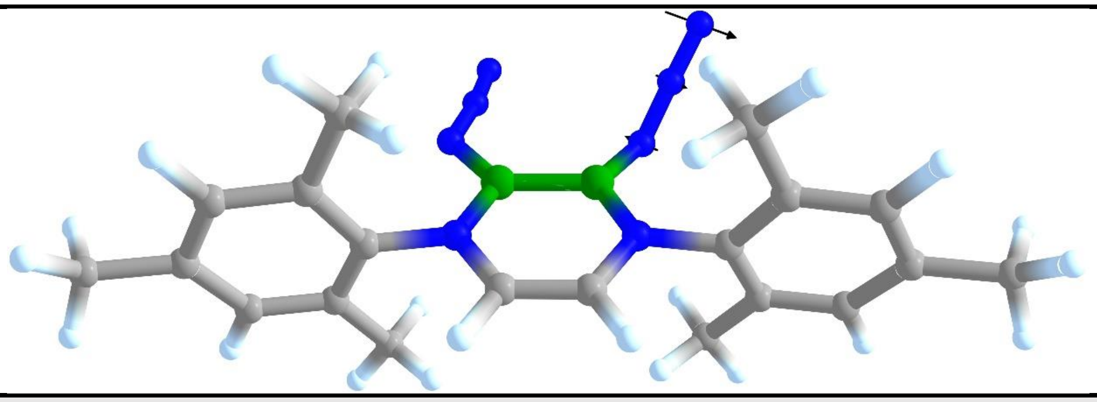		
		**TS1**		
Im.	–74.1897	12.7719	0.0414	0.3312

Table S20: Harmonic frequencies (cm^{-1}), reduced masses (amu), force constants (mDyne Å^{-1}) and IR intensities (km mol^{-1}) for the imaginary vibrational frequency mode of **TS2**.

Vibrational Mode	Frequencies	Reduced Masses	Force Constants	IR Intensities
		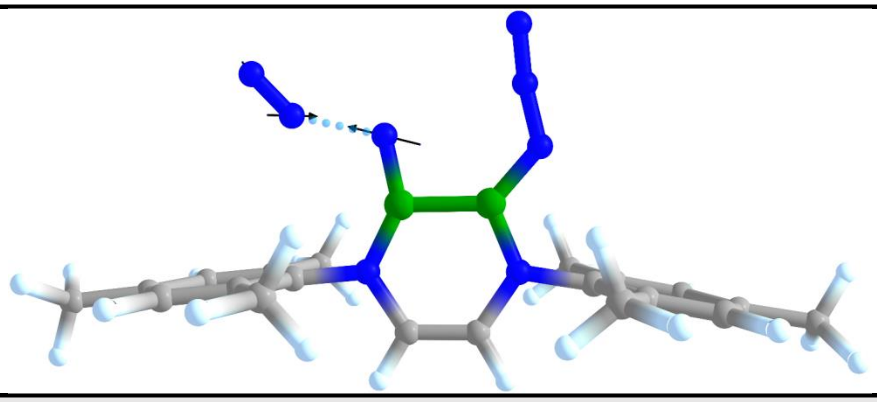		
		**TS2**		
Im.	–478.7781	13.7882	1.8622	63.5296

Table S21: Harmonic frequencies (cm^{-1}), reduced masses (amu), force constants (mDyne $Å^{-1}$) and IR intensities (km mol^{-1}) for the imaginary vibrational frequency mode of **TS3**.

Vibrational Mode	Frequencies	Reduced Masses	Force Constants	IR Intensities
		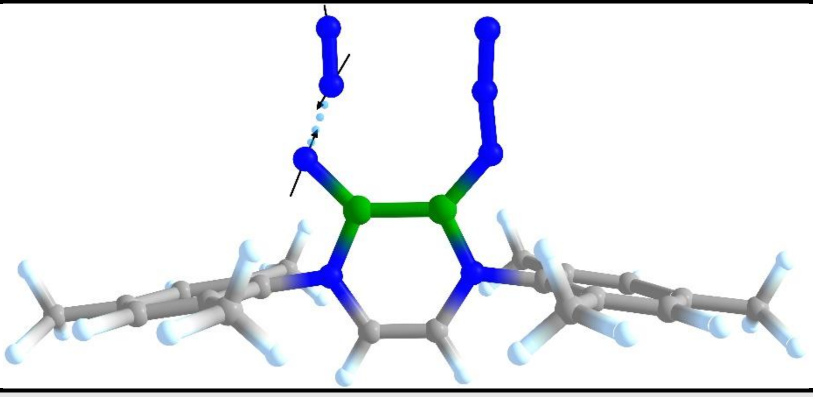		
		**TS3**		
Im.	–534.1159	13.8327	2.3250	2.7153

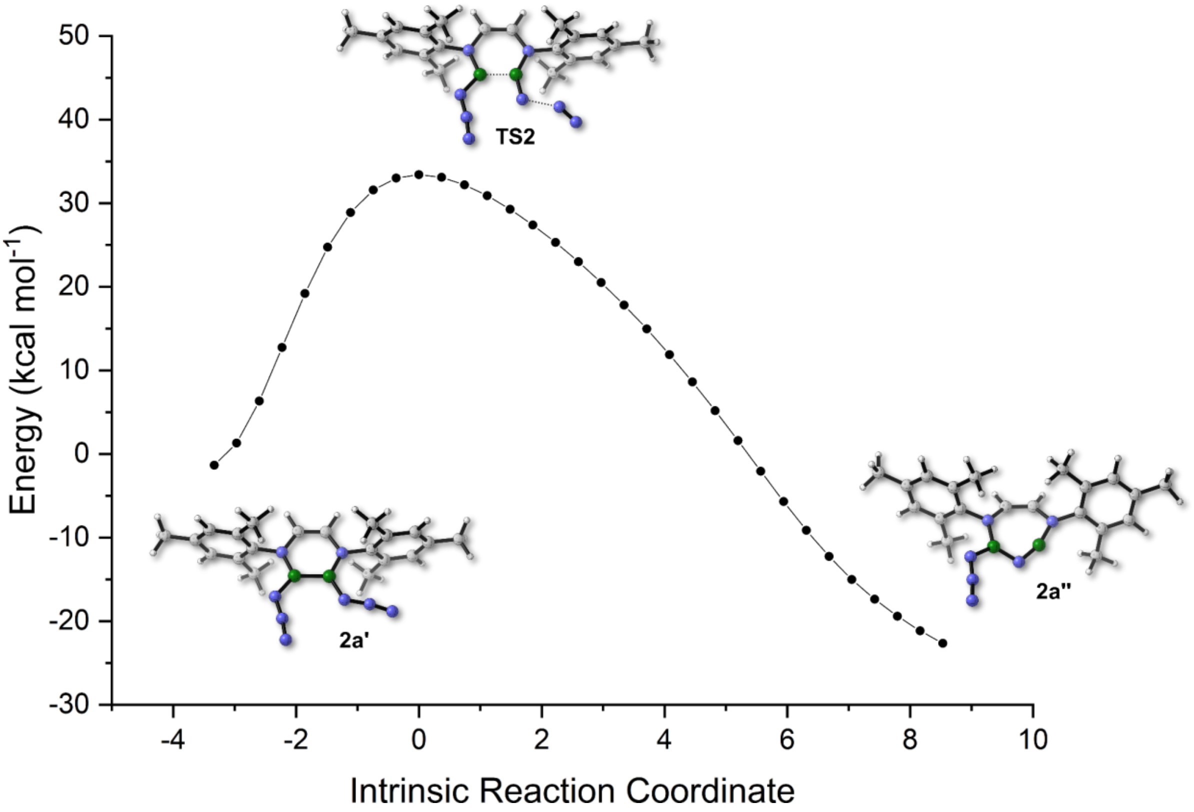

Figure S37: Intrinsic reaction coordinate (IRC) plot showing the connectivity of **2a'**, **TS2** and **2a''**.

Cartesian Coordinates

1 (B3LYP/6-31+G*)			
B	0.753439000	0.414620000	-0.108257000
B	-0.753494000	-0.414638000	-0.108265000
N	1.882737000	-0.010104000	-0.833305000
N	-1.882799000	0.010116000	-0.833286000
C	-3.183149000	-0.655633000	-0.796940000
H	-3.472812000	-0.988799000	-1.803740000
H	-3.144155000	-1.521279000	-0.135347000
H	-3.957160000	0.035040000	-0.433845000
C	-1.875767000	1.179192000	-1.704489000
H	-2.607262000	1.925246000	-1.362423000
H	-0.887210000	1.643831000	-1.718838000
H	-2.139588000	0.897800000	-2.734287000
C	1.875691000	-1.179136000	-1.704569000
H	2.607182000	-1.925212000	-1.362547000
H	0.887130000	-1.643765000	-1.718936000
H	2.139509000	-0.897692000	-2.734354000
C	3.183094000	0.655633000	-0.796932000
H	3.144110000	1.521245000	-0.135294000
H	3.957101000	-0.035067000	-0.433878000
H	3.472753000	0.988848000	-1.803716000
N	0.953297000	1.630808000	0.711018000
N	0.073348000	2.120857000	1.414967000
N	-0.660893000	2.664973000	2.098817000
N	-0.953332000	-1.630883000	0.710929000
N	-0.073370000	-2.120983000	1.414833000
N	0.661220000	-2.664847000	2.098512000

1b (B3LYP/6-31+G*)			
B	0.576546000	-0.112080000	0.060226000
B	-1.131441000	-0.068468000	-0.129252000
N	1.249404000	0.564230000	1.102045000

N	-1.995053000	-1.129550000	0.201595000
C	-3.440284000	-1.113297000	-0.008490000
H	-3.969473000	-1.275279000	0.941575000
H	-3.749239000	-0.154821000	-0.426372000
H	-3.737344000	-1.914549000	-0.700313000
C	-1.524361000	-2.387577000	0.766150000
H	-1.771946000	-3.229172000	0.103122000
H	-0.441197000	-2.366849000	0.902622000
H	-1.995718000	-2.578210000	1.741548000
C	0.520036000	1.267961000	2.156589000
H	0.769884000	2.338420000	2.154189000
H	-0.557573000	1.161177000	2.019470000
H	0.783394000	0.862023000	3.143853000
C	2.693743000	0.680301000	1.290492000
H	3.249619000	0.216329000	0.477692000
H	2.984825000	1.738806000	1.336462000
H	3.002050000	0.205916000	2.233110000
N	1.253526000	-0.874002000	-1.002634000
N	2.422055000	-1.189883000	-1.188491000
N	3.459632000	-1.566797000	-1.486911000
N	-1.798113000	1.126121000	-0.699330000
N	-1.187908000	2.130616000	-1.055085000
N	-0.730346000	3.112782000	-1.415515000

1c (B3LYP/6-31+G*)

B	-0.858286000	0.027381000	-0.085665000
B	0.858278000	-0.027402000	-0.085640000
N	-1.614280000	1.014569000	-0.752224000
N	1.614291000	-1.014575000	-0.752199000
C	3.071440000	-1.082577000	-0.834446000
H	3.378833000	-1.233318000	-1.878244000
H	3.537559000	-0.162891000	-0.481956000
H	3.465390000	-1.922108000	-0.243778000
C	0.982684000	-2.128627000	-1.457493000

H	1.322841000	-3.088365000	-1.043349000
H	-0.102401000	-2.083747000	-1.358865000
H	1.244143000	-2.109179000	-2.525479000
C	-0.982649000	2.128604000	-1.457522000
H	-1.322779000	3.088352000	-1.043379000
H	0.102436000	2.083696000	-1.358899000
H	-1.244113000	2.109160000	-2.525508000
C	-3.071428000	1.082613000	-0.834453000
H	-3.537568000	0.162937000	-0.481964000
H	-3.465348000	1.922150000	-0.243775000
H	-3.378828000	1.233370000	-1.878247000
N	-1.483061000	-1.064556000	0.694843000
N	-2.561218000	-1.106283000	1.274730000
N	-3.516035000	-1.263565000	1.884379000
N	1.483037000	1.064610000	0.694776000
N	2.561213000	1.106426000	1.274625000
N	3.515993000	1.263370000	1.884419000

2a (B3LYP/6-31+G*)

C	-2.832588202	-0.609518045	-2.542222184
C	-3.561800256	-0.745774053	-1.225747089
H	-1.991754145	-1.308802094	-2.620100189
H	-3.509974250	-0.799948056	-3.380593244
H	-2.421869175	0.400232029	-2.664826192
C	-2.883882210	-0.637363045	0.000062000
C	-4.943396358	-0.972262069	-1.200030086
C	-5.653800426	-1.085629077	0.000097000
C	-4.943362353	-0.972188069	1.200243085
C	-3.561802256	-0.745713055	1.225923088
H	-5.476311414	-1.061344074	-2.145139155
H	-5.476271408	-1.061207076	2.145368156
C	-2.832531204	-0.609446045	2.542364184
H	-2.421066174	0.400041029	2.664586194
H	-1.992209142	-1.309320095	2.620544190

H	-3.510078255	-0.799062059	3.380789241
C	-7.151124492	-1.296395094	0.000210000
H	-7.477732531	-1.854477131	0.884751064
H	-7.478831527	-1.847402132	-0.888324064
H	-7.685014541	-0.336312024	0.004429000
N	-1.447351106	-0.430179031	0.000053000
B	-0.847176061	0.873022062	-0.000119000
B	0.845604061	0.874652061	-0.000135000
N	1.817383129	1.962983143	-0.000739000
N	1.614762118	3.172813226	-0.002340000
N	1.583892114	4.314110310	-0.003827000
N	1.448396104	-0.427336031	0.000295000
C	-0.674454047	-1.592843115	0.000392000
C	0.677802051	-1.591515113	0.000517000
H	-1.219196087	-2.529722181	0.000566000
H	1.224399090	-2.527306180	0.000873000
C	2.885272205	-0.632212044	0.000229000
C	3.564443258	-0.733267055	1.226106088
C	3.563903254	-0.735748055	-1.225555088
C	4.946961357	-0.953514071	-1.199882085
C	5.657222404	-1.067896077	0.000159000
C	4.947332357	-0.951028070	1.200368087
H	5.481516401	-1.031902076	-2.144990154
H	5.482262382	-1.027378076	2.145468155
C	2.836176204	-0.590475041	2.542410185
H	3.513956251	-0.777463055	3.381238242
H	1.995314142	-1.289305092	2.623742188
H	2.425761173	0.419911030	2.660756189
C	2.835344205	-0.595129043	-2.541939185
H	3.512929255	-0.783558055	-3.380602245
H	2.424997173	0.415115030	-2.661857190
H	1.994371143	-1.293961095	-2.621995189
C	7.145308516	-1.336355099	0.000649000
H	7.353239509	-2.414899175	0.024353002

H	7.632536562	-0.891574064	0.875465062
H	7.625848555	-0.931308068	-0.896741062
N	-1.821054132	1.959441142	-0.000197000
N	-1.621191119	3.169695229	0.000641000
N	-1.592709117	4.311071308	0.001455000

2a' (B3LYP/6-31+G*)

C	-2.980212000	-0.473255000	-2.541766000
C	-3.713020000	-0.592635000	-1.225608000
H	-2.162407000	-1.199032000	-2.623429000
H	-3.663556000	-0.637481000	-3.380908000
H	-2.536788000	0.523166000	-2.658788000
C	-3.031751000	-0.501563000	0.000043000
C	-5.099780000	-0.784835000	-1.200164000
C	-5.812705000	-0.880769000	0.000015000
C	-5.099835000	-0.784617000	1.200203000
C	-3.713071000	-0.592411000	1.225674000
H	-5.634864000	-0.859979000	-2.145297000
H	-5.634961000	-0.859590000	2.145325000
C	-2.980327000	-0.472790000	2.541846000
H	-2.536940000	0.523665000	2.658720000
H	-2.162505000	-1.198529000	2.623667000
H	-3.663705000	-0.636897000	3.380984000
C	-7.314944000	-1.053573000	-0.000019000
H	-7.656185000	-1.598955000	0.886931000
H	-7.655984000	-1.600403000	-0.886158000
H	-7.824357000	-0.080254000	-0.000881000
N	-1.593048000	-0.326311000	0.000056000
B	-0.948949000	0.952736000	-0.000028000
B	0.729655000	0.925120000	-0.000066000
N	1.485309000	2.172786000	-0.000257000
N	2.699285000	2.373253000	-0.000324000
N	3.783176000	2.729406000	-0.000667000
N	1.327683000	-0.376911000	0.000072000

C	-0.832244000	-1.499583000	0.000160000
C	0.519990000	-1.525008000	0.000177000
H	-1.389379000	-2.429601000	0.000235000
H	1.044964000	-2.473318000	0.000272000
C	2.755636000	-0.618904000	0.000095000
C	3.431712000	-0.744460000	1.227080000
C	3.431696000	-0.744771000	-1.226867000
C	4.809419000	-0.991583000	-1.200518000
C	5.518066000	-1.112096000	0.000140000
C	4.809434000	-0.991282000	1.200776000
H	5.341571000	-1.089431000	-2.145004000
H	5.341596000	-1.088896000	2.145281000
C	2.701588000	-0.613100000	2.544421000
H	3.378906000	-0.809295000	3.381283000
H	1.860645000	-1.312775000	2.615772000
H	2.287772000	0.393997000	2.679090000
C	2.701555000	-0.613742000	-2.544230000
H	3.378904000	-0.809968000	-3.381060000
H	2.287572000	0.393264000	-2.679062000
H	1.860724000	-1.313566000	-2.615470000
C	7.012748000	-1.337443000	0.000160000
H	7.552929000	-0.381114000	0.000058000
H	7.334879000	-1.894512000	-0.886543000
H	7.334883000	-1.894324000	0.886980000
N	-1.842151000	2.107311000	-0.000198000
N	-1.463895000	3.278822000	-0.000333000
N	-1.263639000	4.400938000	0.000045000

2a'' (B3LYP/6-31+G*)

C	2.291515165	-2.116562155	1.021444073
C	3.411120247	-1.271951089	0.460519033
H	1.729341127	-2.631923189	0.233570017
H	2.689043195	-2.876076205	1.701905122

H	1.569959115	-1.504984107	1.574672115
C	3.157660225	-0.072649005	-0.234118017
C	4.743632344	-1.653996118	0.655488048
C	5.816123431	-0.880144063	0.198315014
C	5.526684378	0.315513023	-0.467708034
C	4.212167303	0.736712052	-0.701217048
H	4.946176354	-2.576580186	1.196724088
H	6.345185478	0.938070070	-0.824344058
C	3.949927283	2.032868148	-1.435123106
H	3.553607253	2.813602201	-0.771319053
H	3.223945232	1.901530138	-2.245536160
H	4.876330349	2.421797175	-1.869059134
C	7.244073523	-1.333251098	0.400909029
H	7.930829597	-0.481715035	0.458390033
H	7.579000546	-1.968112140	-0.430755031
H	7.352032508	-1.918440140	1.320882094
N	1.805313130	0.345843025	-0.472990034
B	1.127491082	1.474432106	-0.062743004
N	0.143101010	2.275719166	0.170796012
B	-1.062961076	1.524410109	0.354009026
N	-2.169019157	1.996992142	1.193699088
N	-2.080817152	3.081893224	1.770827125
N	-2.110212149	4.060863293	2.350728167
N	-1.319448097	0.248829018	-0.316046023
C	0.906380063	-0.425978031	-1.289616095
C	-0.442889032	-0.429141031	-1.192861083
H	1.381418098	-0.984058070	-2.091703148
H	-0.958356069	-1.054605076	-1.917420140
C	-2.646993189	-0.338435024	-0.269526019
C	-3.627608262	0.093393007	-1.180847088
C	-2.927126210	-1.338616098	0.677182051
C	-4.207256305	-1.905684137	0.691235051
C	-5.203547376	-1.506849108	-0.206521015
C	-4.893226354	-0.503902036	-1.131365083

H	-4.430757316	-2.676297194	1.427275100
H	-5.657056401	-0.170594012	-1.832050134
C	-3.342779238	1.192378087	-2.178511157
H	-4.189841304	1.325248097	-2.859068205
H	-2.452567175	0.982658069	-2.782915201
H	-3.165067230	2.150550156	-1.674424122
C	-1.884898134	-1.780970130	1.677025119
H	-2.272867165	-2.584591184	2.311256164
H	-1.584243111	-0.951517067	2.328861166
H	-0.977318072	-2.146926155	1.183050084
C	-6.568364448	-2.157987155	-0.193542014
H	-6.595567492	-3.035972217	-0.853587060
H	-7.345345507	-1.466824104	-0.538764041
H	-6.840536500	-2.497917179	0.811951060

**3a (B3LYP/6-31+G*)**			
B	-2.483468211	-0.160580084	-0.074675993
B	0.024326979	-0.976455116	0.265868032
N	-1.094527113	-0.016216059	0.219824029
N	-0.046657011	-2.381070217	0.421096043
C	-1.281988095	-3.056004280	0.593832056
H	-1.155900074	-4.077843352	0.938047082
C	-2.558203189	-2.657393262	0.423795043
H	-3.303172236	-3.415521329	0.645496060
N	-3.142749243	-1.437009184	-0.003614988
N	-3.170121275	1.060350999	-0.493151023
N	-4.338156357	1.281880000	-0.785177046
N	-5.374528456	1.655646014	-1.084924064
C	-4.542917345	-1.578599209	-0.341646012
C	-4.904702367	-1.921315234	-1.657566107
C	-6.262920482	-2.083198265	-1.955636130
H	-6.548952480	-2.345000284	-2.972980201
C	-7.258202556	-1.918492259	-0.985823056

C	-6.863422516	-1.593518234	0.316646036
H	-7.622196544	-1.468578231	1.087318089
C	-5.517670426	-1.418975209	0.660876062
C	-3.856967287	-2.107716237	-2.730938184
H	-4.315892319	-2.450508268	-3.663773252
H	-3.098740228	-2.840478282	-2.431805160
H	-3.325890260	-1.171426166	-2.944332201
C	-8.720584624	-2.060305290	-1.341745081
H	-9.141757696	-1.097601220	-1.661924109
H	-9.310522693	-2.406001318	-0.485562022
H	-8.866840643	-2.770078340	-2.163594143
C	-5.131999390	-1.056690177	2.077299161
H	-4.379008327	-1.741507218	2.483705192
H	-6.007476464	-1.082806186	2.733870207
H	-4.704098369	-0.047585099	2.134039165
C	1.118796081	-3.224186267	0.571065052
C	1.741152130	-3.326992267	1.826109145
C	2.851203218	-4.173729317	1.952141153
H	3.338554250	-4.257982315	2.922159225
C	3.342540261	-4.915347366	0.873520076
C	2.687027210	-4.800918363	-0.359033013
H	3.051227244	-5.373430413	-1.210785074
C	1.579892121	-3.964286312	-0.532850025
C	1.238449084	-2.546463217	3.019187228
H	1.760027126	-2.856155232	3.930669295
H	0.163278007	-2.691174239	3.176185244
H	1.399307083	-1.468536135	2.891530223
C	4.558344359	-5.801735419	1.022560086
H	5.448506430	-5.326582352	0.588683056
H	4.421150358	-6.761235479	0.509964049
H	4.775842373	-6.009297416	2.075878161
C	0.914304074	-3.842824311	-1.883009125
H	1.006450068	-2.822755237	-2.276214153
H	-0.157155001	-4.069513340	-1.832393120

H	1.373669112	-4.525846356	-2.604830176
B	-0.024309045	0.976482025	0.265759032
N	1.094544046	0.016237967	0.219775029
B	2.483470142	0.160571992	-0.074808993
N	3.142751180	1.437011089	-0.003933988
C	2.558226123	2.657442174	0.423371043
H	3.303205167	3.415594234	0.644952060
C	1.282019025	3.056070189	0.593424056
H	1.155945006	4.077947261	0.937531082
N	0.046680945	2.381116123	0.420814043
N	3.170109204	-1.060401091	-0.493186023
N	4.338132295	-1.281948094	-0.785250046
N	5.374516391	-1.655729110	-1.084934066
C	4.542898278	1.578564114	-0.342068012
C	5.517715353	1.419047114	0.660411058
C	6.863445430	1.593550143	0.316077035
H	7.622267507	1.468693142	1.086715091
C	7.258144452	1.918379168	-0.986453058
C	6.262801418	2.082978169	-1.956222126
H	6.548770390	2.344665193	-2.973613203
C	4.904602300	1.921131144	-1.658048108
C	5.132132324	1.056915083	2.076897161
H	4.704241305	0.047813008	2.133773168
H	4.379163265	1.741770124	2.483276194
H	6.007649399	1.083106097	2.733411209
C	8.720504560	2.060150193	-1.342481082
H	9.141667630	1.097400132	-1.662535108
H	9.310489619	2.405983226	-0.486386022
H	8.866706550	2.769797247	-2.164448145
C	3.856799221	2.107415146	-2.731375184
H	4.315667250	2.450094177	-3.664280251
H	3.098598159	2.840218192	-2.432279160
H	3.325701196	1.171105072	-2.944626202
C	-1.118768146	3.224247175	0.570728056

C	-1.579912187	3.964207221	-0.533260026
C	-2.687042275	4.800859270	-0.359501013
H	-3.051279310	5.373263299	-1.211310076
C	-3.342504325	4.915439273	0.873065074
C	-2.851121280	4.173957223	1.951758156
H	-3.338432317	4.258329223	2.921785224
C	-1.741073193	3.327207176	1.825786146
C	-0.914380138	3.842579223	-1.883431123
H	0.157081935	4.069265246	-1.832886117
H	-1.373769179	4.525518267	-2.605315173
H	-1.006551136	2.822464147	-2.276510153
C	-4.558305422	5.801843318	1.022045086
H	-4.421131424	6.761283370	0.509330049
H	-4.775763441	6.009529354	2.075347163
H	-5.448481506	5.326637280	0.588257052
C	-1.238318150	2.546827122	3.018939230
H	-1.399161151	1.468882043	2.891416223
H	-1.759872188	2.856619141	3.930401294
H	-0.163145073	2.691577144	3.175886243

^{1}2a^{nit} (UB3LYP/6-31+G*)

C	-2.741821000	-0.432534000	-2.543080000
C	-3.469416000	-0.581864000	-1.227039000
H	-1.923122000	-1.155195000	-2.645754000
H	-3.428059000	-0.582169000	-3.382237000
H	-2.301351000	0.566812000	-2.642512000
C	-2.795194000	-0.466771000	0.000036000
C	-4.847301000	-0.828637000	-1.200415000
C	-5.555477000	-0.952818000	0.000074000
C	-4.847323000	-0.828330000	1.200543000
C	-3.469437000	-0.581549000	1.227129000
H	-5.379369000	-0.923031000	-2.145322000
H	-5.379407000	-0.922483000	2.145465000
C	-2.741867000	-0.431882000	2.543144000

H	-2.301442000	0.567507000	2.642349000
H	-1.923138000	-1.154482000	2.645999000
H	-3.428110000	-0.581349000	3.382327000
C	-7.049540000	-1.184374000	0.000089000
H	-7.368957000	-1.742984000	0.886779000
H	-7.368925000	-1.743311000	-0.886407000
H	-7.595820000	-0.231382000	-0.000097000
N	-1.362799000	-0.226288000	0.000017000
B	-0.769931000	1.103826000	-0.000132000
B	0.916525000	1.166788000	-0.000116000
N	1.666156000	2.334039000	-0.000241000
N	1.564956000	-0.169339000	0.000028000
C	-0.554489000	-1.339347000	0.000146000
C	0.824246000	-1.311630000	0.000149000
H	-1.058734000	-2.299944000	0.000250000
H	1.366004000	-2.252254000	0.000253000
C	3.008680000	-0.318005000	0.000052000
C	3.687985000	-0.384538000	1.227971000
C	3.688010000	-0.384714000	-1.227845000
C	5.079225000	-0.538824000	-1.200123000
C	5.794021000	-0.615653000	0.000101000
C	5.079202000	-0.538651000	1.200300000
H	5.616528000	-0.593224000	-2.145214000
H	5.616486000	-0.592913000	2.145410000
C	2.954118000	-0.272259000	2.544104000
H	3.649252000	-0.379584000	3.382483000
H	2.178348000	-1.040422000	2.651019000
H	2.459512000	0.701879000	2.635459000
C	2.954169000	-0.272624000	-2.544009000
H	3.649309000	-0.380144000	-3.382358000
H	2.459628000	0.701529000	-2.635544000
H	2.178352000	-1.040756000	-2.650796000
C	7.300568000	-0.743069000	0.000124000
H	7.658124000	-1.278419000	-0.886449000

H	7.658119000	-1.278168000	0.886849000
H	7.778988000	0.245730000	-0.000016000
N	-1.727459000	2.190318000	-0.000245000
N	-1.389055000	3.379087000	-0.000367000
N	-1.228275000	4.504201000	-0.000535000

^{3}2a^{nit} (UB3LYP/6-31+G*)

C	-2.737597000	-0.431106000	-2.542791000
C	-3.464795000	-0.581018000	-1.226654000
H	-1.907695000	-1.141693000	-2.638047000
H	-3.420254000	-0.597217000	-3.381860000
H	-2.312233000	0.574168000	-2.648945000
C	-2.790017000	-0.464374000	0.000042000
C	-4.842450000	-0.829364000	-1.200304000
C	-5.550631000	-0.954113000	0.000077000
C	-4.842500000	-0.828907000	1.200439000
C	-3.464846000	-0.580550000	1.226754000
H	-5.374276000	-0.925061000	-2.145264000
H	-5.374365000	-0.924250000	2.145413000
C	-2.737710000	-0.430122000	2.542866000
H	-2.312684000	0.575320000	2.648803000
H	-1.907575000	-1.140411000	2.638281000
H	-3.420322000	-0.596269000	3.381964000
C	-7.044412000	-1.187734000	0.000088000
H	-7.363071000	-1.746816000	0.886793000
H	-7.363015000	-1.747268000	-0.886352000
H	-7.592322000	-0.235645000	-0.000172000
N	-1.357924000	-0.224650000	0.000025000
B	-0.771634000	1.098829000	-0.000107000
B	0.914073000	1.153543000	-0.000112000
N	1.695722000	2.335383000	-0.000226000
N	1.557161000	-0.157435000	0.000020000
C	-0.550446000	-1.346746000	0.000135000
C	0.817756000	-1.317939000	0.000135000

H	-1.060597000	-2.303945000	0.000232000
H	1.371083000	-2.250902000	0.000226000
C	3.001796000	-0.307850000	0.000041000
C	3.681496000	-0.380055000	1.227602000
C	3.681531000	-0.380094000	-1.227497000
C	5.072158000	-0.540116000	-1.200076000
C	5.786781000	-0.619531000	0.000086000
C	5.072124000	-0.540077000	1.200226000
H	5.609078000	-0.599090000	-2.145119000
H	5.609018000	-0.599016000	2.145286000
C	2.948986000	-0.268598000	2.544670000
H	3.637881000	-0.417481000	3.381877000
H	2.145323000	-1.009386000	2.633502000
H	2.489321000	0.720582000	2.657566000
C	2.949062000	-0.268674000	-2.544591000
H	3.637940000	-0.417804000	-3.381768000
H	2.489596000	0.720584000	-2.657626000
H	2.145253000	-1.009312000	-2.633332000
C	7.292614000	-0.754738000	0.000108000
H	7.647366000	-1.291926000	-0.886472000
H	7.647354000	-1.291769000	0.886788000
H	7.776332000	0.231487000	0.000024000
N	-1.727434000	2.192774000	-0.000228000
N	-1.392326000	3.380409000	-0.000363000
N	-1.229875000	4.506589000	-0.000491000

^{1}2d^{nit} (UB3LYP/6-31+G*)

C	0.793365000	2.850546000	0.000126000
N	0.027380000	1.594020000	0.000105000
B	0.634596000	0.274041000	-0.000028000
B	-0.430592000	-1.034966000	-0.000035000
N	-0.060360000	-2.372486000	-0.000148000
N	-1.859556000	-0.642825000	0.000106000
C	-1.339030000	1.717069000	0.000219000

C	-2.228407000	0.663320000	0.000221000
H	-1.735881000	2.727631000	0.000316000
H	-3.292071000	0.884220000	0.000319000
C	-2.930380000	-1.648883000	0.000125000
N	2.085155000	0.267516000	-0.000146000
N	2.744940000	-0.777796000	-0.000271000
N	3.470251000	-1.652254000	-0.000396000
H	1.857240000	2.617339000	0.000032000
H	0.555924000	3.441511000	0.892249000
H	0.555791000	3.441623000	-0.891888000
H	-2.474332000	-2.639079000	0.000022000
H	-3.557401000	-1.537020000	-0.892057000
H	-3.557257000	-1.537139000	0.892424000

^{3}2d^{nit} (UB3LYP/6-31+G*)

C	0.782411000	2.849315000	0.000125000
N	0.021329000	1.590512000	0.000103000
B	0.631600000	0.280584000	-0.000033000
B	-0.434862000	-1.025780000	-0.000033000
N	-0.064209000	-2.392444000	-0.000153000
N	-1.844361000	-0.650574000	0.000105000
C	-1.350843000	1.714951000	0.000222000
C	-2.229628000	0.665189000	0.000222000
H	-1.745151000	2.726298000	0.000320000
H	-3.295645000	0.870295000	0.000321000
C	-2.920591000	-1.652131000	0.000125000
N	2.086592000	0.272735000	-0.000150000
N	2.751189000	-0.767211000	-0.000271000
N	3.479073000	-1.641139000	-0.000385000
H	1.847632000	2.621795000	0.000028000
H	0.542476000	3.439863000	0.892057000
H	0.542337000	3.439978000	-0.891693000
H	-2.475992000	-2.647509000	0.000023000
H	-3.547435000	-1.535856000	-0.891652000

H	-3.547291000	-1.535975000	0.892019000

4 (B3LYP/6-31+G*)

C	4.751936000	-0.420604000	-2.481343000
C	5.757144000	0.146920000	-1.506299000
H	4.122975000	0.362256000	-2.921409000
H	5.259313000	-0.946826000	-3.296462000
H	4.078624000	-1.131362000	-1.986787000
C	5.351209000	0.949087000	-0.427295000
C	7.123258000	-0.125963000	-1.654816000
C	8.078637000	0.371346000	-0.762753000
C	7.636776000	1.167584000	0.300547000
C	6.282807000	1.467424000	0.489960000
H	7.445822000	-0.743070000	-2.491734000
H	8.364479000	1.567464000	1.004918000
C	5.842428000	2.314853000	1.660819000
H	5.207630000	1.741659000	2.347350000
H	5.259673000	3.186258000	1.339843000
H	6.708582000	2.675120000	2.224869000
C	9.545212000	0.040140000	-0.925005000
H	10.180642000	0.897124000	-0.673246000
H	9.776393000	-0.261984000	-1.952269000
H	9.839705000	-0.787259000	-0.265110000
N	3.946205000	1.264741000	-0.271041000
B	3.043303000	0.478695000	0.509683000
B	1.449625000	1.037125000	0.557011000
N	0.486401000	0.370294000	1.375165000
N	1.220702000	2.313815000	-0.068846000
C	3.519943000	2.464776000	-0.860891000
C	2.267036000	2.956703000	-0.746212000
H	4.271147000	3.016433000	-1.414127000
H	2.018001000	3.907589000	-1.203482000
C	-0.040224000	3.025017000	-0.067446000
C	-0.397457000	3.790314000	1.057004000

C	-0.855735000	2.988930000	-1.215035000
C	-2.041219000	3.733058000	-1.211829000
C	-2.431629000	4.500769000	-0.108129000
C	-1.594763000	4.516280000	1.011829000
H	-2.675407000	3.711485000	-2.096671000
H	-1.878716000	5.106695000	1.880632000
C	0.477010000	3.836671000	2.287876000
H	0.110838000	4.590377000	2.991635000
H	1.516730000	4.076698000	2.036780000
H	0.484145000	2.870612000	2.807160000
C	-0.472897000	2.173589000	-2.428553000
H	-1.259147000	2.219945000	-3.188524000
H	-0.313856000	1.120406000	-2.169907000
H	0.458960000	2.528262000	-2.885573000
C	-3.734954000	5.266910000	-0.117627000
H	-3.969403000	5.648407000	-1.118029000
H	-3.704039000	6.117829000	0.571552000
H	-4.571236000	4.625260000	0.191203000
N	3.638768000	-0.663807000	1.197291000
N	3.098393000	-1.291356000	2.105762000
N	2.733730000	-1.938217000	2.971237000
C	-4.752019000	0.420778000	-2.480678000
C	-5.757269000	-0.147398000	-1.506056000
H	-4.122192000	-0.361645000	-2.920263000
H	-5.259412000	0.946432000	-3.296156000
H	-4.079568000	1.132213000	-1.985917000
C	-5.351402000	-0.949923000	-0.427328000
C	-7.123431000	0.125285000	-1.654709000
C	-8.078882000	-0.372611000	-0.763086000
C	-7.637067000	-1.169209000	0.299992000
C	-6.283091000	-1.468829000	0.489548000
H	-7.445950000	0.742708000	-2.491407000
H	-8.364822000	-1.569521000	1.004068000
C	-5.842741000	-2.316499000	1.660246000

H	-5.208428000	-1.743240000	2.347179000
H	-5.259488000	-3.187541000	1.339203000
H	-6.708928000	-2.677327000	2.223886000
C	-9.545548000	-0.041910000	-0.925564000
H	-10.180498000	-0.901016000	-0.679817000
H	-9.775485000	0.265974000	-1.951384000
H	-9.841897000	0.781246000	-0.261222000
N	-3.946353000	-1.265325000	-0.270856000
B	-3.043779000	-0.479015000	0.510035000
B	-1.449942000	-1.036881000	0.557357000
N	-0.486596000	-0.370041000	1.375636000
N	-1.220604000	-2.313349000	-0.068718000
C	-3.519754000	-2.465185000	-0.860710000
C	-2.266661000	-2.956672000	-0.746074000
H	-4.270801000	-3.017101000	-1.413901000
H	-2.017282000	-3.907489000	-1.203290000
C	0.040601000	-3.024139000	-0.067382000
C	0.398010000	-3.789572000	1.056861000
C	0.856102000	-2.987551000	-1.214994000
C	2.041749000	-3.731361000	-1.211973000
C	2.432373000	-4.499245000	-0.108435000
C	1.595530000	-4.515236000	1.011498000
H	2.675958000	-3.709424000	-2.096793000
H	1.879619000	-5.105751000	1.880187000
C	-0.476417000	-3.836534000	2.287741000
H	-0.109807000	-4.590103000	2.991414000
H	-1.516001000	-4.077166000	2.036630000
H	-0.484112000	-2.870529000	2.807105000
C	0.473011000	-2.172029000	-2.428309000
H	1.259393000	-2.217747000	-3.188183000
H	0.313394000	-1.119011000	-2.169359000
H	-0.458595000	-2.527058000	-2.885571000
C	3.735964000	-5.264927000	-0.118122000
H	3.970810000	-5.645648000	-1.118722000

H	3.705173000	-6.116321000	0.570475000
H	4.571941000	-4.623190000	0.191366000
N	-3.639827000	0.663179000	1.197693000
N	-3.099371000	1.291014000	2.105960000
N	-2.734644000	1.938059000	2.971264000

4b (B3LYP/6-31+G*)

C	-5.650551000	-2.120056000	-2.481979000
C	-6.363848000	-1.298795000	-1.433198000
H	-5.181673000	-3.014273000	-2.054661000
H	-6.348713000	-2.443208000	-3.260600000
H	-4.853228000	-1.540904000	-2.963346000
C	-5.691006000	-0.826813000	-0.292909000
C	-7.718052000	-0.976882000	-1.583479000
C	-8.405075000	-0.202551000	-0.641600000
C	-7.701565000	0.249256000	0.479997000
C	-6.346509000	-0.046863000	0.674374000
H	-8.247743000	-1.341107000	-2.462213000
H	-8.218281000	0.849400000	1.227077000
C	-5.614782000	0.472997000	1.890066000
H	-4.822683000	1.176370000	1.605812000
H	-5.135916000	-0.334663000	2.456249000
H	-6.303811000	0.995208000	2.561552000
C	-9.859334000	0.158209000	-0.845918000
H	-10.362000000	0.352971000	0.107879000
H	-10.403621000	-0.643232000	-1.358202000
H	-9.959590000	1.063847000	-1.459663000
N	-4.295610000	-1.166967000	-0.098534000
B	-3.211515000	-0.364304000	-0.580129000
B	-1.665121000	-0.932344000	-0.216328000
N	-0.459814000	-0.213419000	-0.662399000
N	-1.622788000	-2.203626000	0.437891000
C	-4.047834000	-2.373867000	0.560445000
C	-2.809624000	-2.862749000	0.793360000

H	-4.920300000	-2.932120000	0.880380000
H	-2.688699000	-3.814456000	1.297770000
C	-0.411992000	-2.917872000	0.799851000
C	0.188909000	-2.669208000	2.045342000
C	0.087135000	-3.898543000	-0.076148000
C	1.228736000	-4.610030000	0.311430000
C	1.869573000	-4.375434000	1.533535000
C	1.329922000	-3.407055000	2.386896000
H	1.626343000	-5.365931000	-0.363380000
H	1.803653000	-3.221147000	3.349304000
C	-0.380272000	-1.647950000	3.000795000
H	0.194920000	-1.627477000	3.932153000
H	-1.425092000	-1.867590000	3.252904000
H	-0.356534000	-0.645347000	2.559716000
C	-0.589434000	-4.191150000	-1.394825000
H	-0.052690000	-4.975431000	-1.936532000
H	-0.616524000	-3.304145000	-2.036767000
H	-1.625614000	-4.521994000	-1.252152000
C	3.120252000	-5.137521000	1.909760000
H	3.056966000	-6.188382000	1.604183000
H	3.296662000	-5.109148000	2.990561000
H	4.005547000	-4.710171000	1.419881000
N	-3.618175000	0.823675000	-1.325738000
N	-2.871690000	1.556916000	-1.967815000
N	-2.311173000	2.309420000	-2.616344000
C	5.500840000	1.655928000	-2.703618000
C	6.239384000	0.913573000	-1.614376000
H	5.116671000	2.621421000	-2.354118000
H	6.158161000	1.842012000	-3.558872000
H	4.638297000	1.080127000	-3.060570000
C	5.624364000	0.618439000	-0.385286000
C	7.558442000	0.488703000	-1.813977000
C	8.266456000	-0.217219000	-0.834616000
C	7.621346000	-0.491656000	0.375966000

C	6.303121000	-0.087392000	0.622012000
H	8.043163000	0.715778000	-2.762118000
H	8.155812000	-1.034968000	1.153491000
C	5.632898000	-0.415951000	1.935969000
H	4.789527000	-1.101989000	1.790372000
H	5.234168000	0.479788000	2.426576000
H	6.340623000	-0.892449000	2.621994000
C	9.679078000	-0.692933000	-1.089683000
H	10.237707000	-0.810094000	-0.154483000
H	10.229685000	0.007780000	-1.727542000
H	9.682848000	-1.667196000	-1.597398000
N	4.268780000	1.068860000	-0.139347000
B	3.113812000	0.278810000	-0.441644000
B	1.627726000	0.988964000	-0.058884000
N	0.363684000	0.303324000	0.116520000
N	1.719413000	2.357653000	0.378647000
C	4.145129000	2.371427000	0.359668000
C	2.955819000	2.976418000	0.576269000
H	5.071350000	2.900665000	0.550573000
H	2.923744000	3.999873000	0.933037000
C	0.556891000	3.174945000	0.659493000
C	0.055114000	3.231177000	1.971019000
C	-0.023772000	3.922391000	-0.381483000
C	-1.137917000	4.715800000	-0.084280000
C	-1.678411000	4.781882000	1.205743000
C	-1.064423000	4.037008000	2.217914000
H	-1.594480000	5.295119000	-0.884723000
H	-1.461099000	4.085029000	3.230686000
C	0.710117000	2.462448000	3.094578000
H	0.132909000	2.562081000	4.019521000
H	1.726823000	2.825405000	3.292662000
H	0.793568000	1.397771000	2.853283000
C	0.543987000	3.888182000	-1.781358000
H	0.004126000	4.581322000	-2.432827000

H	0.461580000	2.889682000	-2.224672000
H	1.605982000	4.161662000	-1.791645000
C	-2.899326000	5.628339000	1.488689000
H	-2.816671000	6.618703000	1.025217000
H	-3.047039000	5.770501000	2.564666000
H	-3.808477000	5.159722000	1.088792000
N	3.411261000	-1.005634000	-1.073146000
N	2.626698000	-1.690260000	-1.721082000
N	2.025472000	-2.404550000	-2.377440000

TS1 (B3LYP/6-31+G*)

C	-2.955615000	-1.008099000	-2.419576000
C	-3.675017000	-0.864822000	-1.098396000
H	-2.142541000	-1.741851000	-2.367241000
H	-3.648794000	-1.327195000	-3.204169000
H	-2.508553000	-0.056596000	-2.732508000
C	-2.981859000	-0.541189000	0.080149000
C	-5.062167000	-1.040601000	-1.022727000
C	-5.763004000	-0.897464000	0.179781000
C	-5.037529000	-0.574066000	1.331852000
C	-3.650042000	-0.388068000	1.306760000
H	-5.607175000	-1.294976000	-1.930118000
H	-5.563196000	-0.461325000	2.278484000
C	-2.903575000	-0.019400000	2.567894000
H	-2.460518000	0.980873000	2.487920000
H	-2.084251000	-0.716799000	2.778959000
H	-3.577961000	-0.019288000	3.429932000
C	-7.265601000	-1.059335000	0.227995000
H	-7.597212000	-1.439989000	1.200481000
H	-7.619693000	-1.750163000	-0.545140000
H	-7.771549000	-0.098138000	0.064008000
N	-1.541498000	-0.375918000	0.032761000
B	-0.899203000	0.879208000	-0.222290000
B	0.779961000	0.842952000	-0.234488000

N	1.659185000	1.975333000	-0.570240000
N	2.197216000	2.799960000	0.144631000
N	2.735349000	3.623091000	0.730744000
N	1.367936000	-0.435859000	-0.004026000
C	-0.785589000	-1.530897000	0.242214000
C	0.567186000	-1.563499000	0.225399000
H	-1.346061000	-2.440799000	0.424749000
H	1.092796000	-2.496171000	0.393119000
C	2.801477000	-0.661476000	0.014607000
C	3.486058000	-0.629843000	1.241904000
C	3.470330000	-0.921223000	-1.194063000
C	4.850351000	-1.152533000	-1.148402000
C	5.567865000	-1.127650000	0.052879000
C	4.866119000	-0.868946000	1.235172000
H	5.376566000	-1.357729000	-2.078994000
H	5.404461000	-0.848814000	2.180990000
C	2.768763000	-0.337718000	2.540557000
H	3.454170000	-0.432445000	3.388307000
H	1.926438000	-1.019068000	2.707627000
H	2.362027000	0.681218000	2.556433000
C	2.733417000	-0.938636000	-2.512968000
H	3.392068000	-1.277840000	-3.318581000
H	2.369823000	0.062616000	-2.775144000
H	1.860759000	-1.601446000	-2.485478000
C	7.063765000	-1.346490000	0.068909000
H	7.602044000	-0.397841000	-0.061324000
H	7.380232000	-2.014425000	-0.739894000
H	7.395417000	-1.781872000	1.017965000
N	-1.790335000	2.015215000	-0.438528000
N	-1.398853000	3.156501000	-0.683029000
N	-1.181156000	4.251819000	-0.913189000

TS2 (B3LYP/6-31+G*)

C	-2.963351000	-0.476885000	-2.541430000

C	-3.697430000	-0.591505000	-1.225779000
H	-2.138804000	-1.195697000	-2.613983000
H	-3.643622000	-0.654187000	-3.380456000
H	-2.528768000	0.522592000	-2.666574000
C	-3.016141000	-0.504020000	0.000113000
C	-5.085160000	-0.776231000	-1.200229000
C	-5.798469000	-0.867901000	0.000169000
C	-5.085172000	-0.775798000	1.200531000
C	-3.697433000	-0.591058000	1.226025000
H	-5.620662000	-0.849327000	-2.145304000
H	-5.620678000	-0.848554000	2.145628000
C	-2.963370000	-0.475956000	2.541643000
H	-2.528842000	0.523587000	2.666452000
H	-2.138785000	-1.194698000	2.614445000
H	-3.643638000	-0.653010000	3.380723000
C	-7.301588000	-1.032735000	0.000175000
H	-7.645714000	-1.576014000	0.887306000
H	-7.645537000	-1.577985000	-0.885823000
H	-7.805846000	-0.056719000	-0.000973000
N	-1.573384000	-0.346064000	0.000086000
B	-0.963855000	0.942590000	-0.000167000
B	0.772993000	0.833611000	-0.000147000
N	1.124897000	2.143459000	-0.000434000
N	2.920614000	2.443372000	-0.000526000
N	3.730142000	3.204168000	-0.000715000
N	1.337728000	-0.482087000	0.000121000
C	-0.837186000	-1.549962000	0.000340000
C	0.509952000	-1.610496000	0.000355000
H	-1.425889000	-2.459051000	0.000536000
H	1.014107000	-2.570411000	0.000569000
C	2.765435000	-0.683716000	0.000144000
C	3.449521000	-0.776606000	1.226960000
C	3.449417000	-0.777685000	-1.226661000
C	4.834790000	-0.978527000	-1.201108000

C	5.545715000	-1.084838000	0.000196000
C	4.834904000	-0.977465000	1.201459000
H	5.371207000	-1.051002000	-2.145645000
H	5.371401000	-1.049112000	2.146012000
C	2.713274000	-0.655531000	2.541082000
H	3.401466000	-0.780324000	3.383002000
H	1.922834000	-1.409816000	2.633483000
H	2.228940000	0.323416000	2.641566000
C	2.713050000	-0.657725000	-2.540817000
H	3.401204000	-0.782996000	-3.382697000
H	2.228508000	0.321049000	-2.641972000
H	1.922754000	-1.412236000	-2.632615000
C	7.037013000	-1.333640000	0.000230000
H	7.257702000	-2.409859000	-0.000411000
H	7.515210000	-0.903466000	0.887074000
H	7.515439000	-0.902394000	-0.885978000
N	-1.859625000	2.079794000	-0.000394000
N	-1.514450000	3.261379000	-0.000617000
N	-1.345395000	4.386679000	-0.000832000

TS3 (B3LYP/6-31+G*)

C	2.808114000	-0.614818000	2.553286000
C	3.537056000	-0.756479000	1.237237000
H	1.956800000	-1.301606000	2.625698000
H	3.481451000	-0.818384000	3.391966000
H	2.412073000	0.400489000	2.679277000
C	2.856516000	-0.657741000	0.011557000
C	4.919156000	-0.978290000	1.211810000
C	5.628797000	-1.098197000	0.011510000
C	4.916538000	-0.996313000	-1.188240000
C	3.533792000	-0.774551000	-1.213764000
H	5.453290000	-1.059768000	2.157049000
H	5.448463000	-1.091392000	-2.133339000
C	2.802518000	-0.652835000	-2.530550000

H	2.388305000	0.354327000	-2.662989000
H	1.963002000	-1.354467000	-2.599335000
H	3.479153000	-0.849619000	-3.368142000
C	7.126734000	-1.305183000	0.012871000
H	7.463726000	-1.801824000	-0.903646000
H	7.447049000	-1.914402000	0.865684000
H	7.658325000	-0.346135000	0.079913000
N	1.419635000	-0.458185000	0.012770000
B	0.817797000	0.838503000	0.010839000
B	-0.879255000	0.847026000	0.027964000
N	-1.933145000	1.825005000	0.080425000
N	-1.282359000	3.417391000	0.228166000
N	-1.355740000	4.521369000	0.352960000
N	-1.465788000	-0.478461000	-0.016678000
C	0.656101000	-1.635500000	-0.008859000
C	-0.694202000	-1.636680000	-0.030362000
H	1.210665000	-2.565909000	-0.015240000
H	-1.242130000	-2.572649000	-0.055262000
C	-2.904327000	-0.652321000	-0.021876000
C	-3.589386000	-0.690309000	-1.250602000
C	-3.586726000	-0.791688000	1.201334000
C	-4.974262000	-0.971127000	1.169336000
C	-5.688864000	-1.010822000	-0.033509000
C	-4.977089000	-0.871948000	-1.230397000
H	-5.509512000	-1.082097000	2.110857000
H	-5.514343000	-0.905809000	-2.176658000
C	-2.855652000	-0.531307000	-2.561706000
H	-3.540006000	-0.663680000	-3.405727000
H	-2.042552000	-1.259343000	-2.665983000
H	-2.406813000	0.465858000	-2.643292000
C	-2.849871000	-0.738947000	2.519117000
H	-3.536763000	-0.914886000	3.353055000
H	-2.379814000	0.240451000	2.667495000
H	-2.052182000	-1.489380000	2.572396000

C	-7.192250000	-1.170789000	-0.038104000
H	-7.540470000	-1.664875000	-0.951947000
H	-7.692828000	-0.194353000	0.016329000
H	-7.535800000	-1.760888000	0.818868000
N	1.796951000	1.924164000	0.010371000
N	1.677522000	3.125590000	-0.172252000
N	1.728927000	4.257091000	-0.334558000

References

[1] H. Nöth, W. Meister, *Z. Naturforsch., B: Chem. Sci.* **1962**, *17*, 714.

[2] H. Braunschweig, T. Thiess, T. Kupfer, M. Ernst, *Chem. Eur. J.* **2020**, *DOI: 10.1002/chem.201905356*.

[3] W. Fraenk, T. Habereder, A. Hammerl, T. M. Klapötke, B. Krumm, P. Mayer, H. Nöth, M. Warchhold, *Inorg. Chem.* **2001**, *40*, 1334-1340.

[4] G. Sheldrick, *Acta Crystallogr. Sect. A: Found. Crystallogr.* **2015**, *71*, 3-8.

[5] G. Sheldrick, *Acta Crystallogr. Sect. A: Found. Crystallogr.* **2008**, *64*, 112-122.

[6] a) S. H. Vosko, L. Wilk, M. Nusair, *Can. J. Phys.* **1980**, *58*, 1200-1211; b) C. Lee, W. Yang, R. G. Parr, *Physical Review B* **1988**, *37*, 785-789; c) A. D. Becke, *J. Chem. Phys.* **1993**, *98*, 5648-5652; d) P. J. Stephens, F. J. Devlin, C. F. Chabalowski, M. J. Frisch, *J. Phys. Chem.* **1994**, *98*, 11623-11627.

[7] a) T. Clark, J. Chandrasekhar, G. W. Spitznagel, P. V. R. Schleyer, *J. Comput. Chem.* **1983**, *4*, 294-301; b) J. D. Dill, J. A. Pople, *J. Chem. Phys.* **1975**, *62*, 2921-2923; c) R. Ditchfield, W. J. Hehre, J. A. Pople, *J. Chem. Phys.* **1971**, *54*, 724-728; d) P. C. Hariharan, J. A. Pople, *Theoret. Chim. Acta* **1973**, *28*, 213-222; e) W. J. Hehre, R. Ditchfield, J. A. Pople, *J. Chem. Phys.* **1972**, *56*, 2257-2261.

[8] a) R. Bauernschmitt, R. Ahlrichs, *J. Chem. Phys.* **1996**, *104*, 9047-9052; b) R. Seeger, J. A. Pople, *J. Chem. Phys.* **1977**, *66*, 3045-3050.

[9] X. Lu, Z. Su, X. Xu, N. Wang, Q. Zhang, *Chem. Phys. Lett.* **2003**, *371*, 172-177.

[10] R. Krishnan, J. S. Binkley, R. Seeger, J. A. Pople, *J. Chem. Phys.* **1980**, *72*, 650-654.

[11] a) Z. Chen, C. S. Wannere, C. Corminboeuf, R. Puchta, P. v. R. Schleyer, *Chem. Rev.* **2005**, *105*, 3842-3888; b) P. v. R. Schleyer, C. Maerker, A. Dransfeld, H. Jiao, N. J. R. van Eikema Hommes, *J. Am. Chem. Soc.* **1996**, *118*, 6317-6318; c) A. Stanger, *J. Org. Chem.* **2006**, *71*, 883-893.

[12] M. J. Frisch, G. W. Trucks, H. B. Schlegel, G. E. Scuseria, M. A. Robb, J. R. Cheeseman, G. Scalmani, V. Barone, B. Mennucci, G. A. Petersson, H. Nakatsuji, M. Caricato, X. Li, H. P. Hratchian, A. F. Izmaylov, J. Bloino, G. Zheng, J. L. Sonnenberg, M. Hada, M. Ehara, K. Toyota, R. Fukuda, J. Hasegawa, M. Ishida, T. Nakajima, Y. Honda, O. Kitao, H. Nakai, T. Vreven, J. A. Montgomery Jr., J. E. Peralta, F. Ogliaro, M. Bearpark, J. J. Heyd, E. Brothers, K. N. Kudin, V. N. Staroverov, R. Kobayashi, J. Normand, K. Raghavachari, A. Rendell, J. C. Burant, S. S. Iyengar, J. Tomasi, M. Cossi, N. Rega, J. M. Millam, M. Klene, J. E. Knox, J. B. Cross, V. Bakken, C. Adamo, J. Jaramillo, R. Gomperts, R. E. Stratmann, O. Yazyev, A. J. Austin, R. Cammi, C. Pomelli, J. W. Ochterski, R. L. Martin, K. Morokuma, V. G. Zakrzewski, G. A. Voth, P. Salvador, J. J. Dannenberg, S. Dapprich, A. D. Daniels, Ö. Farkas, J. B. Foresman, J. V. Ortiz, J. Cioslowski, D. J. Fox, Gaussian 16, Revision B.01.

[13] G. A. Andrienko, “ChemCraft: graphical software for visualization of quantum chemistry computations,” can be found under https://www.chemcraftprog.com, 2019.

[14] B. O. Roos, in *Advances in Chemical Physics*, Wiley, **2007**, pp. 399-445.

[15] a) C. Angeli, R. Cimiraglia, S. Evangelisti, T. Leininger, J.-P. Malrieu, *J. Chem. Phys.* **2001**, *114*, 10252-10264; b) C. Angeli, R. Cimiraglia, J.-P. Malrieu, *Chem. Phys. Lett.* **2001**, *350*, 297-305; c) C. Angeli, R. Cimiraglia, J.-P. Malrieu, *J. Chem. Phys.* **2002**, *117*, 9138-9153.

[16] T. H. Dunning, *J. Chem. Phys.* **1989**, *90*, 1007-1023.

[17] a) M. Nakano, *Top. Curr. Chem.* **2017**, *375*, 47; b) K. Yamaguchi, *Chem. Phys. Lett.* **1975**, *33*, 330-335; c) S. Yamanaka, M. Okumura, M. Nakano, K. Yamaguchi, *J. Mol. Struct.* **1994**, *310*, 205-218.

[18] F. Neese, *Wiley Interdisciplinary Reviews: Computational Molecular Science* **2012**, *2*, 73-78.
[19] a) K. Fukui, *Acc. Chem. Res.* **1981**, *14*, 363-368; b) H. P. Hratchian, H. B. Schlegel, in *Theory and Applications of Computational Chemistry* (Eds.: C. E. Dykstra, G. Frenking, K. S. Kim, G. Scuseria), Elsevier, Amsterdam, **2005**, pp. 195-249.
[20] S. Grimme, J. Antony, S. Ehrlich, H. Krieg, *J. Chem. Phys.* **2010**, *132*, 154104.
[21] A. V. Marenich, C. J. Cramer, D. G. Truhlar, *J. Phys. Chem. B* **2009**, *113*, 6378-6396.
[22] a) N. Sieffert, M. Bühl, *Inorg. Chem.* **2009**, *48*, 4622-4624; b) A. Poater, E. Pump, S. V. C. Vummaleti, L. Cavallo, *J. Chem. Theory Comput.* **2014**, *10*, 4442-4448.
[23] R. L. Martin, P. J. Hay, L. R. Pratt, *J. Phys. Chem. A* **1998**, *102*, 3565-3573.
